# Supplementary material for: Dipeptide coacervates as artificial membraneless organelles for bioorthogonal catalysis
Source: Nat Commun. 2024 Jan 2;15:39. doi: 10.1038/s41467-023-44278-9 (PMC10761997; doi:10.1038/s41467-023-44278-9)
Supplement: Supplementary file 1 — Supplementary Information [file 41467_2023_44278_MOESM1_ESM.pdf]

Supplementary Information

Dipeptide Coacervates as Artificial Membraneless Organelles for Bioorthogonal Catalysis

Shoupeng Cao[1], Tsvetomir Ivanov[1], Julian Heuer[1], Calum T. J. Ferguson[1,2], Katharina Landfester[1], and Lucas Caire da Silva[1,3]**

[1]Max Planck Institute for Polymer Research, 55128 Mainz, Germany

[2]Department School of Chemistry, University of Birmingham, Birmingham B15 2TT, United Kingdom

[3]Department of Chemistry, McGill University, Montreal, H3A 0B8, Canada

**e-mail: silva@mpip-mainz.mpg.de; landfester@mpip-mainz.mpg.de*

1. Supplementary Methods

1.1 Materials

Boc-*L*-phenylalanine (Boc-Phe-OH, 99%, Sigma), *L*-Phenylalanin-methylester-hydrochlorid (98%, Sigma), *L*-glutamic acid (99%, Sigma), N-(tert-Butoxycarbonyl)-*L*-leucine monohydrate (99%, Sigma), N-(tert-Butoxycarbonyl)-*L*-methionine (98.0 %, TCI), *L*-methionine methyl ester (99%, Sigma), isobutylamin (99%, Sigma), (S)-(+)-2-Amino-N-methyl-3-phenyl-propionamide hydrochloride (98%, Sigma), thionyl chloride (99.5%, Sigma), 2-(2-Naphthyl)-ethylamin-hydrochlorid (97%, Sigma), L-glutamic acid (99%, Sigma), glycine methyl ester hydrochloride (99%, Sigma), N-(tert-Butoxycarbonyl)-L-leucine monohydrate (98%, Sigma), allyl chloroformate (97%, Sigma), 1-hydroxybenzotriazole (HOBt, 97%, Sigma), 2-(1H-benzotriazole-1-yl)-1,1,3,3-tetramethyluronium tetra fluoroborate (HBTU, 89%, Sigma), *N,N*-diisopropylethylamine (DIPEA, 99%, Sigma), pyridine (99.8%, Sigma), $KMnO_4$ (99%, Sigma), sodium thiosulfate (99%, Sigma), hydrogen peroxide (35 wt% solution, Sigma), 4 M hydrogen chloride solution in dioxane (TCI), bromotris(triphenylphosphine)copper(I) ($Cu(PPh_3)_4Br$, 98%, Sigma), 3-azido-7-hydroxycoumarin (98%, TCI), phenylacetylene (98%, Sigma), bis-N-succinimidyl-(pentaethylene glycol) ester (Bis(NHS)PEG_5, (Sigma), 2,1,3-benzothiadiazole-4,7-bis(boronic acid pinacol ester) (95%, Sigma), 2-bromothiophene (98%, Sigma), tetrakis(triphenylphosphane)palladium (97%, Sigma), di(N-succinimidyl)-glutarat (Bis-NHS-C3, (Sigma), chloro-(cyclooctadien)-(pentamethylcyclopentadienyl)-ruthenium(II) (Sigma), resorufin sodium salt (Sigma), Rhodamine 110 chloride (>75%,Sigma), Nile Red (98%, Sigma), methylene blue (Sigma), Rhodamine 6G (95%, Sigma), thioflavin T (>65%, Sigma), Zinc (II) Protoporphyrin IX (92%, Sigma), calcein (mixed isomers, Sigma), chlorpromazine (98%, Sigma), amiloride hydrochloride (98%, Sigma), methyl-β-cyclodextrin (Sigma), NaN_3 (TCI), Bovine serum albumin (BSA, Sigma), albumin from bovine serum FITC conjugate (FITC-BSA, Thermofisher), horseradish peroxidase (HRP, Sigma), glucose oxidase from aspergillus niger (GOX, Sigma), amplex™ red reagent (Thermo Fisher). LysoTracker™ Green DND-26 (Thermo Fisher), CellMask™ Deep Red Plasma Membrane Stain (Thermo Fisher). $CDCl_3$ (99.9% D, Sigma-Aldrich), DMSO-d_6 (99.9% D, Sigma-Aldrich), HEPES buffer solution (1 M, Sigma), phosphate buffered saline (PBS, sigma). Grace Bio-Labs reusable CultureWell™ gaskets (Sigma). All the other solvents, chemicals, and salts used were purchased from Sigma Aldrich and were used as received unless otherwise stated.

1.2 Instrumentation

Nuclear magnetic resonance (NMR) spectroscopy: Routine proton nuclear magnetic resonance (^1H NMR) measurements were performed on a Bruker Avance 300 MHz Ultrashield™ spectrometer equipped with a Bruker SampleCase autosampler, using $CDCl_3$ or DMSO-d_6 as solvent and TMS as internal standard.

Microplate reader: Catalytic reactions were evaluated using a microplate reader (TECAN, infinite M1000). Reaction progress was monitored by the absorbance or fluorescence signal on the microplate reader (TECAN).

UV-Vis spectroscopy: UV-Vis spectra were characterized using UV-Vis spectroscopy (Agilent Cary 60 instrument).

Mass spectra: The molecular weights of the peptide compounds were measured using an Advion Express CMS compact mass spectrometer. The resulting spectra were analyzed using Advion CheMS Express software version 5.1.0.2. High-resolution ESI mass spectra were obtained using a Waters SYNAPT G2-Si spectrometer.

Wide-field fluorescence microscopy imaging: The wide-field fluorescence images were captured using Leica DMi8 inverted microscope.

Confocal Scanning Microscopy Imaging: Confocal microscopy was performed using a Leica TCS 264 SP5X system.

1.3 Synthesis procedures

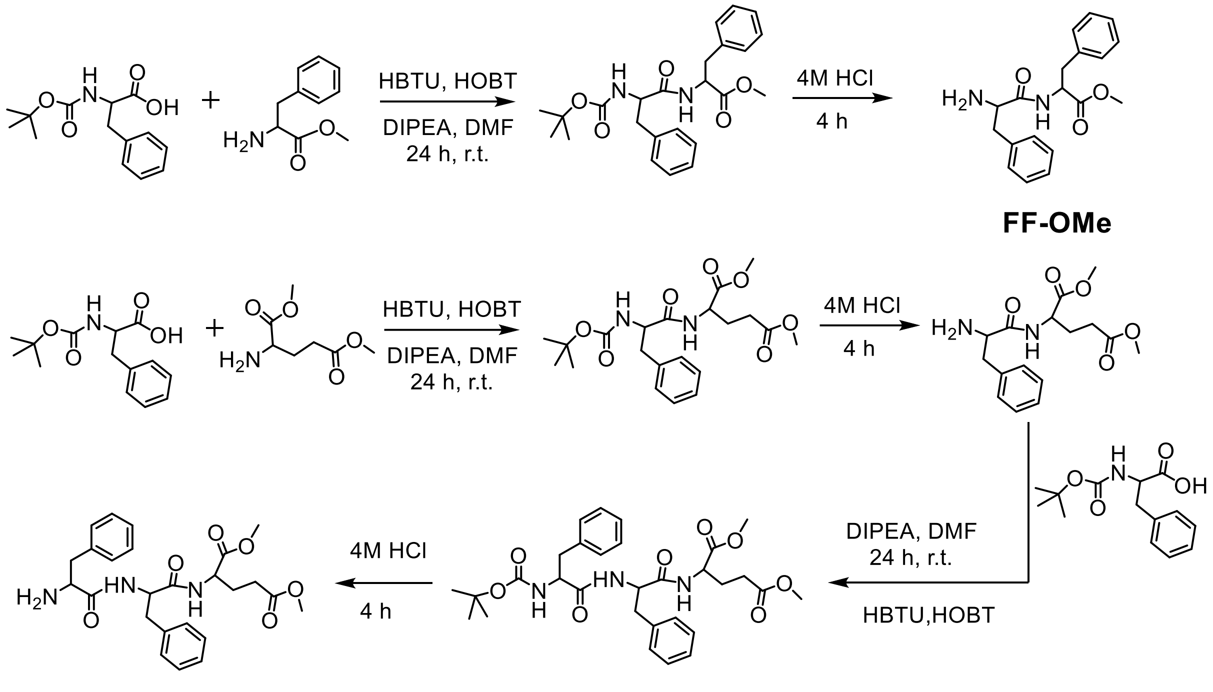

Supplementary Figure 1: Synthesis route of derivatives of diphenylalanine-based compounds.

Derivatives of diphenylalanine-based compounds were synthesized via simple multi-step reactions according to literature reports with slight modifications.[1] The typical synthesis of FF-OMe and FF-GLU is illustrated below. Other diphenylalanine compounds were synthesized using similar procedures.

FF-OMe: This compound was synthesized in a two-step reaction.

(**i**) First, N-(tert-butoxycarbonyl)-*L*-phenylalanine (Boc-Phe-OH) (2.9 g, 10.9 mmol), HBTU (4.13 g 10.9 mmol) and HOBt (1.47 g, 10.9 mmol) were dissolved in DMF (15 mL) in a round-bottom flask and the mixture was stirred with a magnetic stirrer. N,N-Diisopropylethylamine (DIPEA) (3.5 mL, 21.8 mmol), and L-phenylalanin-methylester-hydrochlorid (2.12 g, 9.9 mmol) were added at 1 minute intervals respectively and the reaction mixture was stirred for 24 h at room temperature. The reaction mixture was poured into 500 mL of water. The white precipitate was collected by filtration and washed with water. The crude product was dried in an oven at 40 ^{o}C overnight to give 1.7 g of white solid, which was characterized by NMR. ^{1}H NMR (300 MHz, $CDCl_3$) δ 7.38 – 6.93 (m, 10H), 6.28 (d, *J* = 7.6 Hz, 1H), 4.94 (s, 1H), 4.80 (q, *J* = 6.3 Hz, 1H), 4.34 (d, *J* = 8.2 Hz, 1H), 3.69 (s, 3H), 3.05 (q, *J* = 6.3 Hz, 4H), 1.42 (s, 9H). ^{13}C NMR (75 MHz, $CDCl_3$) δ 171.33, 170.72, 136.49, 135.63, 129.37, 129.22, 128.68, 128.55, 127.12, 126.99, 77.44, 77.02, 76.60, 53.26, 52.26, 38.27, 37.98, 28.23.

(**ii**) The intermediate compound (1.2 g) was dissolved in 6 mL of 4 M hydrogen chloride solution in dioxane for protection. After stirring for 3 hours, the solvent was evaporated on a rotary

evaporator to give an oily residue. Diethyl ether was added to the flask and the contents were stirred gently. A white precipitate formed and was separated by centrifugation, yielding about 1.0 g of a white product. The product was characterized by NMR and Ms. ^{1}H NMR (300 MHz, DMSO) δ 9.26 (d, *J* = 7.5 Hz, 1H), 8.29 (s, 3H), 7.36 – 7.16 (m, 11H), 4.53 (m, 1H), 4.08 (s, 1H), 3.59 (s, 3H), 3.16 (dd, *J* = 14.0, 5.6 Hz, 1H), 3.09 – 2.91 (m, 3H). ^{13}C NMR (75 MHz, DMSO) δ 171.12, 168.15, 136.76, 134.75, 129.59, 129.10, 128.40, 128.33, 127.06, 126.68, 117.68, 53.94, 53.10, 51.97, 36.69, 36.55. MALDI MS(EI): calculated for $C_{29}H_{22}N_2O_3$: 326.1630 (M^+); found 327.1834 ($M+H^+$).

FF-Glu: This compound was synthesized in a four-step reaction.

(**i**) Dimethyl glutamate was synthesized according to the literature.[2] N-(tert-butoxycarbonyl)-*L*-phenylalanine (Boc-Phe-OH) (2.9 g, 10.9 mmol), HBTU (4.16 g 10.9 mmol) and HOBt (1.50 g, 10.9 mmol) were dissolved in DMF (15 mL) in a round-bottom flask and the mixture was stirred with a magnetic stirrer. N,N-Diisopropylethylamine (DIPEA) (3.5 mL, 21.8 mmol), and dimethyl glutamate (1.75 g,10 mmol) were added at 1 minute intervals and the reaction mixture was stirred for 24 h at room temperature. The reaction mixture was poured into 500 mL of water. The white precipitate was collected by filtration and washed with water. The crude product was dried in an oven at 40 ^{o}C overnight to give 1.2 g of a white solid which was characterized by NMR. ^{1}H NMR (300 MHz, $CDCl_3$) δ 7.34 – 7.28 (m, 2H), 7.27 – 7.18 (m, 3H), 6.54 (d, *J* = 7.7 Hz, 1H), 4.97 (s, 1H), 4.58 (m, 1H), 4.36 (d, *J* = 7.4 Hz, 1H), 3.73 (s, 3H), 3.68 (s, 3H), 3.15 – 3.01 (m, 2H), 2.41 – 1.89 (m, 4H), 1.43 (s, 9H). ^{13}C NMR (75 MHz, $CDCl_3$) δ 173.10, 171.64, 171.18, 136.41, 129.34, 128.69, 126.99, 52.51, 51.80, 51.60, 38.11, 29.74, 28.22, 27.33.

(**ii**) The intermediate compound (0.7 g) was dissolved in 4.5 mL of 4 M hydrogen chloride solution in dioxane for protection. After stirring for 3 h, the solvent was evaporated on a rotary evaporator to give an oily residue. Diethyl ether was added to the flask and the contents were stirred gently. A white precipitate formed and was separated by centrifugation, giving about 0.58 g of a white product. The product was characterized by NMR spectroscopy. ^{1}H NMR (300 MHz, $CDCl_3$) δ 8.36 (s, 2H), 8.01 (d, *J* = 7.2 Hz, 1H), 7.38 (d, *J* = 7.3 Hz, 2H), 7.30 (d, *J* = 5.6 Hz, 3H), 4.67 (s, 1H), 4.47 (d, *J* = 6.7 Hz, 1H), 3.67 (s, 3H), 3.57 (s, 3H), 3.48 – 3.26 (m, 3H), 2.45 (t, *J* = 7.4 Hz, 2H), 2. 29 – 2.17 (m, 2H). ^{13}C NMR (75 MHz, $CDCl_3$) δ 173.69, 171.23, 168.36, 134.14, 129.94, 128.79, 127.52, 54.66, 52.49, 52.14, 51.97, 37.09, 30.18, 26.78.

(**iii**) Then, N-(tert-butoxycarbonyl)-*L*-phenylalanine (Boc-Phe-OH) (0.7 g, 2.6 mmol), HBTU (1.0 g 2.6 mmol) and HOBt (0.36 g, 2.6 mmol) were dissolved in DMF (5 mL) in a round-bottom flask and the mixture was stirred with a magnetic stirrer. N,N-Diisopropylethylamine (DIPEA) (3.5 mL, 21.8 mmol), and the above intermediate (0.87 g, 2,4 mmol) were added at 1 min intervals and the reaction mixture was stirred for 24 h at room temperature. The reaction mixture was poured into 100 mL of water. The white precipitate was collected by filtration and

washed with water. The crude product was dried in an oven at 40 °C overnight to give 0.96 g of white solid. The product was characterized by NMR. ^{1}H NMR (300 MHz, $CDCl_3$) δ 7.38 – 7.30 (m, 2H), 7.27 – 6.96 (m, 8H), 6.57 (s, 1H), 6.40 (d, *J* = 7.8 Hz, 1H), 4.83 (s, 1H), 4.64 (m, 1H), 4.50 (m, 1H), 4.32 (m, 1H), 3.73 (s, 3H), 3.67 (s, 3H), 3.23 – 2.86 (m, 5H), 2.40 – 2.09 (m, 3H), 1.93 (m, 1H), 1.35 (s, 9H). ^{13}C NMR (75 MHz, $CDCl_3$) δ 173.08, 171.40, 170.96, 170.28, 136.28, 136.07, 129.30, 129.26, 128.82, 128.72, 128.54, 127.18, 127.12, 118.16, 80.55, 54.09, 52.47, 51.78, 29.84, 28.16, 27.00.

(**iv**) The above intermediate compound (1.1 g) was then dissolved in 6 mL of 4 M hydrogen chloride solution in dioxane for protection. After stirring for 3 h, the solvent was evaporated on a rotary evaporator to give an oily residue. Diethyl ether was added to the flask and the contents were stirred gently. A white precipitate formed which was separated by centrifugation to give 0.99 g of white solid. The product was characterized by NMR and GC-MS. ^{1}H NMR (300 MHz, DMSO) δ 8.92 (d, *J* = 8.1 Hz, 1H), 8.66 (d, *J* = 7.7 Hz, 1H), 8.19 – 8.04 (m, 2H), 7.42 – 7.15 (m, 10H), 4.60 (m, 1H), 4.33 (m, 1H), 4.01 (s, 1H), 3.62 (s, 3H), 3.59 (s, 3H), 3.19 – 2.80 (m, 4H), 2.47 – 2.31 (m, 2H), 2.12 – 1.76 (m, 2H). ^{13}C NMR (75 MHz, DMSO) δ 172.53, 171.70, 170.74, 167.89, 137.31, 134.75, 129.62, 129.21, 128.40, 128.13, 127.03, 126.40, 72.13, 70.49, 60.14, 54.12, 53.10, 51.95, 51.37, 37.47, 36.74, 29.53, 26.00. MALDI MS(EI): calculated for $C_{25}H_{31}N_3O_6$: 469.2213 (M^+); found 470.2747 ($M+H^+$).

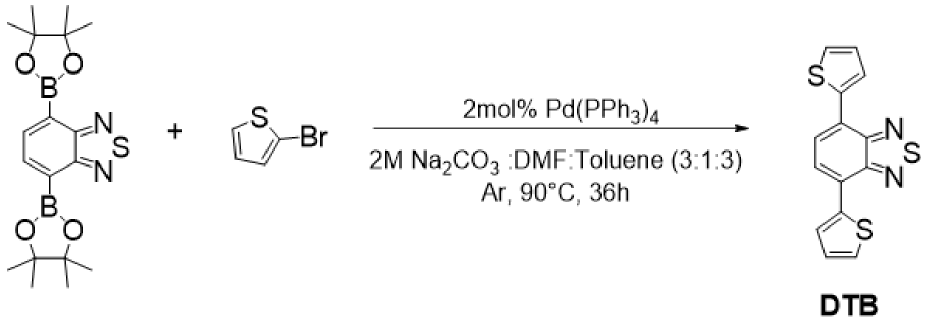

Supplementary Figure 2: Synthesis route of the photocatalytic species 4,7-di(2-thienyl)-2,1,3-benzothiadiazole (abbreviated as DTB).

A 150 mL Schlenk tube with stirring rod was filled with 25 mL 2 M Na_2CO_3 aq. solution, 25 mL toluene and 9 mL DMF. The solution was then degassed with an argon stream for 15 min, and 4,7-bis(4,4,5,5-tetramethyl-1,3,2-dioxaborolan-2-yl)-2,1,3-benzothiadiazole (1.0 eq, 1.0 g, 2.58 mmol) and 2-bromothiophene (2.5 eq., 1.05 g, 6.44 mmol) and tetrakis(triphenylphosphane)palladium (0.02 eq., 59.55 mg, 51.53 µmol) were added in the presence of an argon flow. The reaction mixture was heated to 90 °C for 36 h with an attached reflux condenser while stirring vigorously. After cooling to room temperature, 30 mL of Milli-Q water was added, followed by extraction with dichloromethane (4 x 25 mL), washing with brine, and drying over Na_2SO_4. After evaporation of all volatiles with a rotary evaporator, the crude mixture was purified by SiO_2 column chromatography (gradient from 10% DCM: 90%

petroleum ether to 70% DCM: 30% petroleum ether). The product was obtained as a red powder (310 mg, 40% yield).^{1}H NMR (400 MHz, DMSO-d_6) δ 8.23 (dd, J = 3.7, 1.2 Hz, 2H), 8.17 (s, 2H), 7.83 (dd, J = 5.1, 1.1 Hz, 2H), 7.34 (dd, J = 5.1, 3.7 Hz, 2H). ^{13}C NMR (101 MHz, DMSO-d6) δ 152.20, 138.87, 128.76, 128.57, 127.91, 126.34, 125.52.

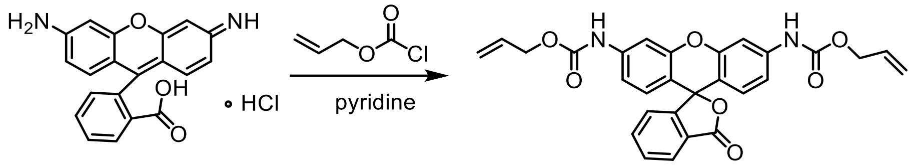

Supplementary Figure 3: Synthesis route of caged Rhodamine 110

This compound was synthesized according to the literature using similar procedures. [3] Briefly, rhodamine 110 (100 mg, 0.27 mmol) was dissolved in dry DMF (1 mL) in a 10 mL pear-shaped flask equipped with a magnetic stirring bar. Pyridine (65 μL, 0.80 mmol) was then added and the mixture was cooled to 0 °C. The flask was then purged with nitrogen. Finally, allyl chloroformate (57 μL, 0.53 mmol) was added dropwise to the above solution. The reaction was then allowed to warm to room temperature and stirred overnight. The reaction mixture was then diluted with ethyl acetate (20 mL) and washed twice with aqueous hydrochloric acid (5%, 10 mL) and with aqueous sodium bicarbonate solution (saturated, 2 × 10 mL). The organic phase was dried with sodium sulfate and filtered. The solution was then concentrated with a rotavap and purified by flash chromatography using 1:1 hexanes/ethyl acetate to give a solid product (40% yield). ^{1}H NMR (400 MHz, DMSO) δ 10.09 (s, 2H), 8.02 (d, *J* = 7.5 Hz, 1H), 7.76 (m, 2H), 7.58 (d, *J* = 2.1 Hz, 2H), 7.28 (d, *J* = 7.7 Hz, 1H), 7.23 – 7.13 (m, 2H), 6.71 (d, *J* = 8.6 Hz, 2H), 5.99 (m, 2H), 5.38 (d, *J* = 17.2 Hz, 2H), 5.26 (d, *J* = 10.5 Hz, 2H), 4.64 (d, *J* = 5.5 Hz, 4H). ^{13}C NMR (101 MHz, DMSO) δ 169.17, 153.58, 152.98, 151.42, 141.92, 136.21, 133.51, 130.70, 129.00, 126.19, 125.26, 124.44, 118.30, 114.97, 112.94, 105.60, 82.44, 65.44, 40.63, 40.58, 40.43, 40.38, 40.22, 40.17, 40.01, 39.96, 39.80, 39.75, 39.54, 39.33.

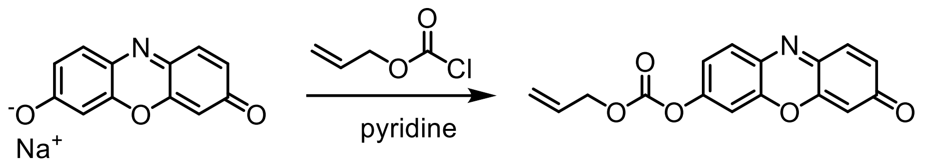

Supplementary Figure 4: Synthesis route of caged resorufin

This compound was synthesized according to the literature using similar procedures.[3] In detail, resorufin sodium salt (200 mg, 1.05 mmol) was dissolved in dry DMF (1 mL) in a 10 mL pear-shaped flask equipped with a magnetic stirring bar. Pyridine (0.18 mL, 2.1 mmol) was then added and the mixture was cooled to 0 °C. The flask was then purged with nitrogen. Finally, allyl chloroformate (0.12 mL, 1.1 mmol) was added dropwise to the above solution. The reaction was then allowed to warm to room temperature and stirred overnight. The reaction

mixture was then diluted with ethyl acetate (50 mL) and washed twice with aqueous hydrochloric acid (5%) and with aqueous saturated sodium bicarbonate solution. The organic phase was dried with sodium sulfate and filtered. The solution was then concentrated with a rotavap and purified by flash chromatography using 4:1 hexanes/ethyl acetate to give a solid product (20% yield). ^{1}H NMR (400 MHz, $CDCl_3$) δ 7.74 (d, *J* = 8.7 Hz, 1H), 7.37 (d, *J* = 9.8 Hz, 1H), 7.19 – 7.06 (m, 2H), 6.80 (d, *J* = 10.0 Hz, 1H), 6.27 (s, 1H), 5.94 (m, 1H), 5.48 – 5.27 (m, 2H), 4.77 – 4.61 (m, 2H). ^{13}C NMR (101 MHz, $CDCl_3$) δ 186.30, 153.58, 152.42, 149.29, 148.46, 144.37, 135.24, 134.83, 131.32, 131.26, 130.63, 120.20, 118.60, 109.13, 107.38, 69.77.

FITC/RITC labeling with GOX and HRP: Enzyme labeling was performed as described in the literature with slight modifications.[2] Briefly, 10 mg of GOX or HRP was dissolved in 2 mL of sodium carbonate buffer (100 mM, pH 8.5). Then 20 μL FITC/RITC solution (1.0 mg mL^{-1} in DMSO) was slowly added to the above solution. The mixture was stirred at r.t. for 3 h in the dark, followed by purification by dialysis (MWCO 12-14 KDa) against Milli-Q water for 2 days and stored at 4°C until further use.

Synthesis of quaternized amylose (Q-AM) and carboxy-functionalized amylose (C-AM): The synthesis of the pairs for complex coacervation was performed according to a literature report with slight modifications.[4] Briefly, quaternized amylose (Q-AM) was prepared by dissolving 1.5 g amylose and 2.8 g NaOH in 14 mL Milli-Q at 30°C. After complete dissolution of the amylose, 11.6 mL of 3-chloro-2-hydroxypropyltrimethylammonium chloride solution (60% w/v in water) was added dropwise to the stirred reaction mixture and allowed to react overnight. After this time, the mixture was neutralized with acetic acid and precipitated in 200 mL cold ethanol. The resulting precipitate was redissolved in Milli-Q water and dialyzed extensively against water using regenerated cellulose dialysis tubing (Spectrum Labs, USA) with a 3.5 KDa MWCO before lyophilization. ^{1}H NMR (D_2O) characterization data and chemical structures are shown in Fig. S20.

Carboxyl-functionalized amylose (C-AM) was prepared by dissolving 1.5 g amylose and 3.6 g NaOH in 15 mL Milli-Q at 70 °C. After complete dissolution of the amylose, 2.7 g chloroacetic acid was added and the reaction mixture was stirred for 2 h. After the reaction, the mixture was neutralized with acetic acid and precipitated in 200 mL cold ethanol. The resulting precipitate was redissolved in Milli-Q water and dialyzed extensively against water using regenerated cellulose dialysis tubing (Spectrum Labs, USA) with a 3.5 KDa MWCO before lyophilization. ^{1}H NMR (D_2O) characterization data and chemical structures are shown in Fig. S21.

Synthesis of BSA@MnO_2 nanoparticles: The synthesis of BSA-stabilized MnO_2 nanoparticles was performed according to the reported literature with slight modifications.[5] Briefly, a 4 mL solution of $KMnO_4$ (5 mM) was added to a 25 mL round-bottom flask, which was purged with N_2 and bubbled for 30 min at RT. An 8 mL solution of $Na_2S_2O_3$ (1.875 mM)

was then added at a rate of 1 mL min^{-1} using a syringe pump. The mixed solution was stirred for 10 minutes. Then 3 mL BSA solution (7 mg mL^{-1}) was added dropwise. The solution was stirred for another hour and dialyzed against MilliQ (Spectrapor, MWCO: 1000 KDa) to remove excess BSA. Samples were stored in the refrigerator at 4 °C prior to use.

RITC labeling of BSA@MnO_2 nanoparticles: Briefly, 500 μL of BSA@MnO_2 nanoparticle solution was dispersed in PBS buffer (pH 8.5). 20 μL RITC (1 mg mL^{-1} in DMSO) was added, and the reaction mixture was stirred at r.t. for 3 h. Then, the RITC-labeled nanoparticles were recovered by dialysis (MWCO 12-14 KDa) against Milli-Q water for 2 days and stored at 4°C for further use.

1.4 Peptide droplet formulations and guest cargo encapsulation

Preparation of peptide coacervates: As an example of coacervate droplet formation from FF-OMe, the FF-OMe solid was dissolved in 5 mM HEPES buffer (pH~6) at a concentration of 20 mg mL^{-1}. For microscopic imaging of the coacervates, 10 μL of peptide coacervates was added with 0.1 M NaOH solution to reach a pH above 7. The solution immediately turned milky. 0.5 μL Pluronic® F-108 (1% wt) was added to increase the stability of the coacervates. The formation of coacervate droplets was confirmed using a Leica DMi8 inverted microscope.

Turbidity measurement: All turbidity-based titrations were performed on a Tecan multimode plate reader. Turbidity was used as an indicator of the phase separation of samples where droplet formation was confirmed by light microscopy. Absorbance at 600 nm was used as the wavelength for all turbidity measurements and all measurements were performed at room temperature. After sample addition and shaking for 5 s, the turbidity value was recorded. A well containing the same volume of buffer was used as a blank.

Protein and enzyme encapsulation: All experiments were performed at room temperature unless stated otherwise. All protein and enzyme solutions were stored at -4 °C or -20 °C prior to use. Specifically, 10 μL of the prepared coacervate solution (10 mg mL^{-1}) was mixed with 0.5 μL FITC-BSA (0.5 mg mL^{-1}) or FITC-GOX (0.5 mg mL^{-1}) or RITC-HRP (0.5 mg mL^{-1}) solution, and encapsulation was further confirmed by confocal microscopy using the Leica TCS 264 SP5X system.

Guest molecule partitioning: All experiments were performed at room temperature unless stated otherwise. Briefly, the coacervate droplet solution was first prepared by adding 0.1 M NaOH solution to the peptide solution in HEPES buffer. Then, 10 μL of the prepared coacervate solution (10 mg mL^{-1}) was mixed with the 0.2 μL dye solutions (2 mg mL^{-1} in DMSO or Milli-Q) by pipetting. The mixture was then dropped onto a glass surface with a coverslip using a homemade setup. The droplets were then imaged by confocal microscopy using the Leica TCS 264 SP5X system.

Dimerization of peptide coacervates: Briefly, 200 μL peptide coacervate solution (5 mg mL^{-1}) in a HEPES/PBS buffer (pH~8) was treated with 2 μL Bis(NHS)PEG5 (100 mg mL^{-1} in DMSO) under shaking (300 rpm) for 0.5 h. The dimerized peptide coacervates were then recovered by low-speed centrifugation (3000 rpm) for 1 min. The supernatant was discarded, and fresh PBS buffer was added. Dimerization efficiency was checked by imaging droplets after dilution.

1.5 Biomimicry application with peptide droplets

Enzymatic metabolism with peptide coacervates: Enzymatic reactions with peptide coacervates were determined by microplate reader assay and confocal imaging.

(i) Microplate reader measurements: 200 μL peptide coacervate solution (5 mg mL^{-1}) in HEPES/PBS buffer (pH~8) was mixed with 5 μL HRP solution (0.004 mg mL^{-1}). The mixture was first treated with 0.5 μL Amplex-red (20 mg mL^{-1}). Then 5 μL H_2O_2 was added and the emission intensity at λ_{em}=580 nm was recorded using a microplate reader.

(ii) Confocal imaging measurements: 20 μL peptide coacervate solution (10 mg mL^{-1}) in HEPES/PBS buffer (pH~8) was mixed with 1 μL HRP solution (0.004 mg mL^{-1}). The mixture was first treated with 0.1 μL Amplex-red (20 mg mL^{-1}). Then 1 μL H_2O_2 was added and the emission intensity at λ_{em}=580 nm was recorded by confocal imaging.

Photocatalytic dye degradation with peptide coacervates: 2 μL DTB (20 mg mL^{-1}) was first mixed with peptide coacervate solution (5 mg mL^{-1}, 800 μL) by pipetting. Then 10 μL of 2 mg mL^{-1} methylene blue or rhodamine B was added to the mixture and stirred under dark conditions until equilibrium was reached (5 min). The mixture was then exposed to blue LED light (power: 0.36 W cm^{-2}, λ > 420 nm) for the desired time. 70 μL samples were taken from the solution and subjected to high centrifugation (12000 rpm, 5 min) to dissolve the coacervates. The change in dye concentration was monitored by UV-vis spectroscopy.

Bio-orthogonal uncaging reaction with alloc-protected rhodamine 110 using peptide coacervates: The bio-orthogonal catalytic potential of peptide coacervates was evaluated using the allylcarbamate cleavage of alloc-protected rhodamine 110 with microplate reader assay and confocal imaging study.

(i) Microplate reader measurements: To a 200 μL peptide coacervate solution (5 mg mL^{-1}), 0.5 μL [Cp*Ru(cod)Cl] (Cp* = pentamethylcyclopentadienyl, cod = 1,5-cyclooctadiene, abbreviated as Ru, 2 mg mL-1 in DMSO) was added by pipetting. After equilibration for 2 minutes, 0.5 μL caged Rhodamine 110 (20 mg mL^{-1} in DMSO) was added to the mixture. The solution was mixed by pipetting for a few seconds. The change in fluorescence intensity (λ_{em}=525 nm) was monitored with a microplate reader under periodic shaking.

(ii) Confocal imaging measurements: 20 μL peptide coacervate solution (10 mg mL^{-1} in HEPES/PBS buffer, pH ~8) was mixed with 0.2 μL Ru solution (2 mg mL^{-1} in DMSO). The mixture was then treated with 0.2 μL caged Rhodamine 110 (2 mg mL^{-1} in DMSO). The emission intensity at λ_{em}=525 nm was then recorded by confocal imaging.

Bio-orthogonal uncaging reaction with alloc-protected resorufin using peptide coacervates:

(i) Microplate reader measurements: To a 200 μL peptide coacervate solution (5 mg mL^{-1}), 0.5 μL [Cp*Ru(cod)Cl] (Cp* = pentamethylcyclopentadienyl, cod = 1,5-cyclooctadiene, abbreviated as Ru, 2 mg mL^{-1} in DMSO) was added by pipetting. After equilibration for 2 min, 0.5 μL caged resorufin (4 mg mL^{-1} in DMSO) was added to the mixture. The solution was mixed by pipetting for a few seconds. The change in fluorescence intensity (λ_{em}=585 nm) was monitored with a microplate reader under periodic shaking.

(ii) Confocal imaging measurements: 20 μL peptide coacervate solution (10 mg mL^{-1} in HEPES/PBS buffer, pH ~8) was mixed with 0.2 μL Ru solution (0.5 mg mL^{-1} in DMSO) by pipetting. The mixture was then treated with 0.2 μL caged resorufin (0.5 mg mL^{-1} in DMSO). The emission intensity at λ_{em}=585 nm was then recorded by confocal imaging.

1.6 Peptide coacervates as sub-organelles within complex coacervate-based synthetic cells

Formation of membrane-bound complex coacervates: The formation of membraneless complex coacervates was adopted with slight modifications from the literature report, which utilized the complex coacervation between Q-Am and C-Am, after which the membraneless complex coacervates were stabilized with BSA@MnO_2 nanoparticles. Briefly, Q-Am and CM-Am were dissolved in PBS buffer at a concentration of 2.5 mg mL^{-1}. Coacervation was induced by mixing the solutions of Q-AM and C-AM in a ratio of 1:1. Different volumes of BSA@MnO_2 nanoparticles were then added to the solution by pipetting. The stability of the BSA@MnO_2 stabilized complex coacervate was then checked by optical microscopy.

Integration of peptide coacervates as sub-organelle: 10 µL of peptide coacervates (20 mg mL^{-1}) were first formed by adding small amounts of 1 M NaOH solution. Then 10 µL Q-AM (2.5 mg mL^{-1} in PBS, pH~9) was added and mixed by pipetting for ~10 s. Then 10 µL C-AM (2.5 mg mL^{-1} in PBS, pH~9) was added and mixed by pipetting for ~10 s. A solution of 5 µL BSA@MnO2 (~0.7 mg mL^{-1}) was then added by pipetting for ~10 s. The resulting complex was then subjected to light microscopy to observe the multi-compartmentalized structure.

Bio-orthogonal uncaging reaction in multi-compartment system: The preparation procedures are similar to those above, except that prior to encapsulation in complex coacervates as organelles, the peptide coacervates were treated with 0.2 µL Ru (2 mg mL^{-1} in DMSO). After the formation of the multi-compartment structure, the mixture was treated with 0.2 µL caged Rhodamine 110 (2 mg mL^{-1} in DMSO). Fluorescence emission from the uncaged product was then monitored by confocal microscopy.

1.7 Peptide coacervates as catalytic sub-organelles inside living cells

Toxicity Studies: The cell biocompatibility of the dimerized peptide coacervates was evaluated using a standard MTT assay and a live/dead cell staining assay.

(i) MTT assay: HeLa cells were cultured in DMEM medium containing 10% FBS, 1% penicillin/streptomycin (complete DMEM) in 5% CO_2 at 37°C. Relative cell viability was determined in vitro by the MTT assay. Cells were seeded in 96-well plates at a density of 5 × 103 cells per well in 100 µL complete DMEM medium and cultured at 37°C for 24 hours. The cells were then incubated with the corresponding dimerized peptide coacervates at different concentrations for 2 h each. The cells were then washed and fresh medium containing MTT was added to each plate. The cells were incubated for another 4 h. After removing the medium containing MTT, dimethyl sulfoxide (100 µL) was added to each well to dissolve the formazan crystals. Finally, the plate was gently vortexed for 5 minutes and the absorbance at 490 nm was recorded using a microplate reader.

(ii) Live/dead cell staining assay: HeLa cells were cultured in DMEM medium containing 10% FBS, 1% penicillin/streptomycin (complete DMEM) in 5% CO_2 at 37°C. Relative cell viability was determined in vitro by the MTT assay. Cells were seeded in 96-well plates at a density of 5 × 103 cells per well in 100 µL complete DMEM medium and cultured at 37°C for 24 hours. The cells were then incubated with the corresponding dimerized peptide coacervates at different concentrations for 2 h each. The cells were then incubated with calcein-AM for live cell staining and PI for dead cell staining for 10 minutes. Fluorescence images of the cells were captured using a Leica TCS 264 SP5X system.

Internalization of Nile red-loaded peptide coacervates: HeLa cells were cultured in DMEM medium containing 10% FBS, 1% penicillin/streptomycin (complete DMEM) in 5% CO_2 at 37°C. Cells were seeded in an 8-well µ-slide for 24 hours, and the medium was changed. The cells were then incubated with 10 µL Nile Red-loaded peptide coacervates (Nile Red: 0.01 mg mL^{-1}, peptide: 5 mg mL^{-1}) for 2 h. The cells were then washed and stained with Lysol tracker green and cell mask deep red for 10 min. The cells were washed 3 times with PBS. Fluorescence images of the cells were then captured using a Leica TCS 264 SP5X system.

Study of Internalization Mechanism. Various inhibitors were used to study the internalization pathway of coacervates. HeLa cells were cultured in DMEM medium containing 10% FBS, 1% penicillin/streptomycin (complete DMEM) in 5% CO2 at 37°C. Cells were seeded in an 8-well µ-slide for 24 hours, and the medium was changed. Then HeLa cells were then treated separately with chlorpromazine (CPM, 30 µM), amiloride chloride (AM, 20 µM), sodium azide (NaN3, 100 mM), or methyl-β-cyclodextrin (MβCD, 2.5 mM) for 1 h, followed by the addition of

10 μL of dipeptide coacervates (Nile Red: 0.01 mg mL-1, peptide: 5 mg mL-1) was added. After another 4 h of incubation, the cells were washed twice with PBS. The cells were then stained with Cell Mask Deep Red for 10 min. Fluorescence images of the cells were then captured using a Leica TCS 264 SP5X system. For the 4 °C treated group, the HeLa cells were pre-incubated for 1 h and kept at low temperature during the 4 h uptake process. Cells treated with dipeptide coacervates without any inhibitors (blank) were also examined.

Bio-orthogonal catalytic performance of Ru-loaded peptide coacervates with living cells: The bio-orthogonal catalytic performance with cells was investigated using confocal imaging and microplate reader assay.

(i) Confocal imaging measurements: HeLa cells were cultured in DMEM medium containing 10% FBS, 1% penicillin/streptomycin (complete DMEM) in 5% CO_2 at 37°C. Cells were seeded in an 8-well μ-slide for 24 hours, and the medium was changed. The cells were then incubated with 10 μL Ru-loaded peptide coacervates (Ru: 0.01 mg mL^{-1}, peptide: 5 mg mL^{-1}) for 2 h. The cells were then washed three times with PBS. The cells were then refreshed with 200 μL culture medium containing caged rhodamine 110 (0.01 mg mL^{-1}) and incubated for 18 h. The cells were then washed with PBS and stained with cell mask deep red for 10 min. Finally, the cells were washed with PBS and the fluorescence images of the cells were captured using a Leica TCS 264 SP5X system.

(ii) Microplate reader measurements: HeLa cells were cultured in DMEM medium containing 10% FBS, 1% penicillin/streptomycin (complete DMEM) in 5% CO2 at 37°C. The cells were seeded in a 96-well for 24 hours and then the medium was changed. The cells were then incubated with 10 μL Ru-loaded peptide coacervates (Ru: 0.01 mg mL-1, peptide: 5 mg mL^{-1}) for 2 h. The cells were then washed three times with PBS. The cells were then refreshed with 200 μL culture medium containing caged Rhodamine 110 (0.01 mg mL^{-1}) and incubated for 18 h. The cells were then washed three times with PBS. Finally, the fluorescence intensity of the uncaged product was measured using a microplate reader assay.

2. Supplementary Figures and Tables

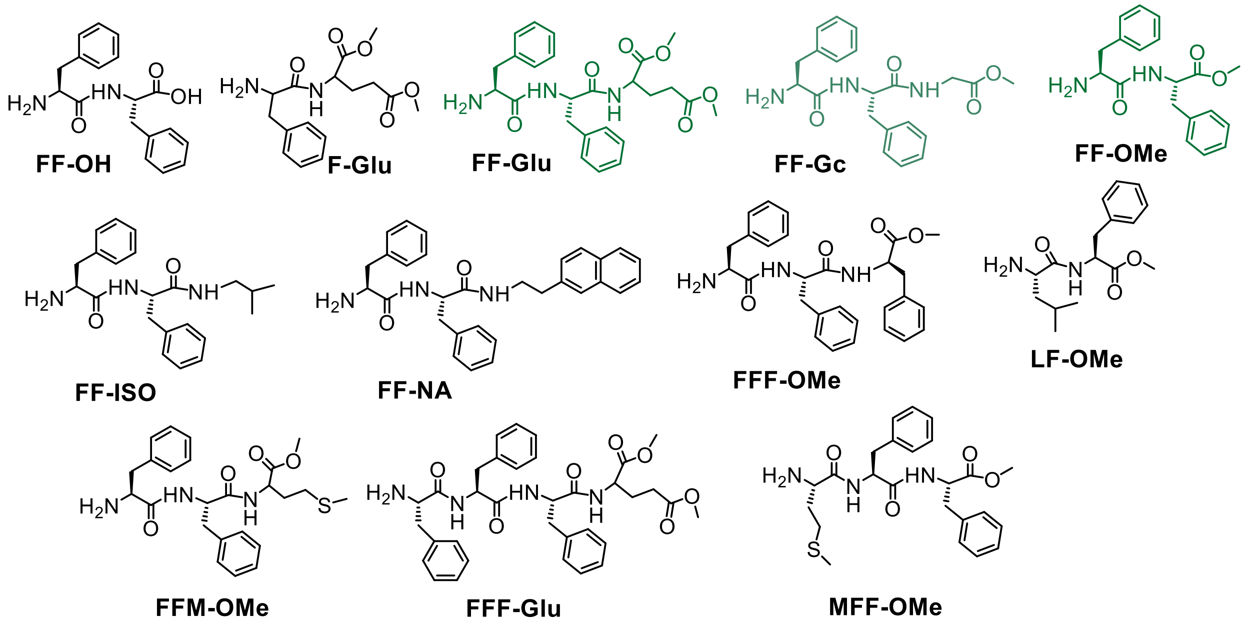

Supplementary Figure 5. Chemical structures of the diphenylalanine precursor (FF-OH) and derivatives (FF-X). Compounds marked in green were able to undergo pH-induced phase separation to form peptide coacervates. The other compounds show different phase separation behaviors: e.g., formation of gel-like, fiber-like and solid aggregates.

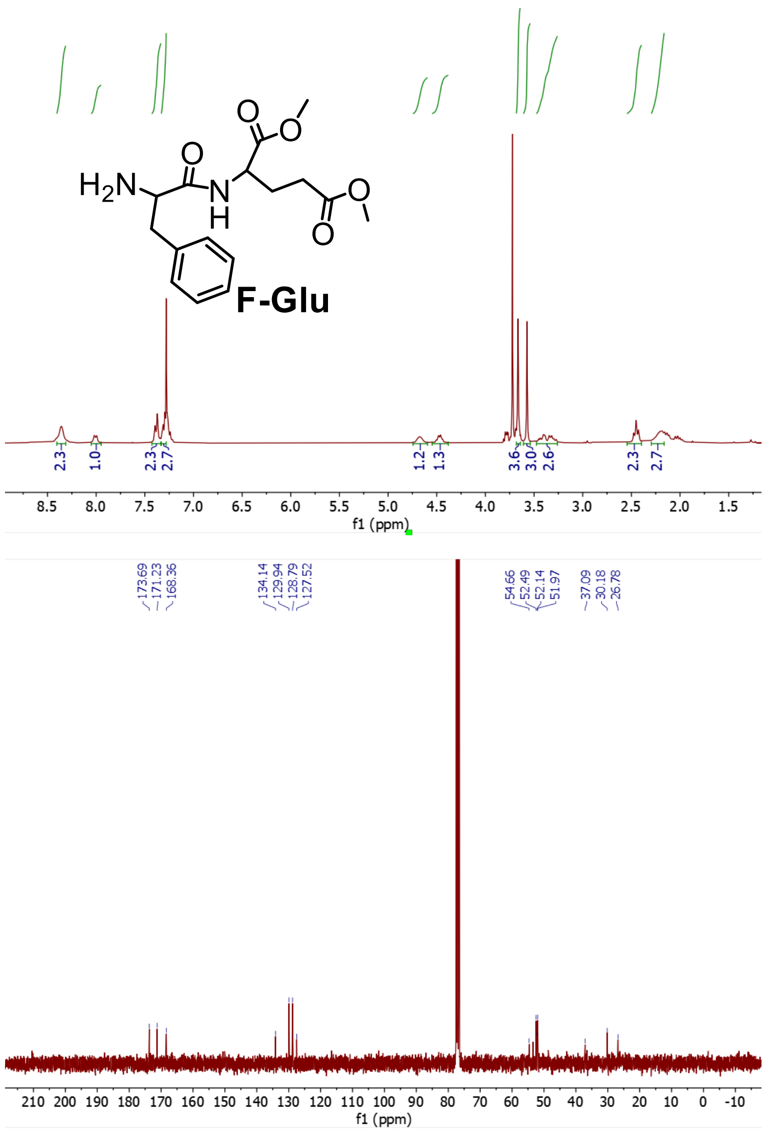

Supplementary Figure 6. ^{1}H NMR and ^{13}C NMR of F-Glu.

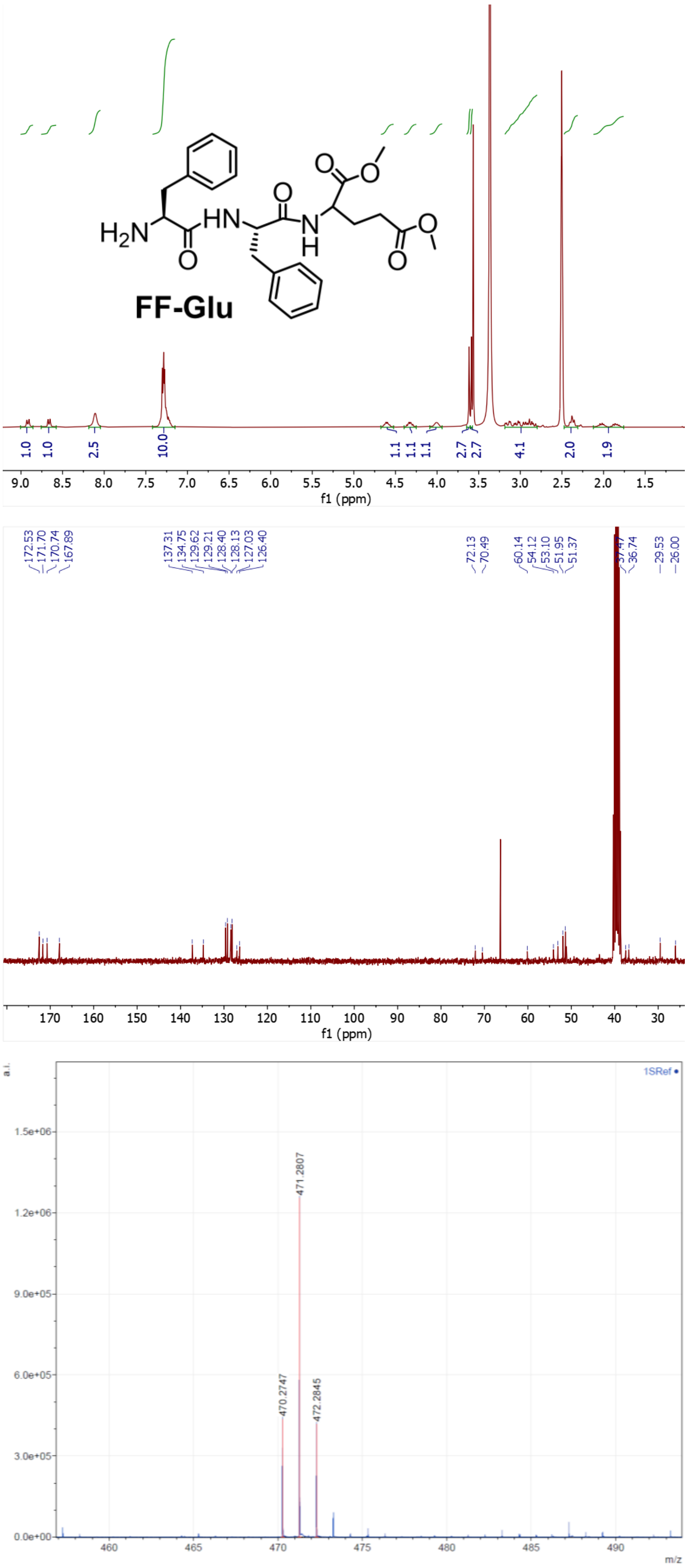

Supplementary Figure 7. ^{1}H NMR, ^{13}C NMR and MS of FF-Glu.

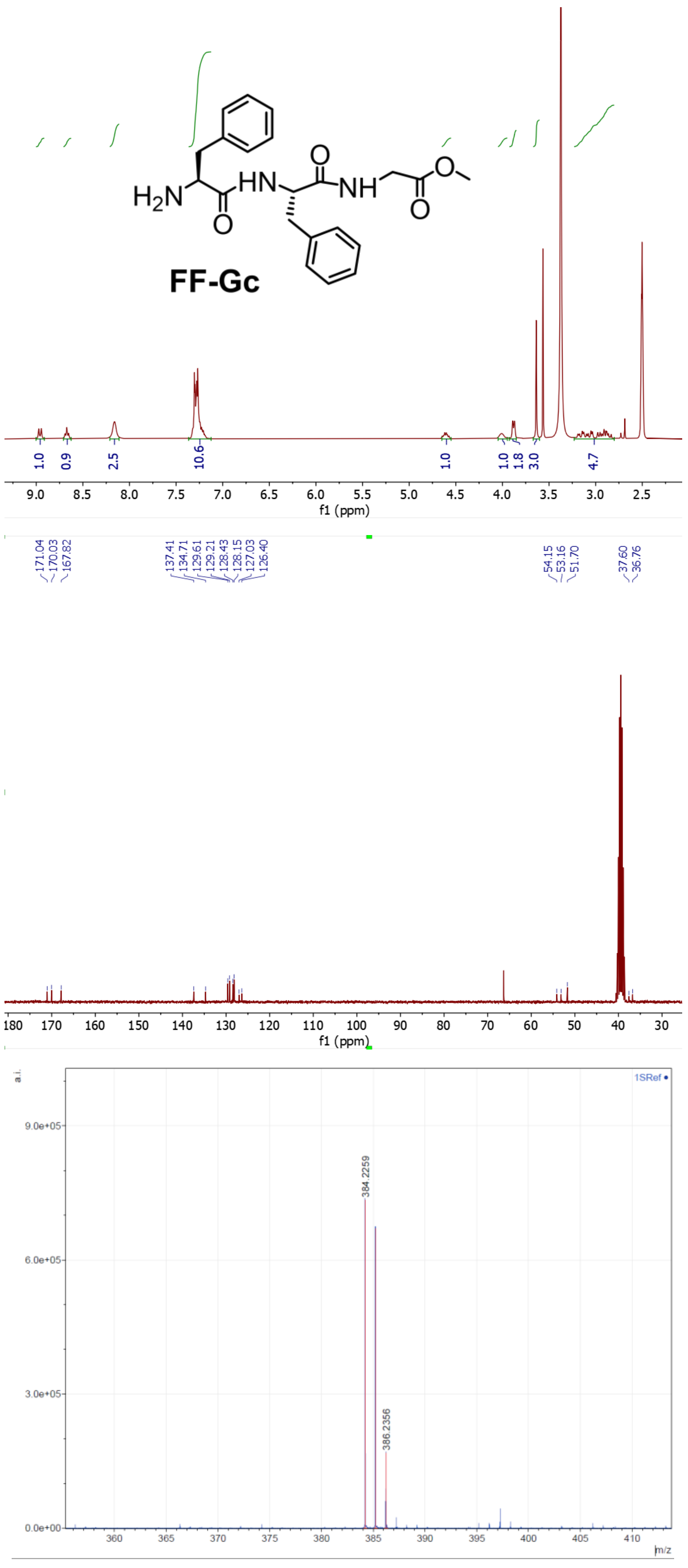

Supplementary Figure 8. ^{1}H NMR, ^{13}C NMR and MS of FF-Gc.

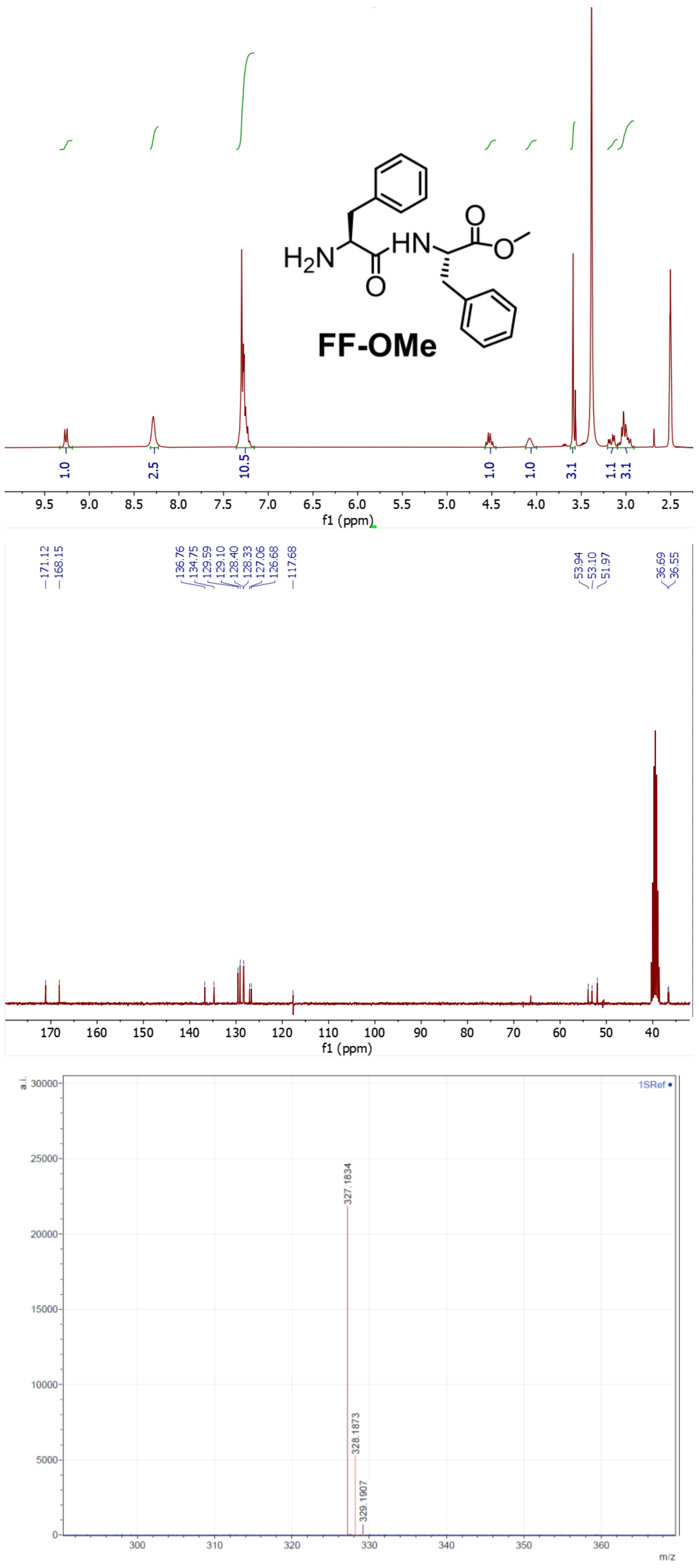

Supplementary Figure 9. ^{1}H NMR, ^{13}C NMR and MS of FF-OMe.

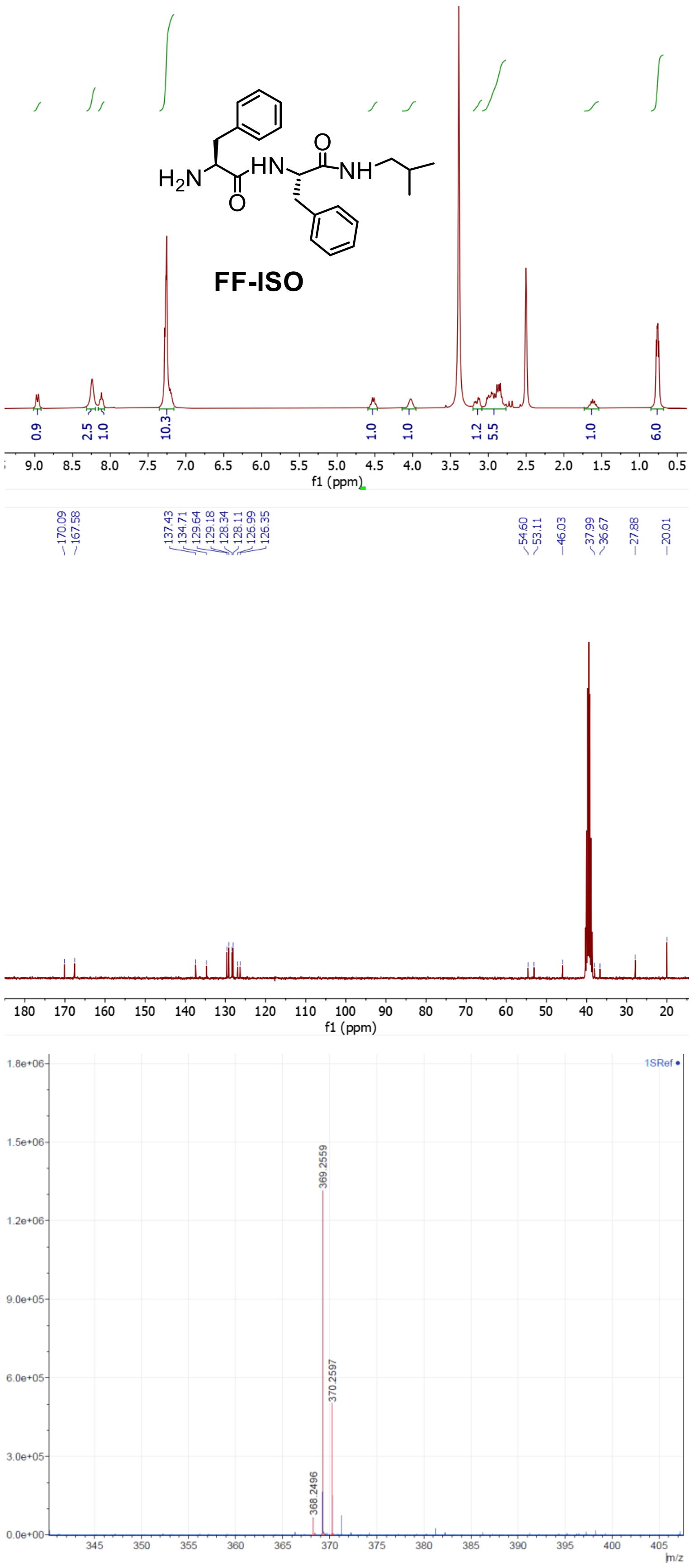

Supplementary Figure 10. ^{1}H NMR, ^{13}C NMR and MS of FF-ISO

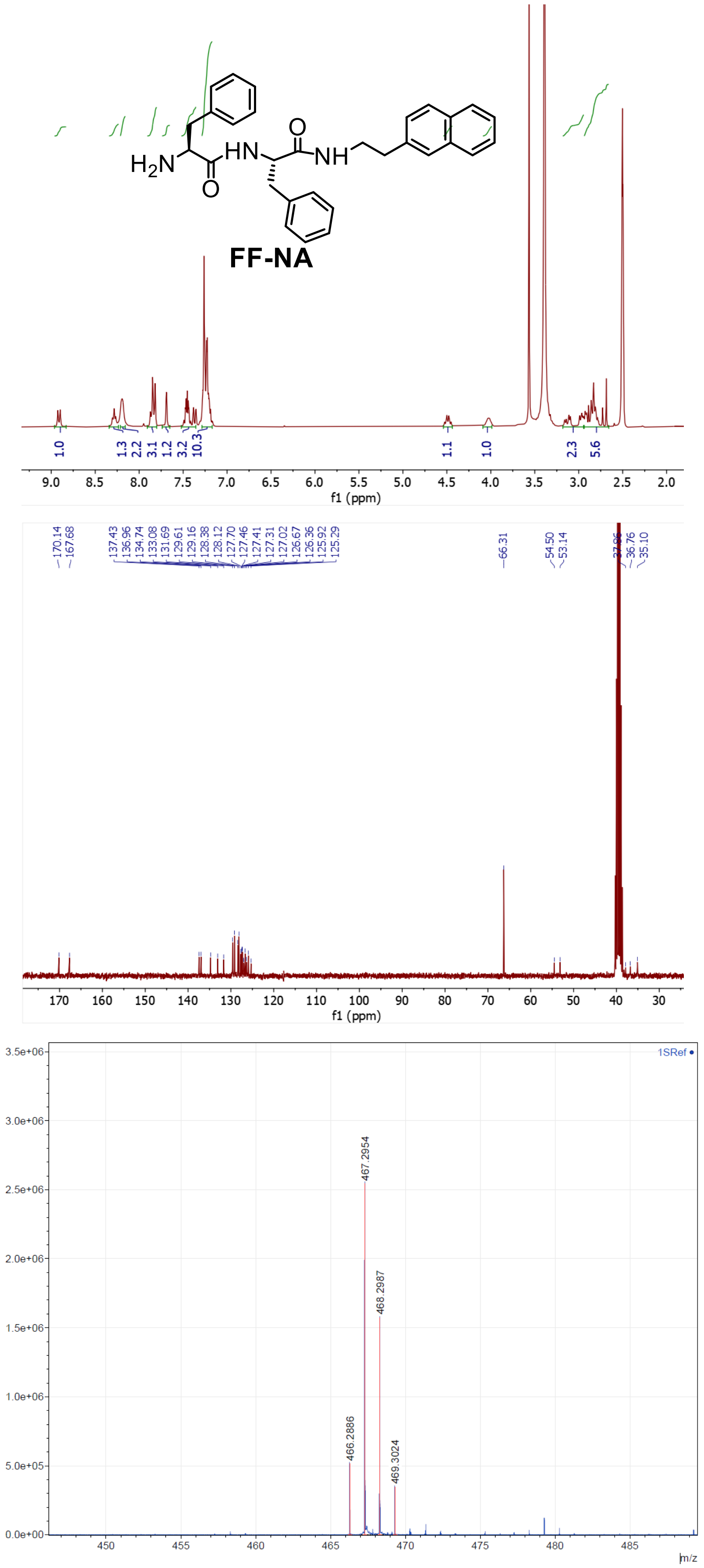

Supplementary Figure 11. ^{1}H NMR, ^{13}C NMR and MS of FF-NA

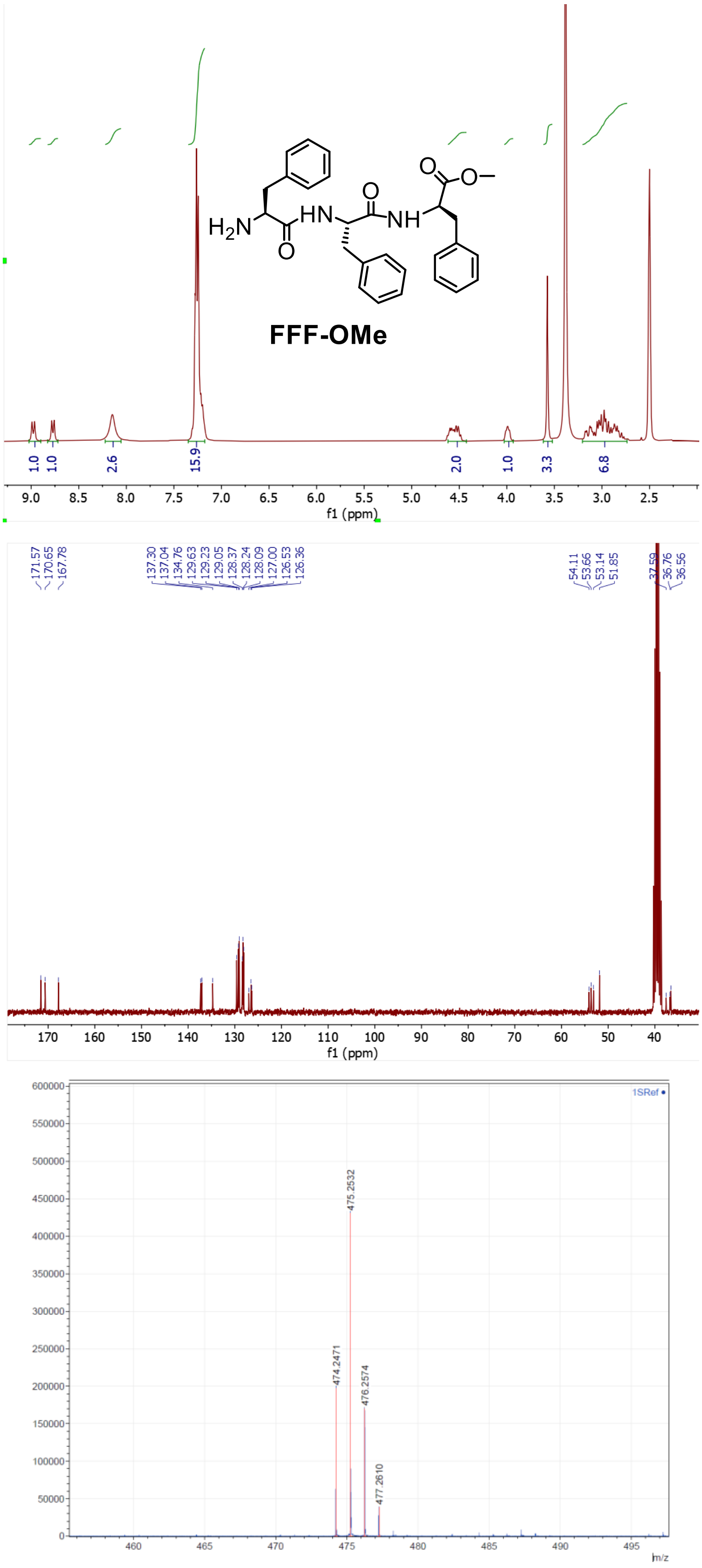

Supplementary Figure 12. ^{1}H NMR, ^{13}C NMR and MS of FFF-OMe

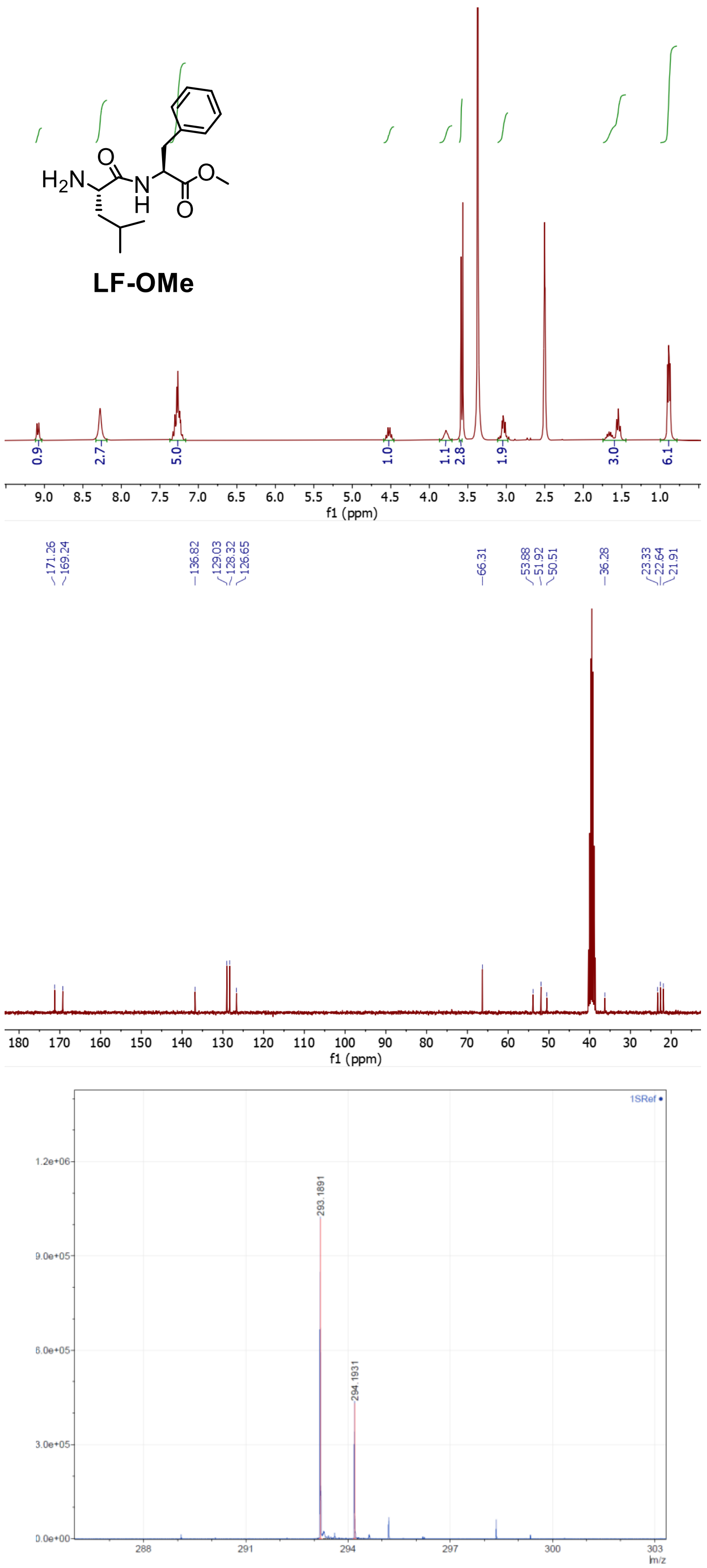

Supplementary Figure 13. ^{1}H NMR, ^{13}C NMR and MS of LF-OMe

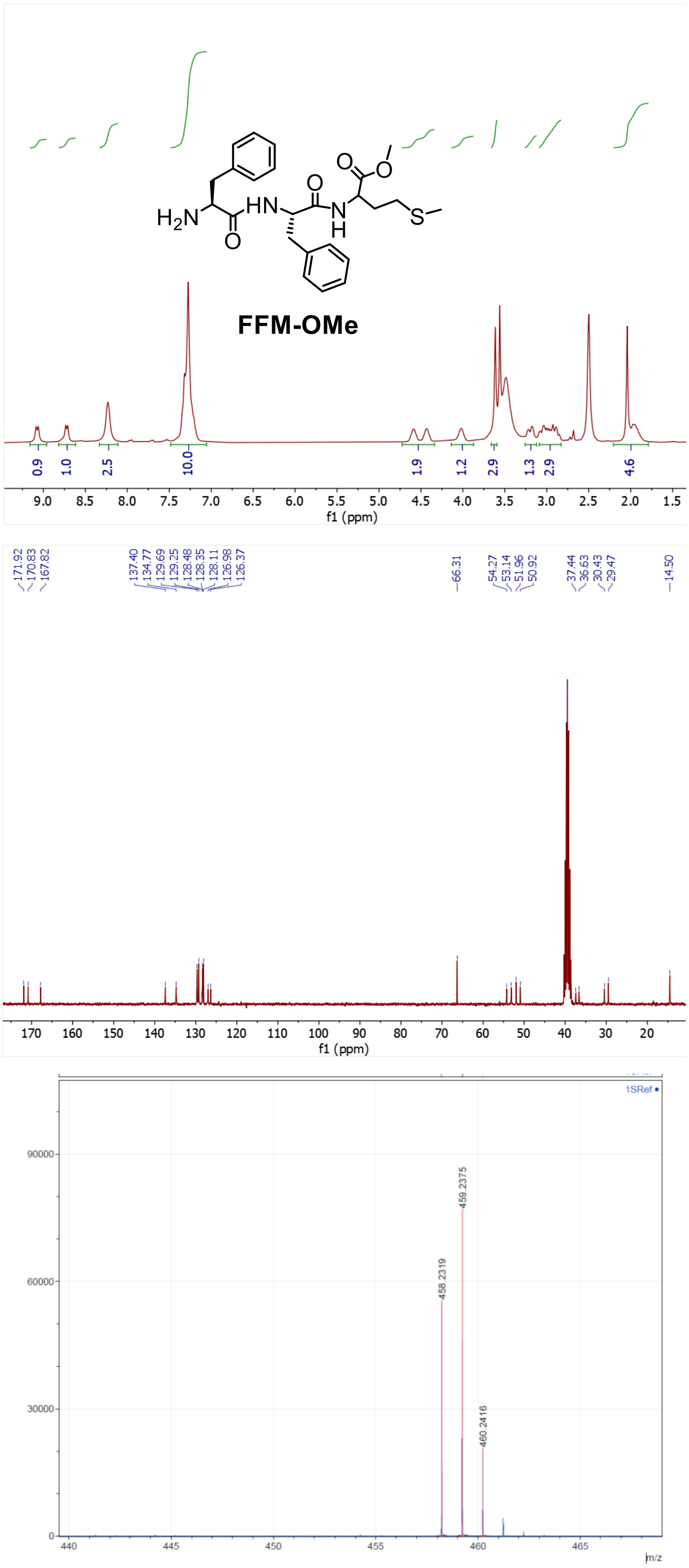

Supplementary Figure 14. ^{1}H NMR, ^{13}C NMR and MS of FFM-OMe

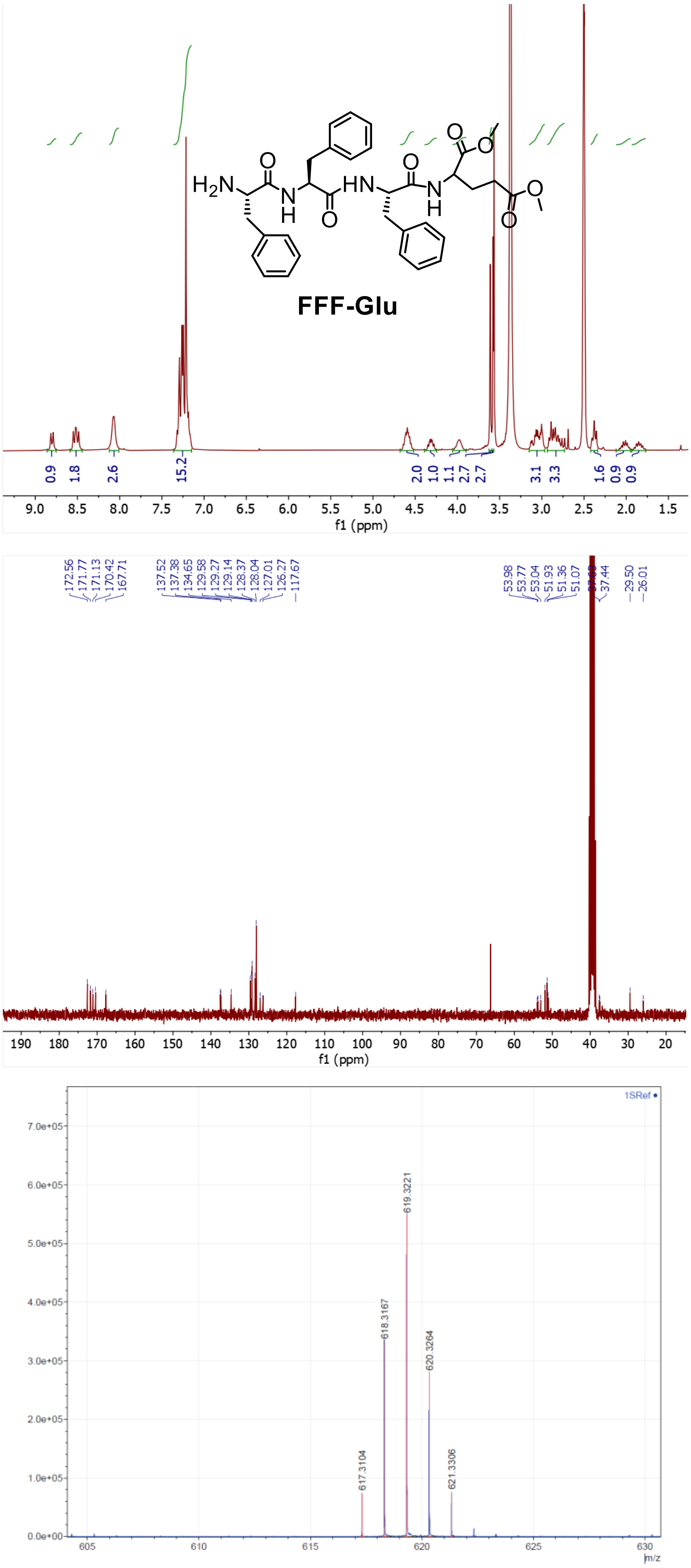

Supplementary Figure 15. ^{1}H NMR, ^{13}C NMR and MS of FFF-Glu

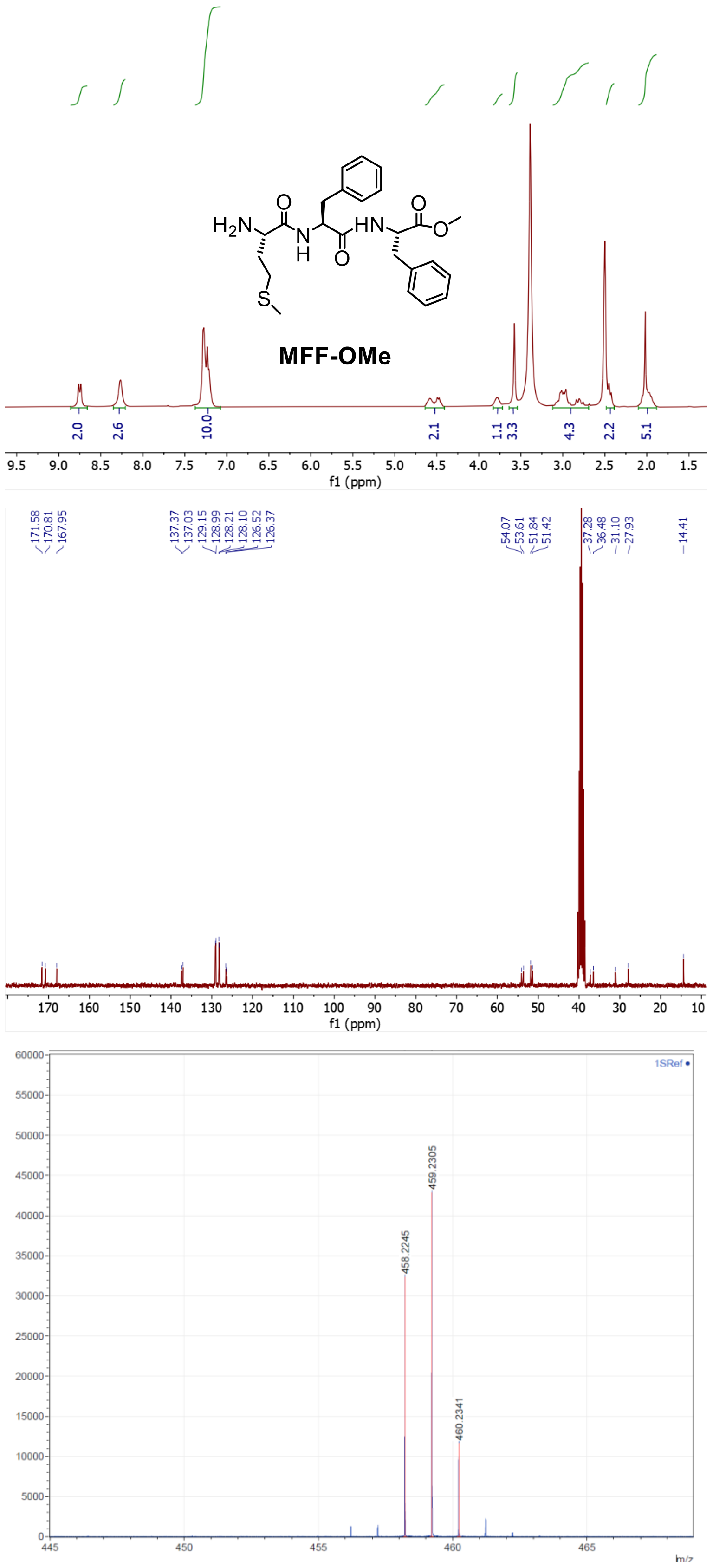

Supplementary Figure 16. ^{1}H NMR, ^{13}C NMR and MS of MFF-OMe

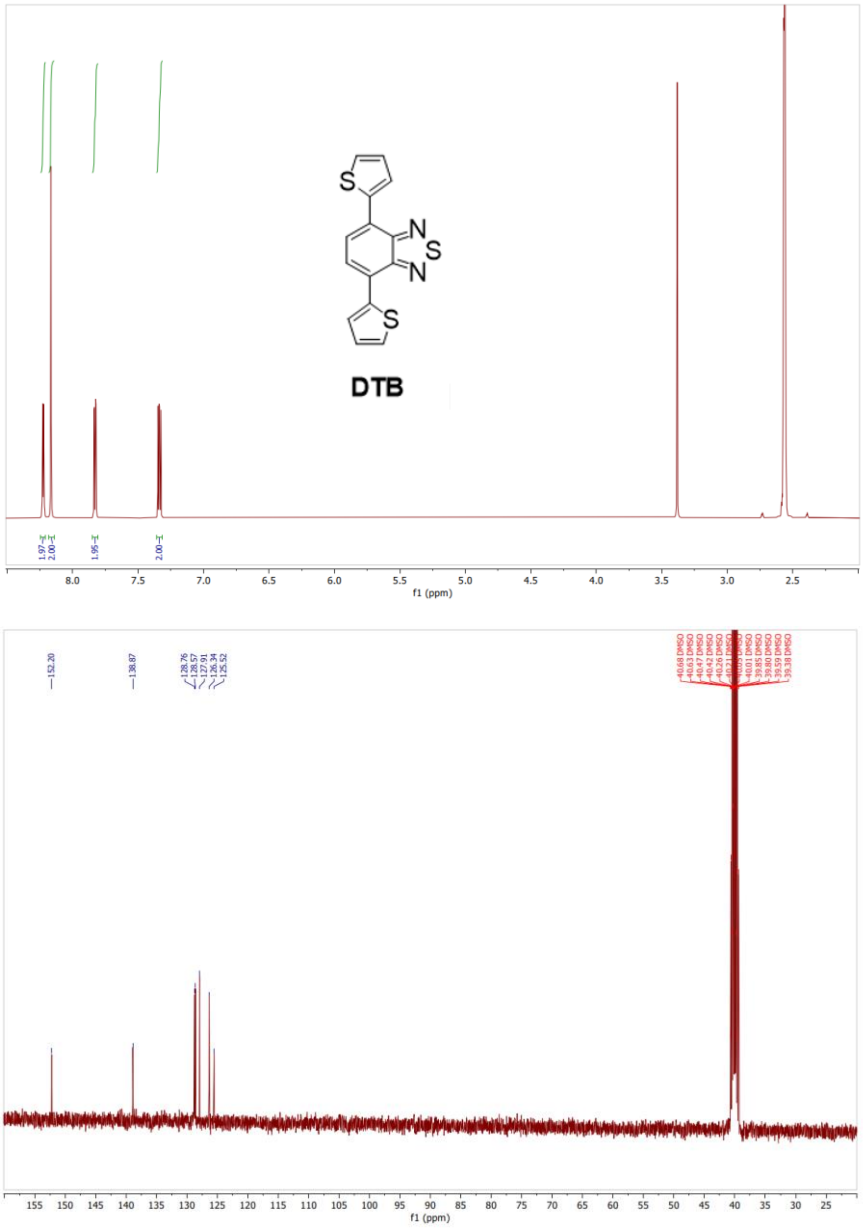

Supplementary Figure 17. ^{1}H NMR and ^{13}C NMR of DTB

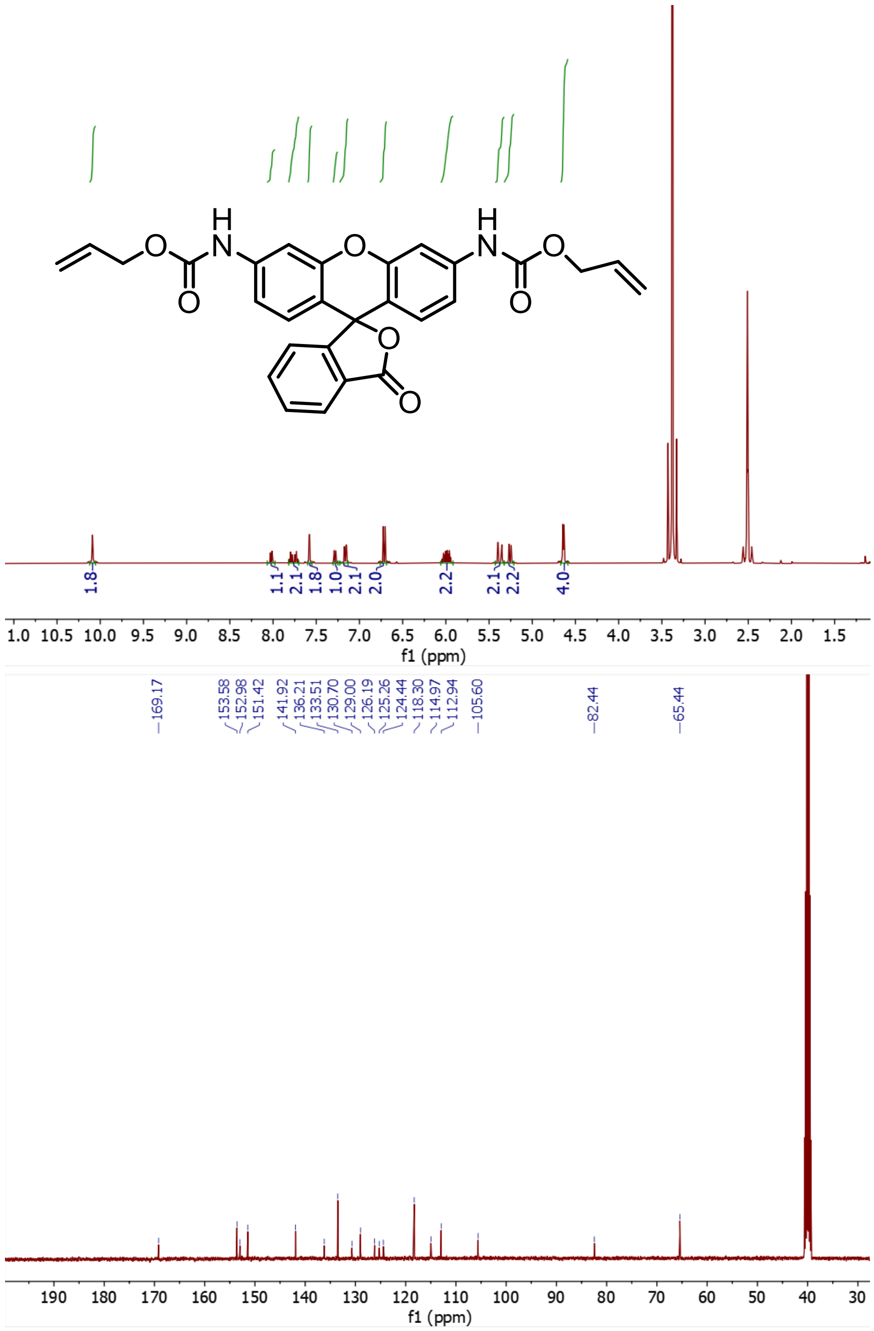

Supplementary Figure 18. ^{1}H NMR and ^{13}C NMR of caged Rho-110

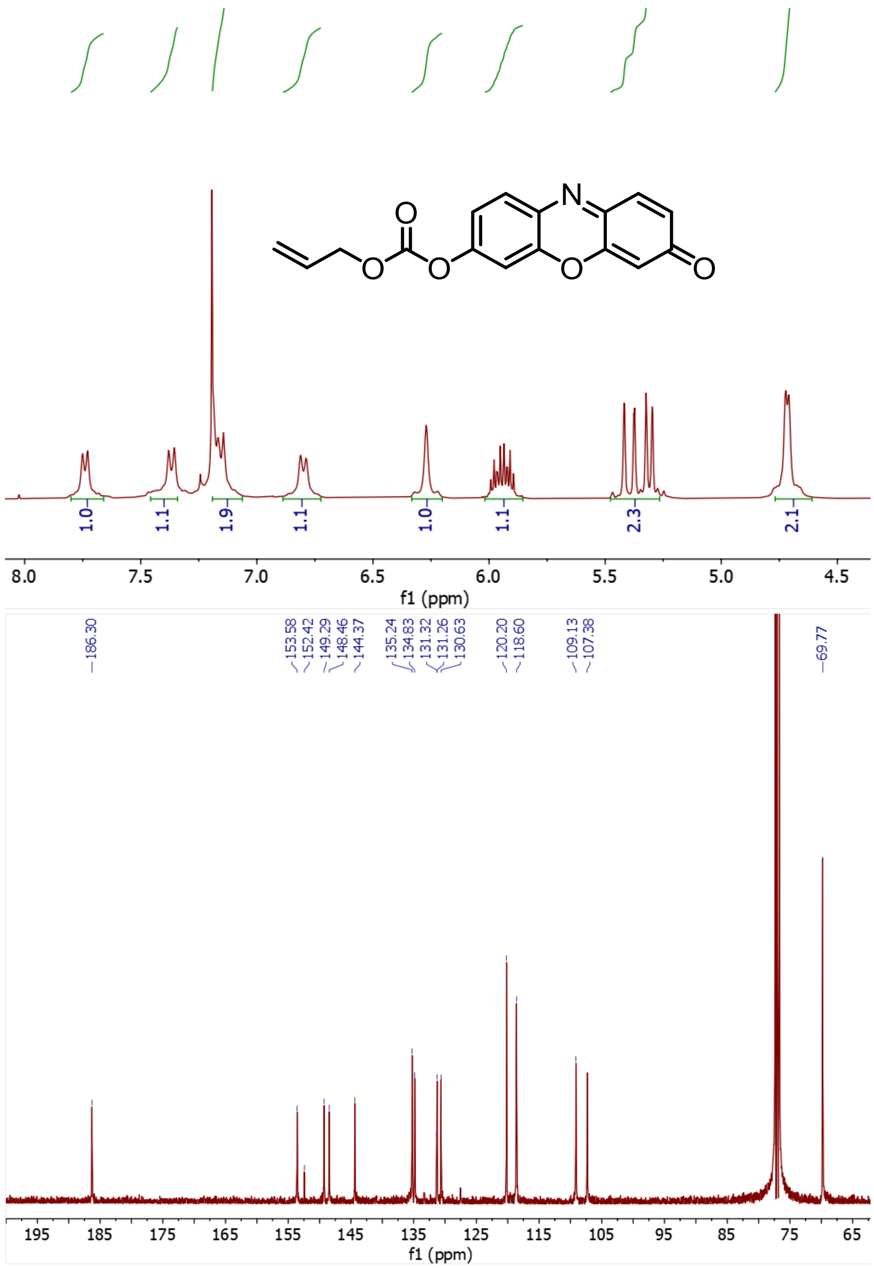

Supplementary Figure 19. ^{1}H NMR and ^{13}C NMR of caged resorufin

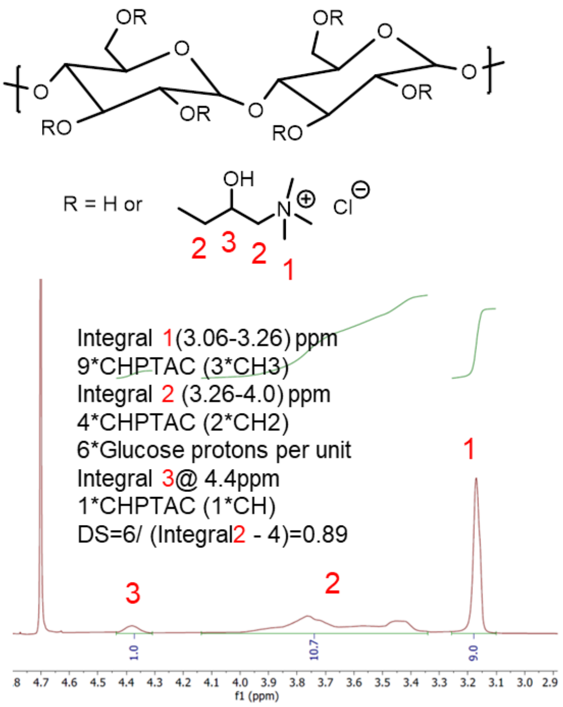

Supplementary Figure 20. ^{1}H NMR of charged amylose derivatives with the calculation of the degree of substitution (DS = number of modifying groups per glucose unit), of quaternized carboxymethylated amylose.

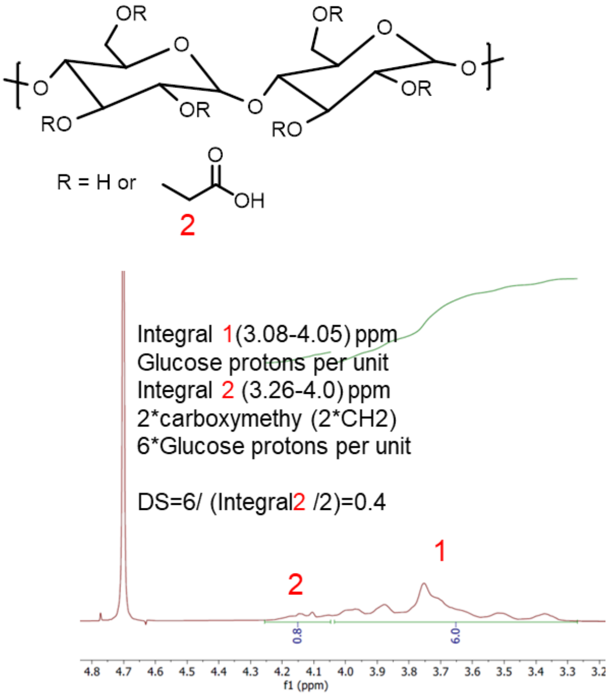

Supplementary Figure 21. ^{1}H NMR of charged amylose derivatives with the calculation of the degree of substitution (DS = number of modifying groups per glucose unit), of carboxymethylated amylose.

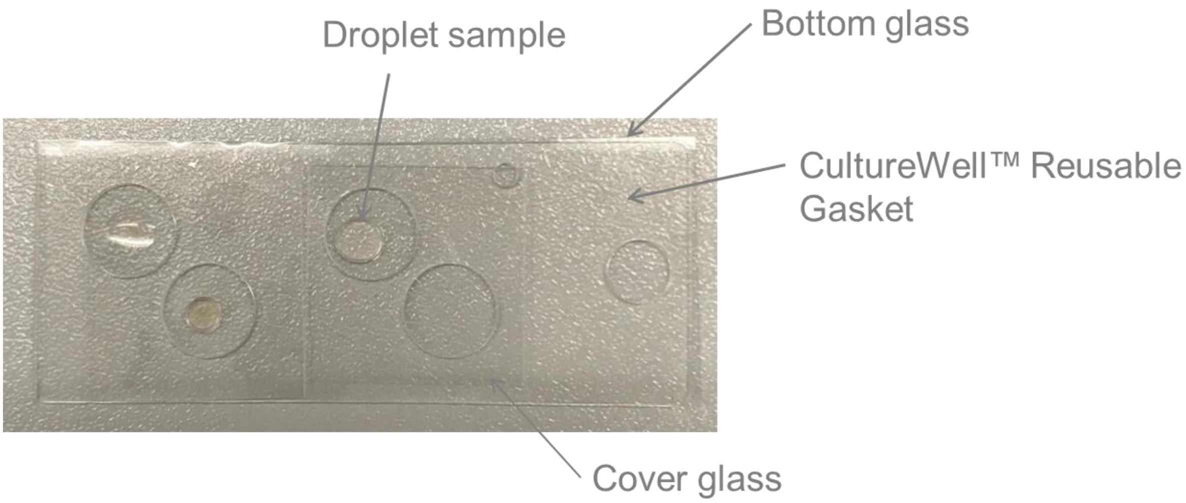

Supplementary Figure 22. Glass setup for droplet observation with microscopy imaging.

Supplementary Table 1.

Summary of the pH-induced phase separation of the diphenylalanine precursor and derivatives.

Peptide abbreviation	~pH 6	~pH 9
F-Glu	Solution	Solution
FF-Glu	Solution	Coacervates
FF-Glc	Solution	Coacervates
FF-OMe	Solution	Coacervates
FF-ISO	Solution	Gel-like
FF-NA	Aggregates	NA
FFF-OMe	Aggregates	NA
LF-OMe	Solution	Solution
FFM-OMe	Solution	Fiber-like
FFF-Glu	Aggregates	NA
MFF-OMe	Aggregates	NA

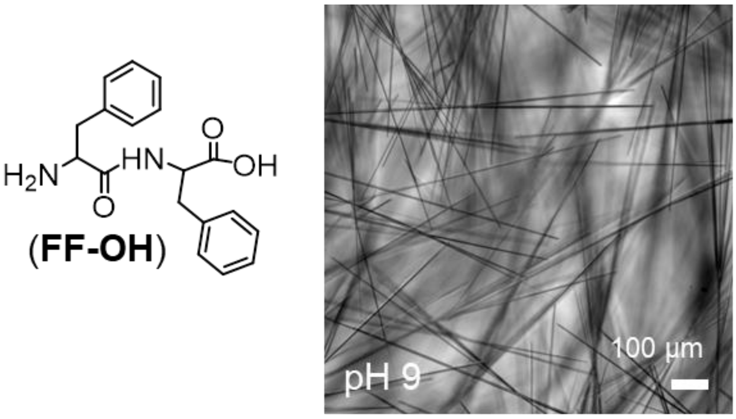

Supplementary Figure 23. Left: Chemical structure of the commercially available **FF-OH**; Right: Microscopic images of FF-OH at pH 9 buffer (in 5 mM HEPES) showed long fiber formation which is similar to observations in literature. Scale bar=100 µm in the microscopy image. Similar results were obtained with 3 samples measured independently.

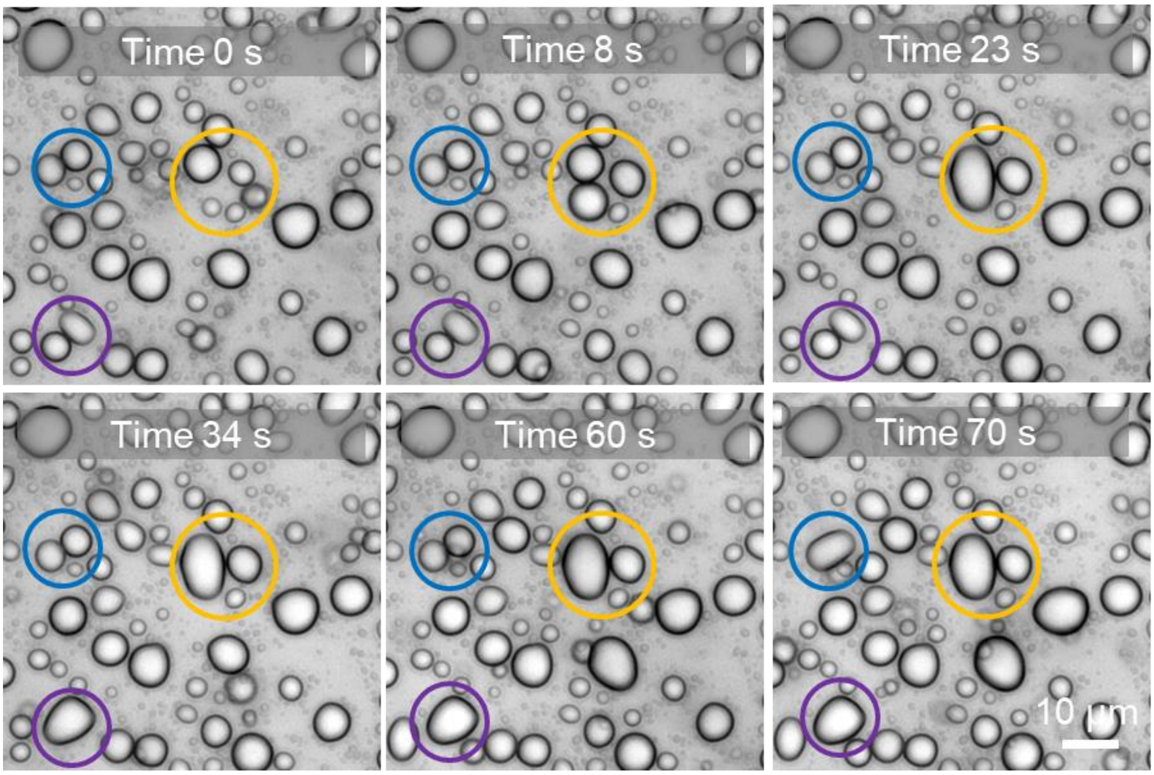

Supplementary Figure 24. Coacervates formed from FF-OMe dipeptide could coalesce to form larger droplets. Scale bar=10 μm in all microscopy images. Similar results were obtained with 3 samples measured independently.

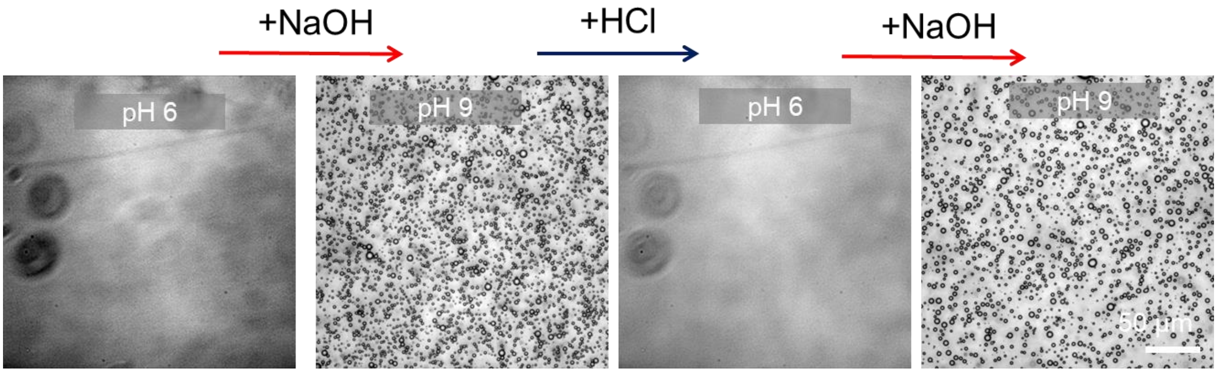

Supplementary Figure 25. Coacervates formed from FF-OMe peptide are reversible, undergoing phase separation at pH ~9 (in 5 mM HEPES), but soluble at pH ~6. Scale bar=50 μm in all microscopy images. Similar results were obtained with 3 samples measured independently.

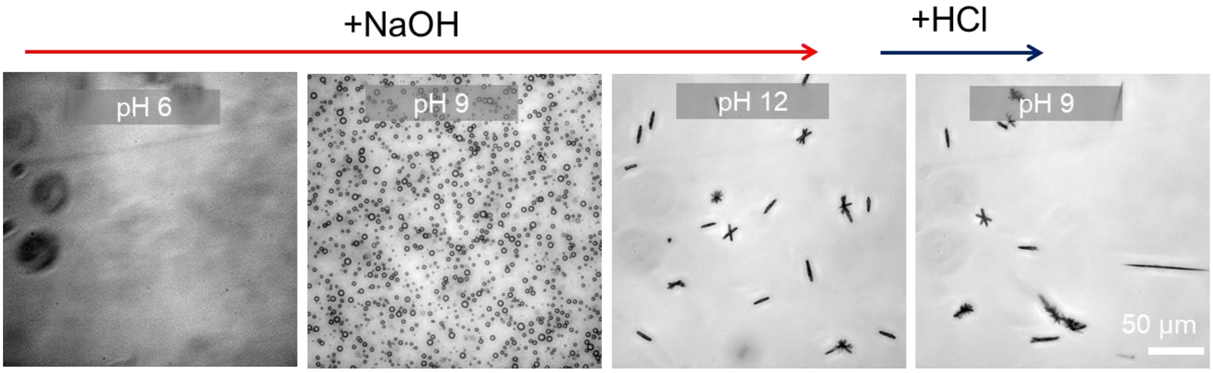

Supplementary Figure 26. The phase separation behaviour of FF-OMe peptide depends on the pH of the environment, which produces a liquid droplet at pH 9 (in 5 mM HEPES). The liquid droplet transforms into tubular aggregates when the pH reaches 12, which cannot return to the liquid state even when the pH decreases. Scale bar=50 μm in all microscopy images. Similar results were obtained with 3 samples measured independently.

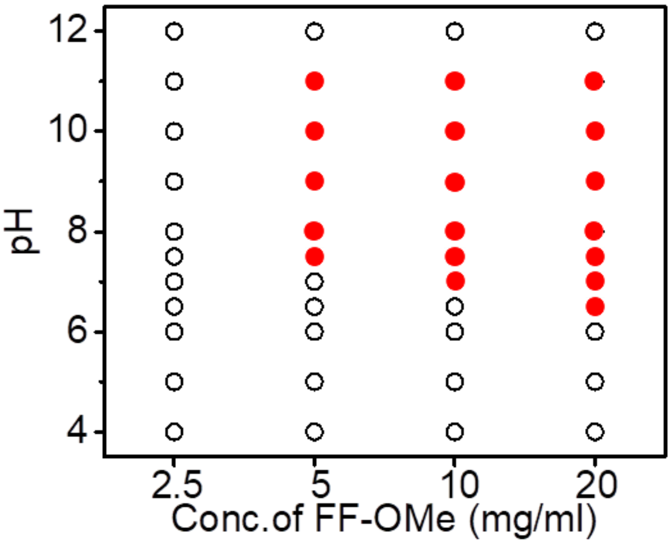

Supplementary Figure 27. Phase separation diagram of FF-OMe. The formation of dipeptide coacervate droplets (red, filled dots) depends on dipeptide concentration and pH.

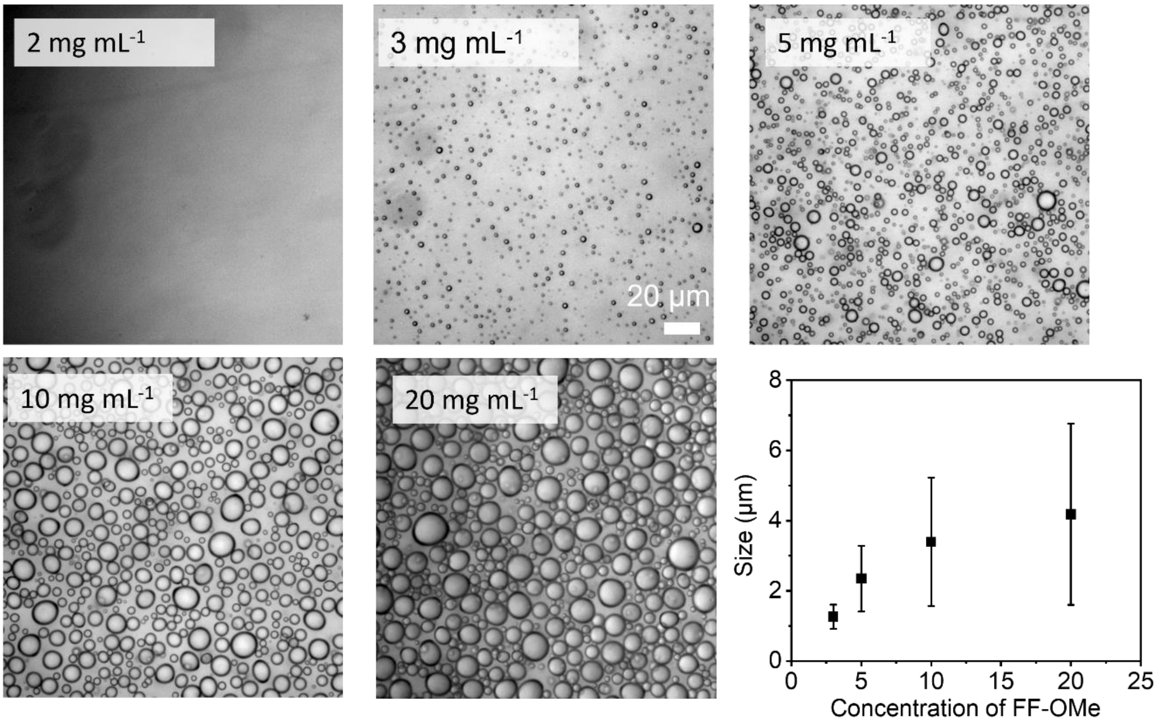

Supplementary Figure 28. Concentration dependent phase separation of FF-OMe at pH ~ 9. Based on the microscopy imaging, the threshold concentration for FF-OMe coacervate formation was approximately 3 mg mL^{-1}. The large error bars are consistent with the uncontrollable nature of the manual mixing process. The presence of large droplets at higher concentrations may also be a result of enhanced Ostwald ripening due to the increased number of droplets with a heterogeneous size distribution in the system. Scale bar=20 μm in

all microscopy images. Similar results were obtained with 3 samples measured independently. Data represent mean ± SD for n = 5 representative microscopy images.

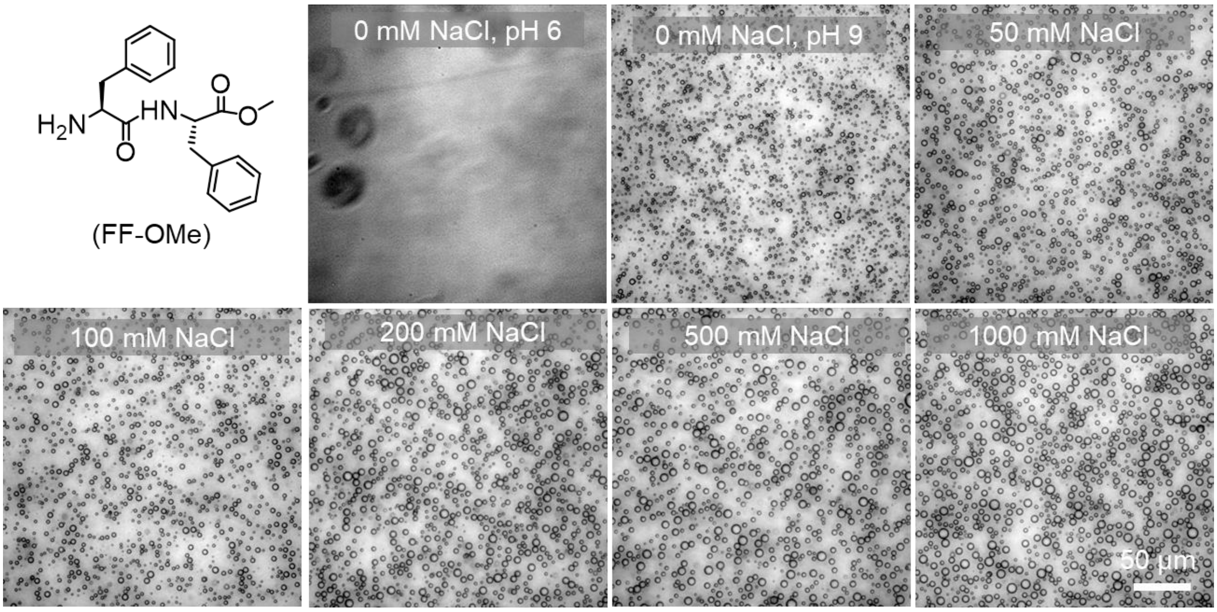

Supplementary Figure 29. Chemical structure of FF-OMe and its phase separation behavior in the presence of NaCl. Microscopy image analysis shows that the presence of NaCl contributes to the formation of droplets that do not easily undergo coalescence and surface wetting. Scale bar=50 μm in all microscopy images. Similar results were obtained with 3 samples measured independently.

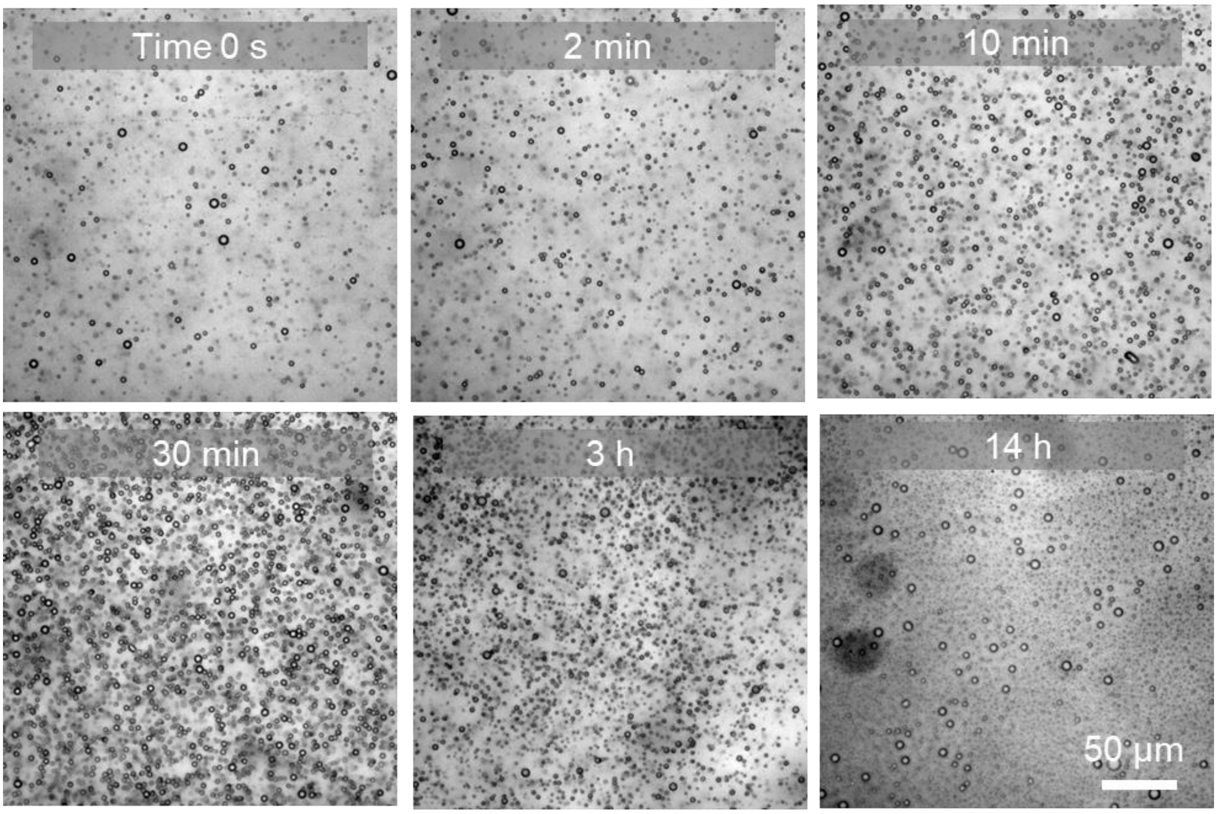

Supplementary Figure 30. Stability of FF-OMe coacervates (5 mg mL^{-1}) by incubation for different periods up to 14 h. A slight change in the size of the coacervates can be seen with increasing incubation time. For example, 50 droplets were measured at 0.5 h, 3 h and 14 h incubation. The size of the coacervates after 0.5 h, 3 h, and 14 h incubation was 1.6±0.3 µm, 1.5±0.5 µm, and 1.2±0.4 µm, respectively, showing a slight decrease in average size with increasing incubation time. Scale bar=50 µm in all microscopy images. Similar results were obtained with 3 samples measured independently.

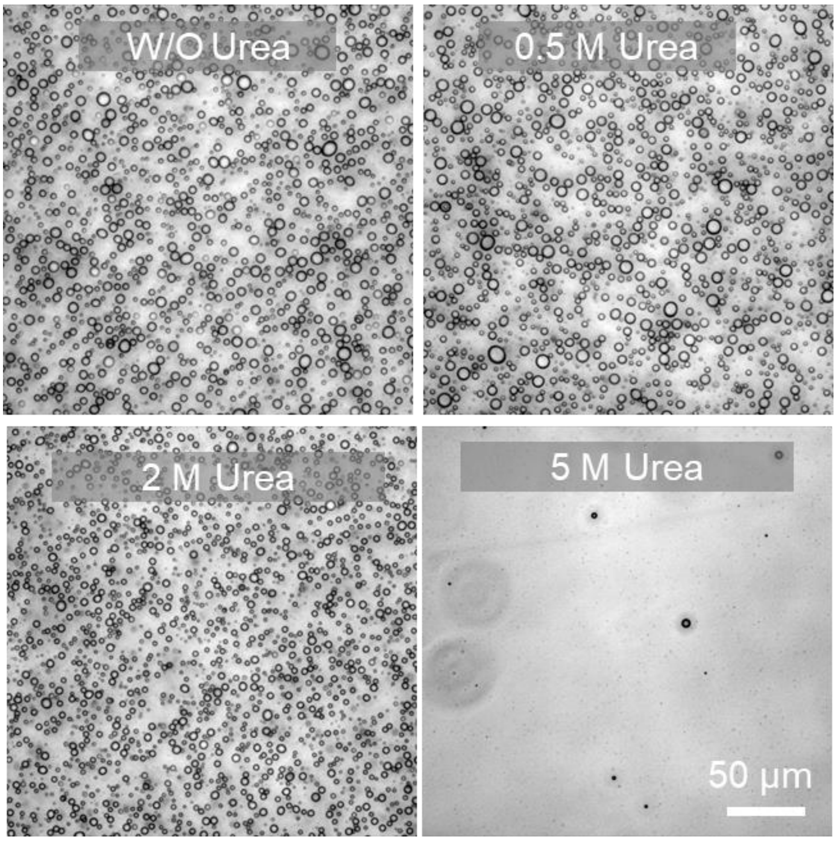

Supplementary Figure 31. The phase separation behaviour of FF-OMe peptide was affected by the presence of high concentrations of urea. The FF-OMe coacervates (HEPES/PBS, pH 9) were able to maintain their structure up to 2 M urea. The presence of 5 M urea induced the solvation of the coacervates. Scale bar=50 μm in all microscopy images. Similar results were obtained with 3 samples measured independently.

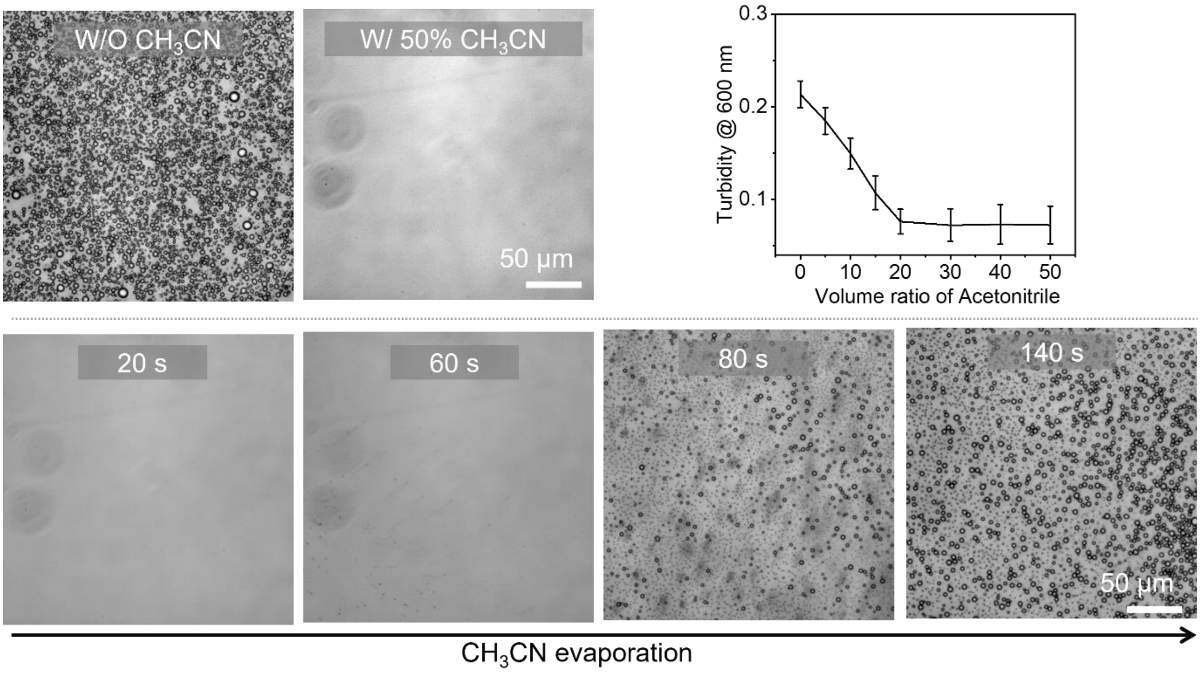

Supplementary Figure 32. Top: The phase separation behaviour of the FF-OMe peptide as a function of the organic solution. The coacervates (in 5 mM HEPES) were dissolved by adding 50% volume of CH_3CN to the solution, data represent mean ± SD for n = 3 independent samples. Bottom: After evaporation of CH_3CN, the FF-OMe is then able to phase separate and produce liquid coacervates again. Scale bar=50 μm in all microscopy images. Similar results were obtained with 3 samples measured independently.

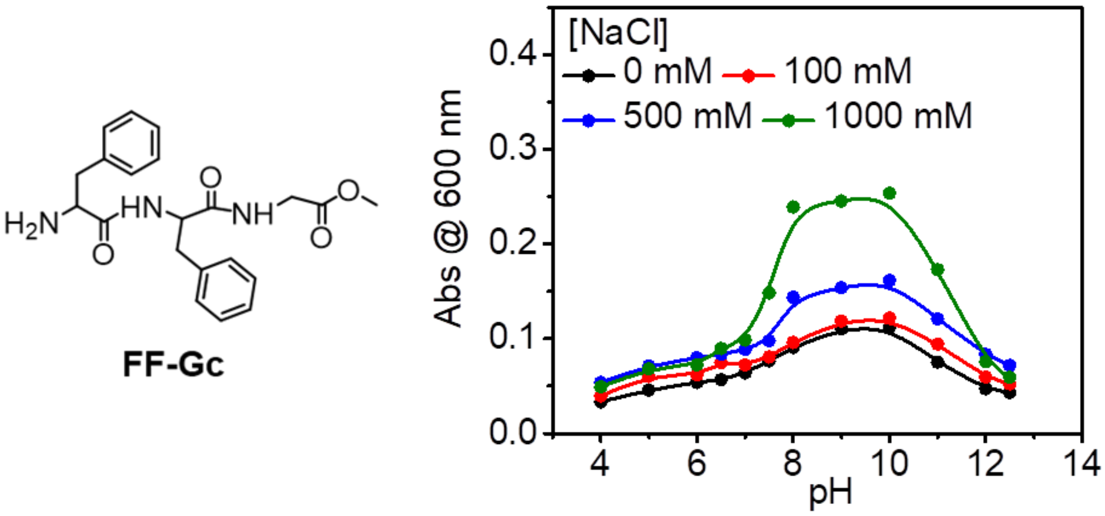

Supplementary Figure 33. Chemical structure of FF-Gc and its phase separation behavior as a function of pH and NaCl.

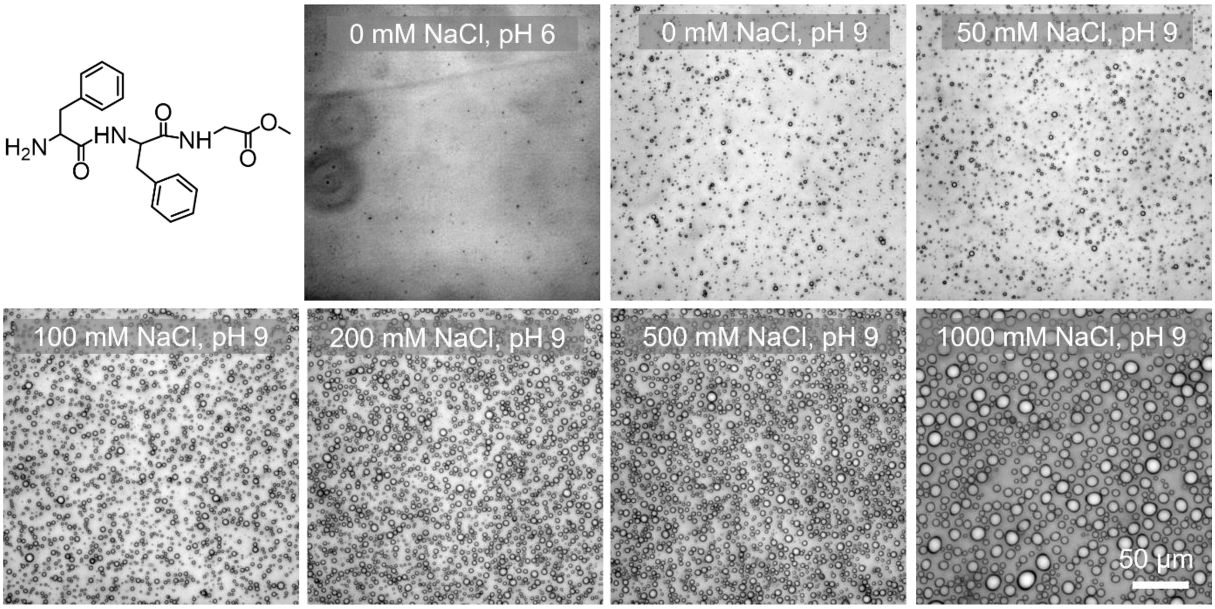

Supplementary Figure 34. Chemical structure of FF-Gc and the effect of NaCl on the phase separation behaviour of FF-Gc. Microscopic image analysis showed that the presence of NaCl contributed to a more pronounced phase separation behaviour. Scale bar=50 μm in all microscopy images. Similar results were obtained with 3 samples measured independently.

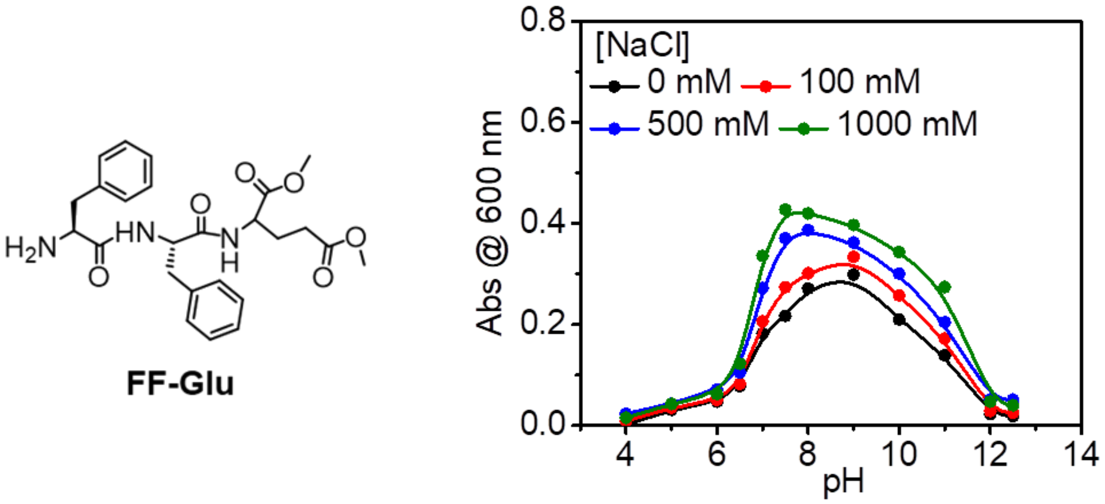

Supplementary Figure 35. Chemical structure of FF-Glu and its phase separation behavior as a function of pH and NaCl.

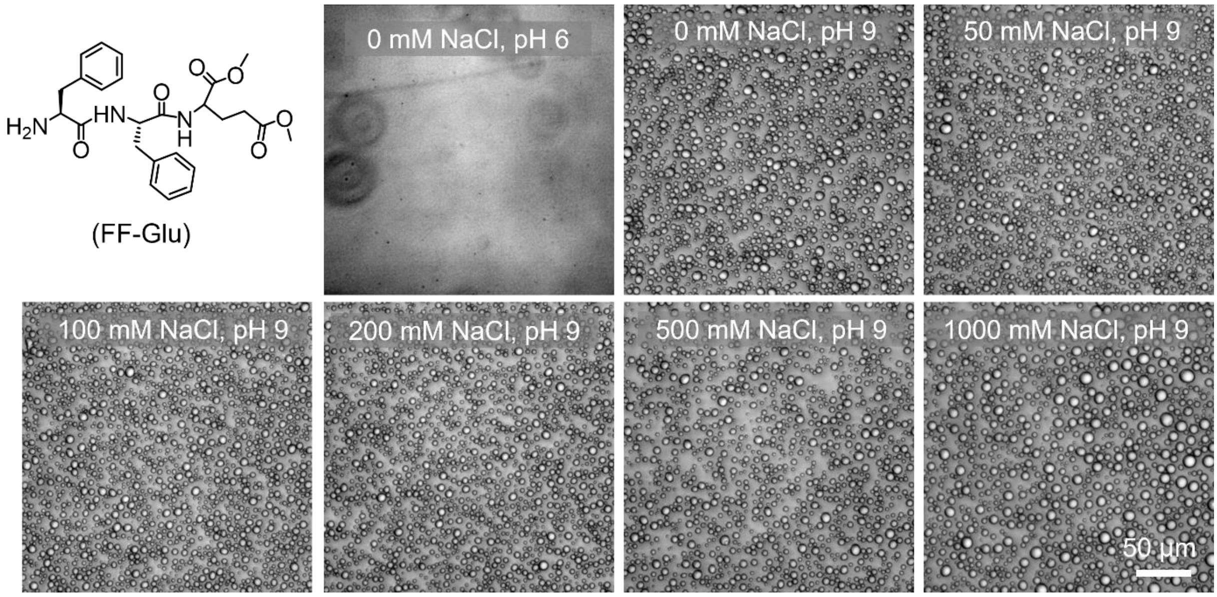

Supplementary Figure 36. Chemical structure of FF-Glu and the effect of NaCl on the phase separation behavior of FF-Glu. Microscopic image analysis showed that the presence of NaCl had no obvious effect compared to the results with FF-Gc. Scale bar=50 μm in all microscopy images. Similar results were obtained with 3 samples measured independently.

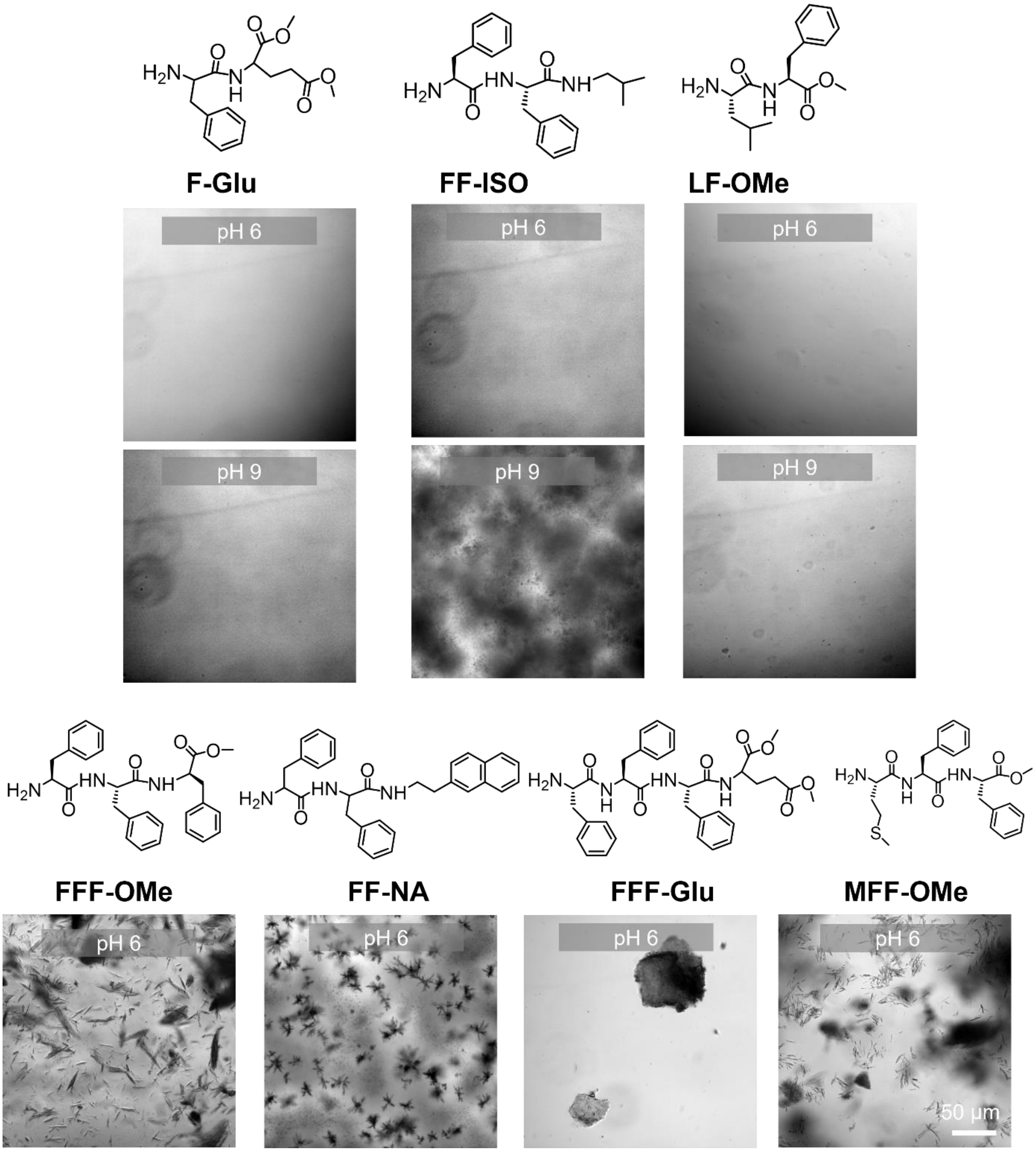

Supplementary Figure 37. Phase separation behaviour of various dipeptide derivatives. Top: the peptide compounds including F-Glu, FF-ISO and LF-OMe were solubilized in water at pH ~6 and remained so or as simple aggregates at pH 9. Bottom: the dipeptides including FFM-OMe, FF-NA, FFM-Glu and MFF-OMe were not solubilized at pH 6, they formed fiber-like aggregates or simple aggregates. Scale bar=50 μm in all microscopy images. Similar results were obtained with 3 samples measured independently.

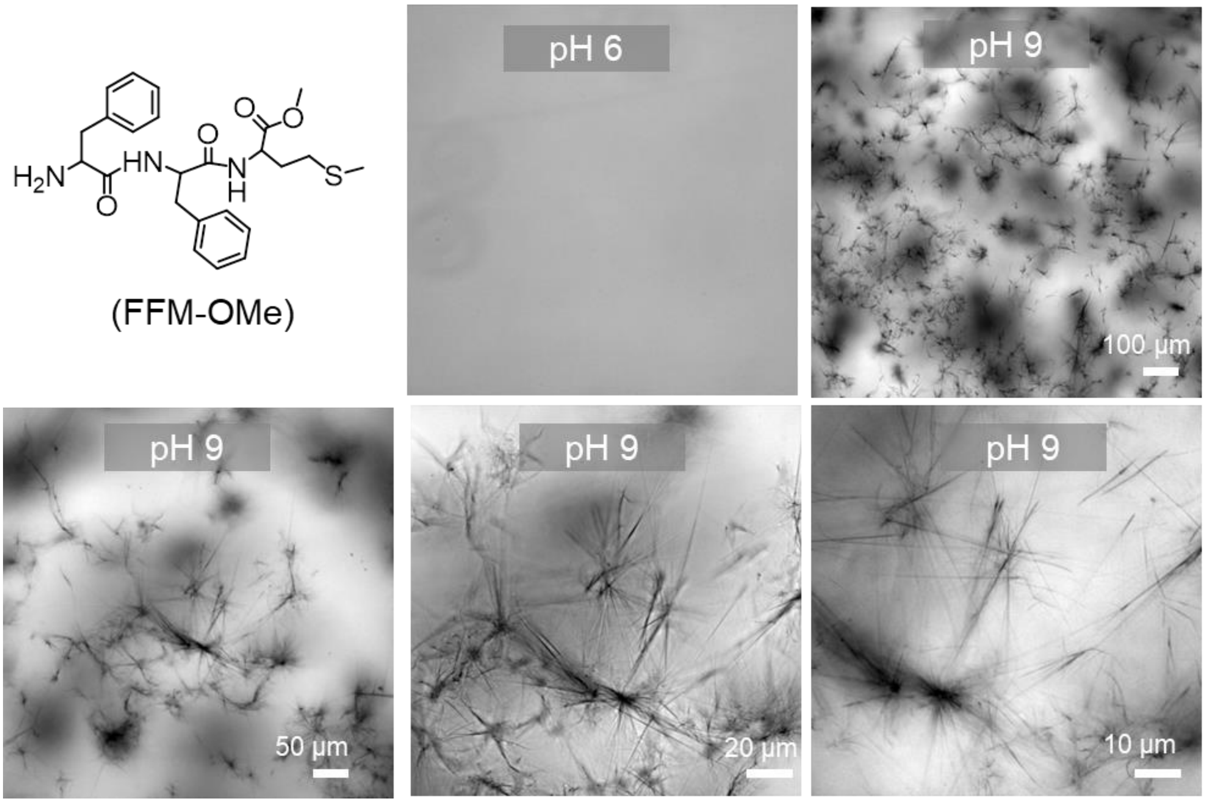

Supplementary Figure 38. Chemical structure of FFM-OMe and its pH-induced phase separation behavior. At pH 6, FFM-OMe is solubilized in 5 mM HEPES buffer, while at pH ~9, phase separation into a fiber-like material was observed. Similar results were obtained with 3 samples measured independently.

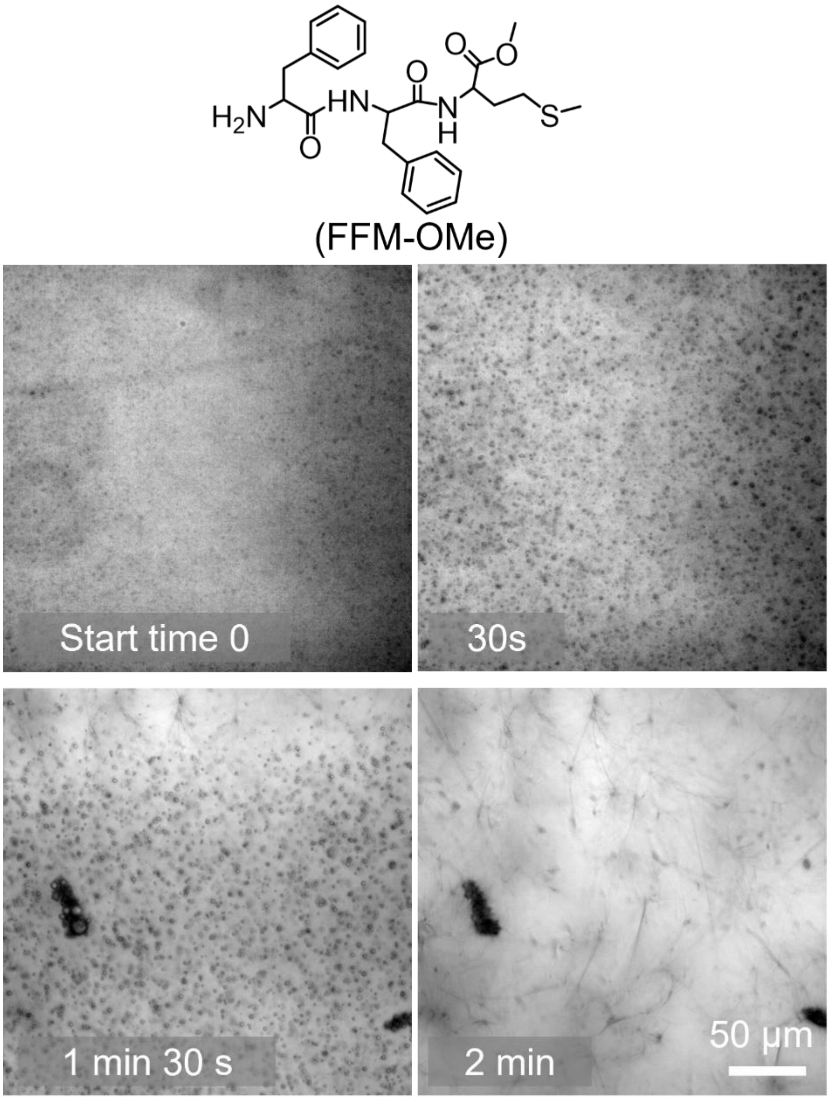

Supplementary Figure 39. Chemical structure of FFM-OMe and its time-dependent phase separation behaviour. Microscopic imaging shows that this compound initially undergoes liquid-liquid phase separation into droplets, which very quickly transform into a solid fiber-like structure. Scale bar=50 μm in all microscopy images. Similar results were obtained with 3 samples measured independently.

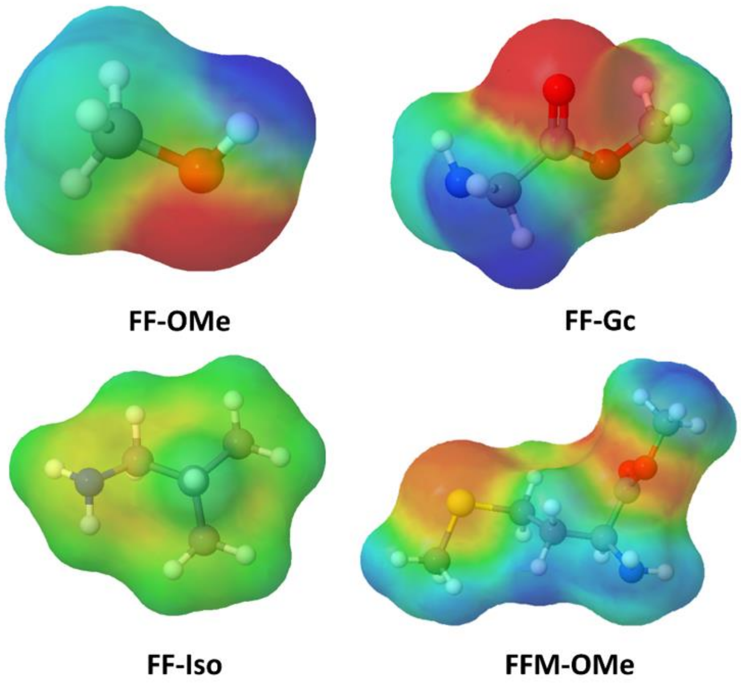

Capping Group	Peptide	Phase transition type	Total solvation energy, ΔG (kJ mol^{-1})	Polar solvation energy, ΔG (kJ mol^{-1})	Non-polar solvation energy, ΔG (kJ mol^{-1})	Surface Area, Å^2
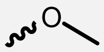	FF-OMe	Coacervate	**-5.4±0.7**	-11.62±0.07	6.3±0.7	181.3±0.7
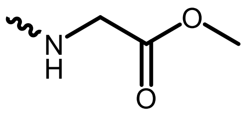	FF-GC	Coacervate	**-11.4±4.0**	-25.6±2.1	14.3±2.5	282.8±8.9
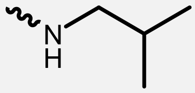	FF-ISO	Solid aggregate	**7.9±1.5**	-8.5±0.2	16.4±1.4	275±12
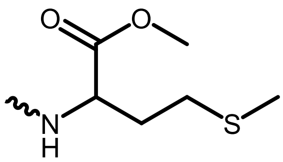	FFM-OMe	Coacervate (transient) / Solid aggregate	**-10.3±2.3**	-40.0±1.4	**29.7±1.5**	401±10

Supplementary Figure 40. Calculation of capping group properties: Solvation free energy and dipole moments of spacers were estimated from PM3 quantum chemical calculations using MolCalc.[1, 6] All structures were first optimised using GAMESS, and dipole moments and solvation surfaces were calculated using PM3 theory. The results are the average of 5 calculations. Electron density maps of several capping groups are shown at the top.

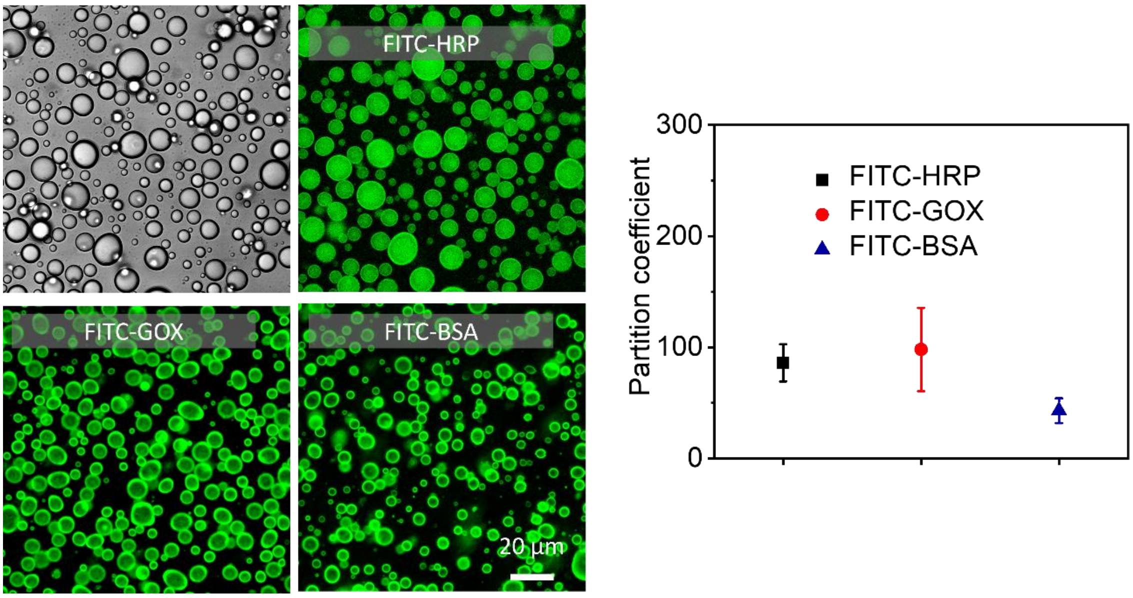

Supplementary Figure 41. Partitioning of proteins in FF-OMe coacervates. Confocal imaging analysis showed that the peptide-coacervates were able to efficiently capture the different proteins. Scale bar=20 μm in all microscopy images. Similar results were obtained with 3 samples measured independently. Data represent mean ± SD for n = 5 representative microscopic images of view.

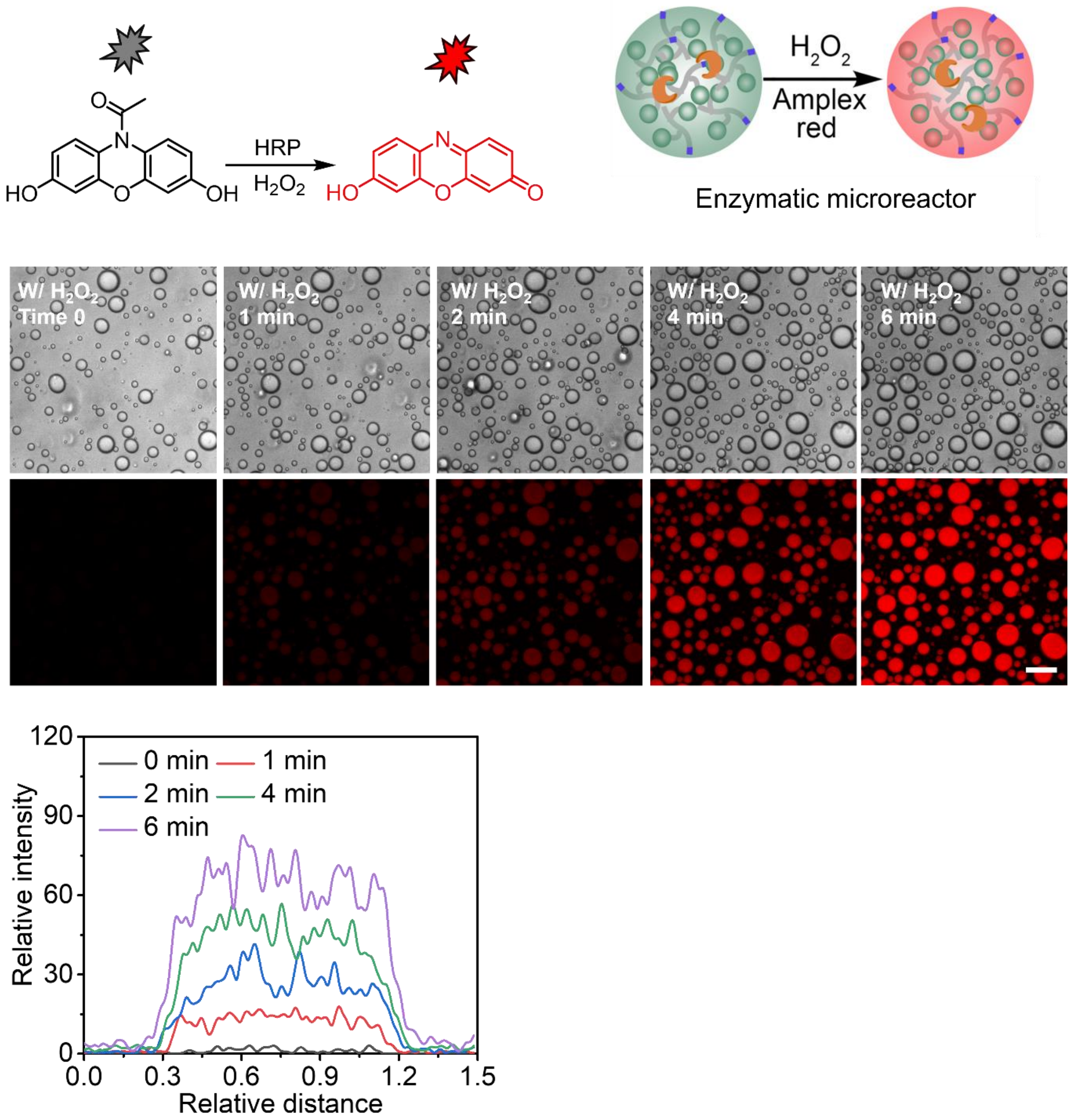

Supplementary Figure 42. Top: Schematic representation of enzymatic microreactors with FF-OMe coacervates containing HRP. Middle: Confocal images of the enzymatic microreactors in the presence of Amplex red (substrate) before and after the addition of H_2O_2, scale bar: 20 μm; Bottom: The fluorescence intensity profile shows a uniform formation of the reaction product within the enzymatic microreactors. Similar results were obtained with 3 samples measured independently.

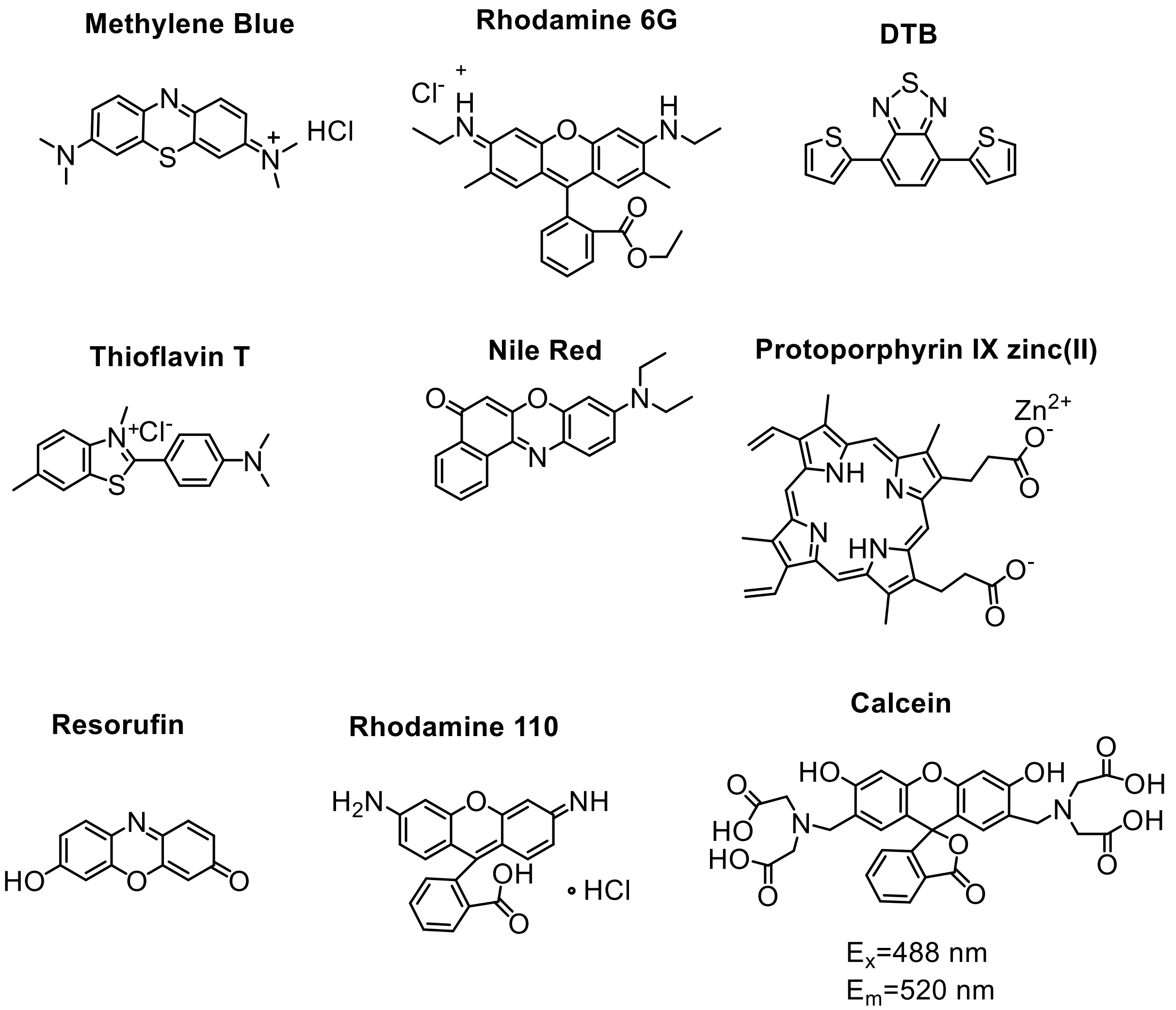

Supplementary Figure 43. Molecular structure of the fluorescent molecules used in the partitioning assays with FF-OMe coacervates.

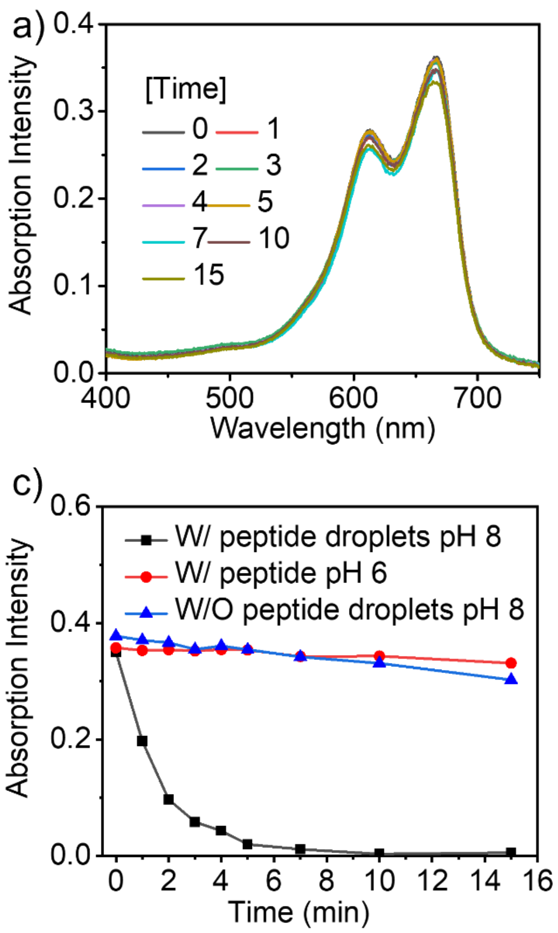

Supplementary Figure 44. Photocatalytic degradation of methylene blue without DTB micro-reactors. a) UV/Vis absorption curve of methylene blue without coacervates at pH ~8 and free DTB photocatalyst; b) UV/Vis absorption curve of methylene blue with fully dissolved FF-OMe (no coacervates) at pH ~6 with free DTB photocatalyst. c) Absorption intensity of methylene blue during photocatalytic degradation under different conditions.

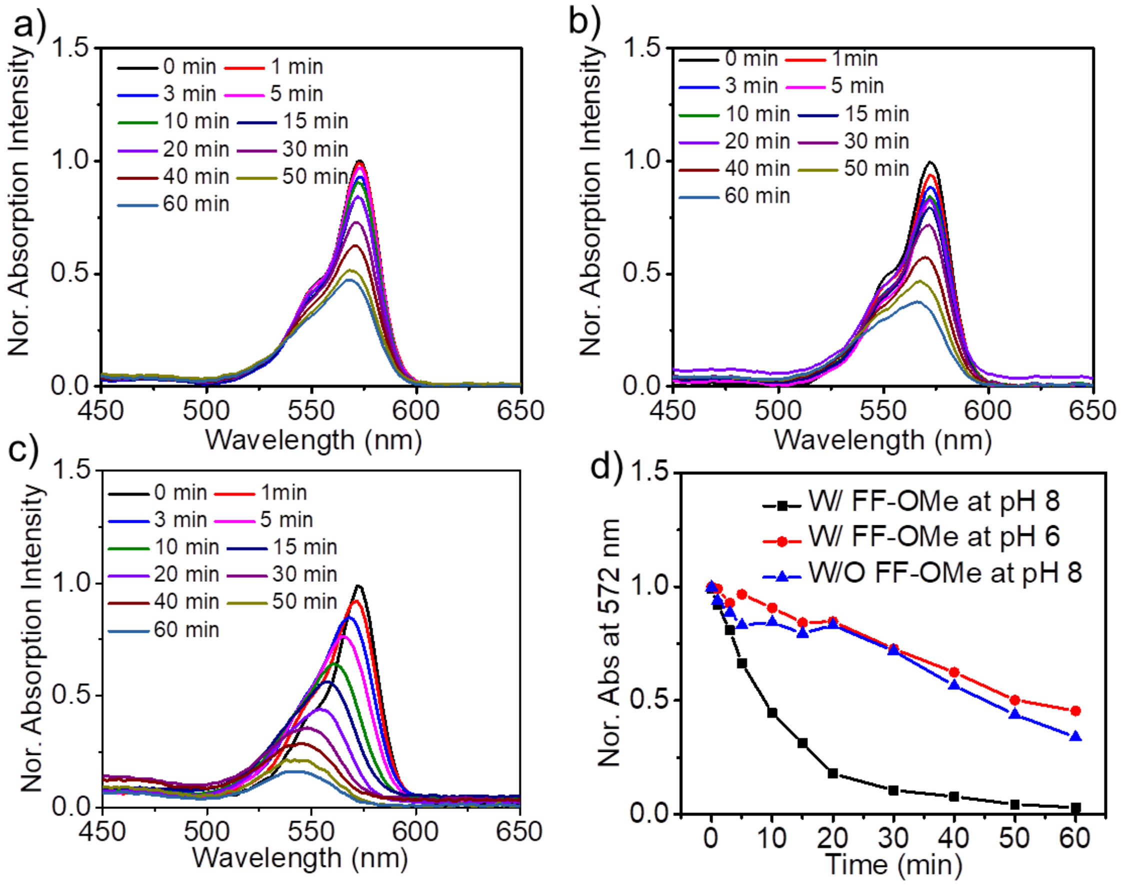

Supplementary Figure 45. Photocatalytic degradation of Rhodamine B under different conditions. a) free FF-OMe, pH 6, free DTB photocatalyst; b) no FF-OMe, pH 8, free DTB; c) DTB microreactors, pH 8; d) normalized absorption intensities over time at λ_{abs}=572 nm.

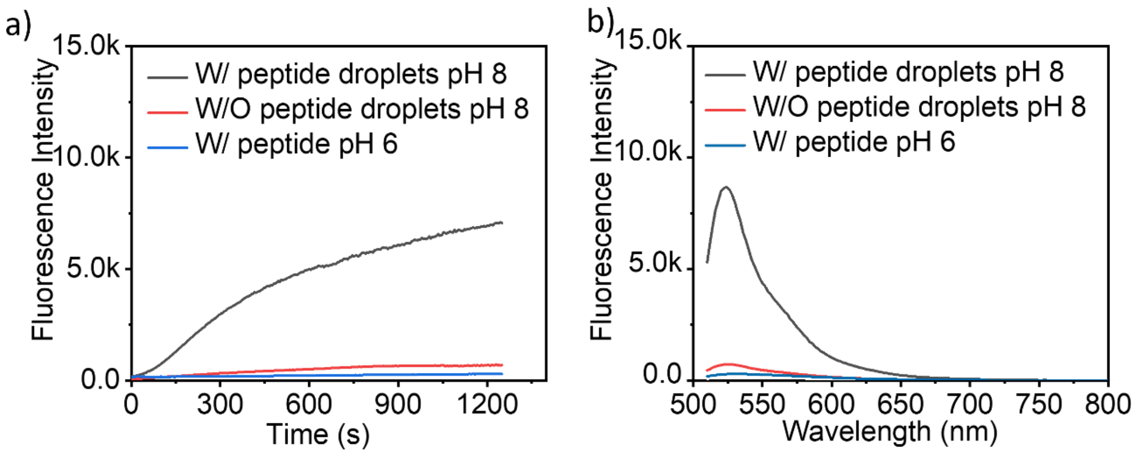

Supplementary Figure 46. The Ru-microreactor causes the decaging of the rhodamine-110-based pro-fluorophore (Rho-pro), restoring the strong fluorescence; (a) the fluorescence intensity of Rho-pro increased significantly only after incubation with the Ru-microreactor; (b) the fluorescence emission intensity of Rho-110 after 20 min under different conditions.

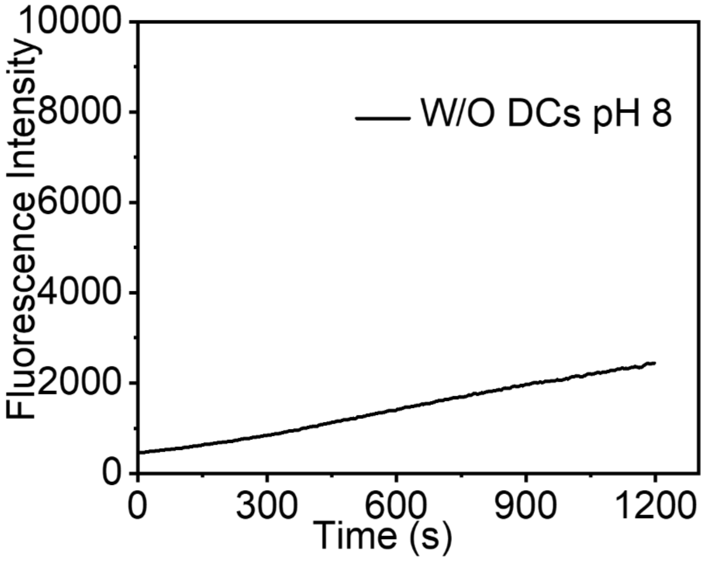

Supplementary Figure 47. Ru-catalysed uncaging reaction of caged Rho-110 with 5-fold free Ru catalyst concentration (with 0.025 mg mL^{-1} Ru) and substrate Rho-pro (0.25 mg mL^{-1}) in the absence of dipeptide coacervates (DCs).

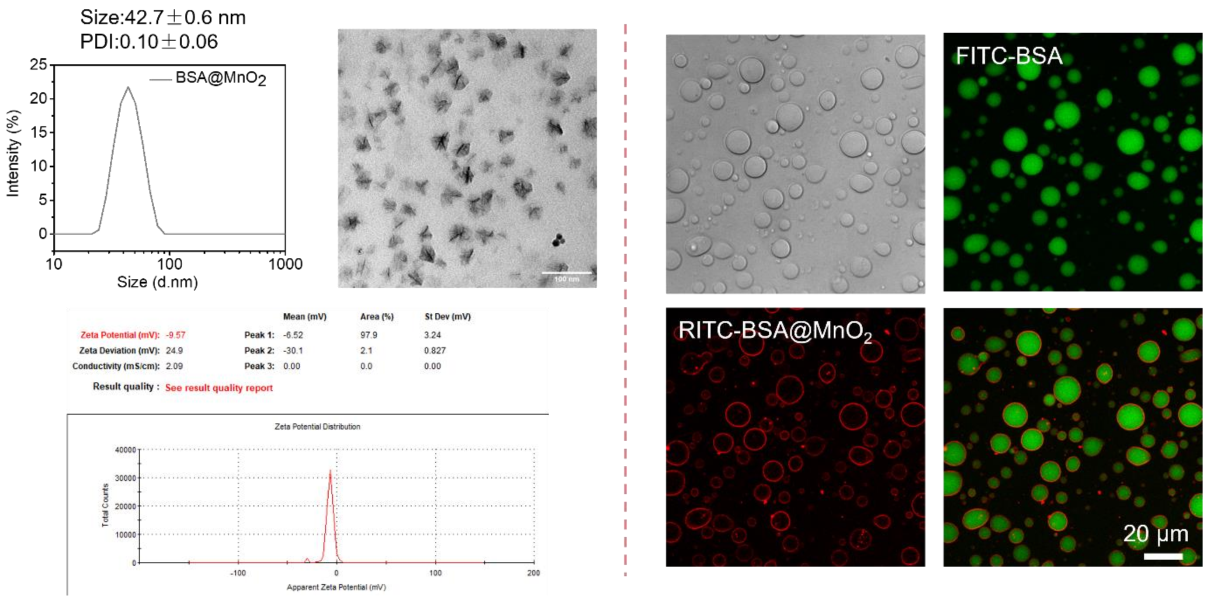

Supplementary Figure 48. Left: DLS, TEM and zeta measurement of BSA@MnO_2 nanoparticles, which have a size of about 40 nm and negative charge of -11.0+2.5 mV, scale bar=100 nm in the TEM image. Right: BSA@MnO_2 stabilized complex coacervates. BSA@MnO_2 particles are located on the surface, while labelled BSA (FITC-BSA) are sequestered inside the complex coacervates for better visualization. Scale bar=20 μm in all microscopy images. Similar results were obtained with 3 samples measured independently.

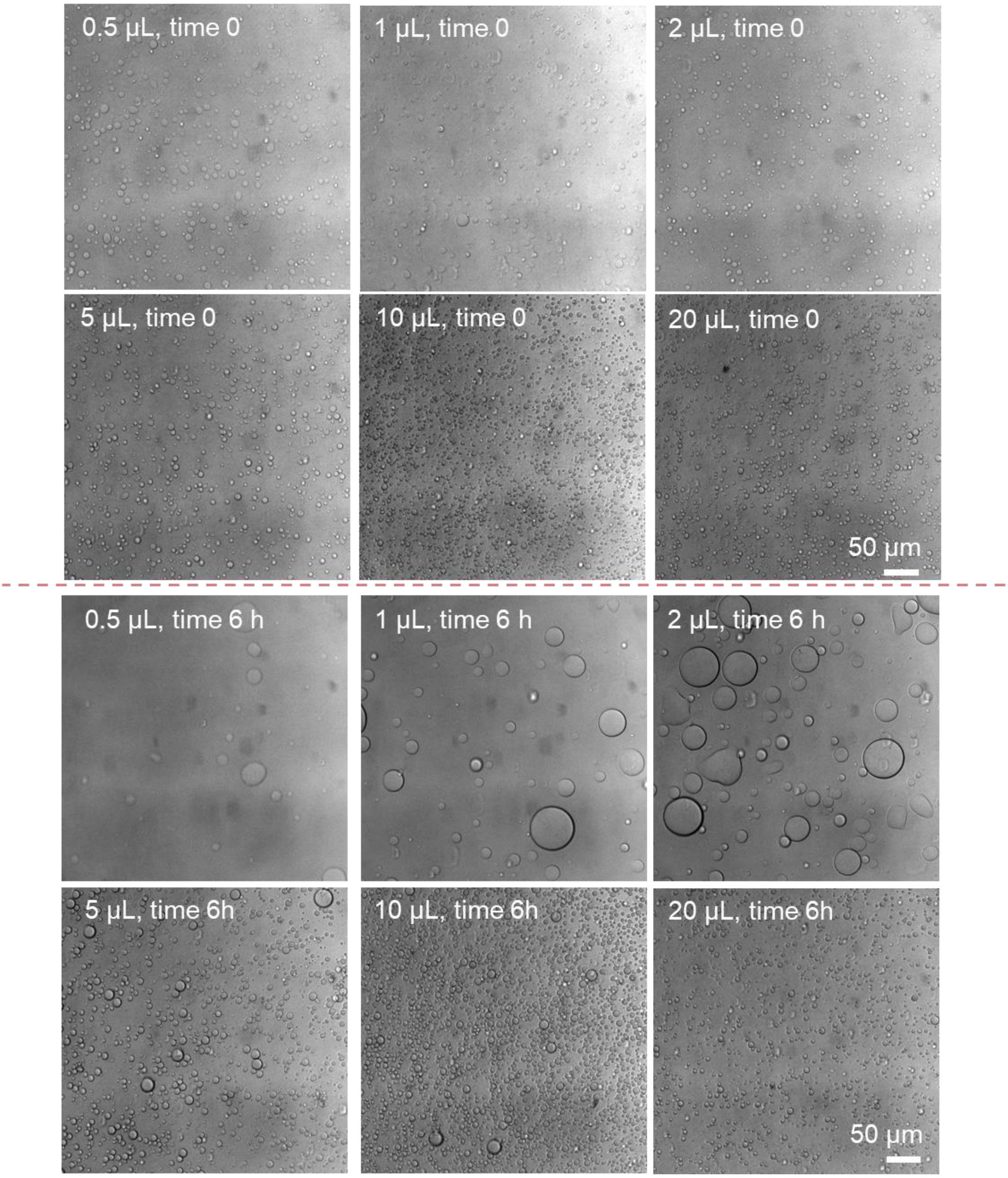

Supplementary Figure 49. Top: Different volumes of BSA@MnO2 (0.7 mg mL^{-1}) were used to stabilize the complex coacervates formed with 10 µL Q-AM (2.5 mg mL^{-1}) and 10 µL C-AM (2.5 mg mL^{-1}) at the beginning of the measurement. Bottom: The above solutions were incubated for 6 h. Microscopic imaging shows that a volume of BSA@MnO_2 below 5 µL is not able to sufficiently stabilize the coacervates: coacervates coalescence and fusing were observed. After treatment of the complex coacervates with a volume of BSA@MnO_2 around or higher than 5 µL, the complex coacervates maintain their initial size, indicating interfacial stabilization. Scale bar=50 µm in all microscopy images. Similar results were obtained with 3 samples measured independently.

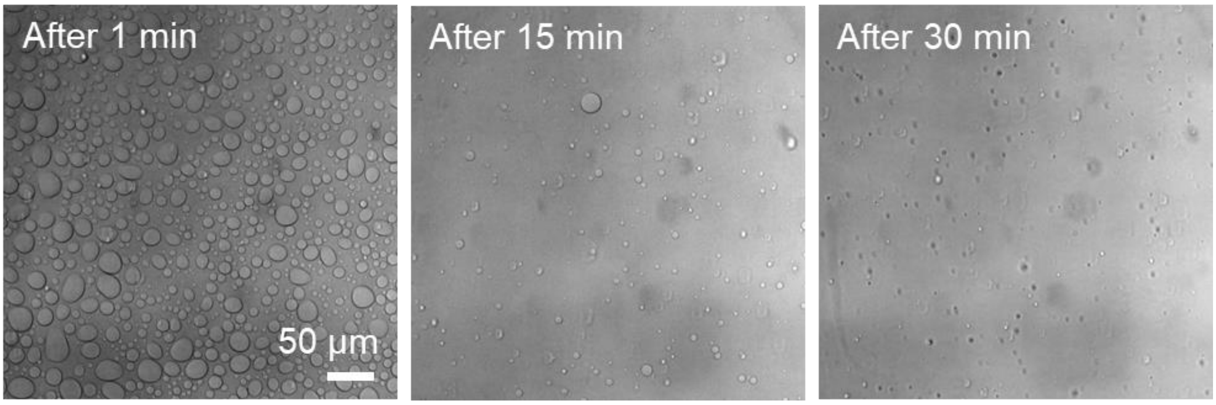

Supplementary Figure 50. Complex coacervates of Q-AM and C-AM were treated with similar amounts of BSA, but no surface stabilization was observed. After 30 minutes, most of the complex coacervates had coalesced and adhered to the glass surface. Scale bar=50 μm in all microscopy images. Similar results were obtained with 3 samples measured independently.

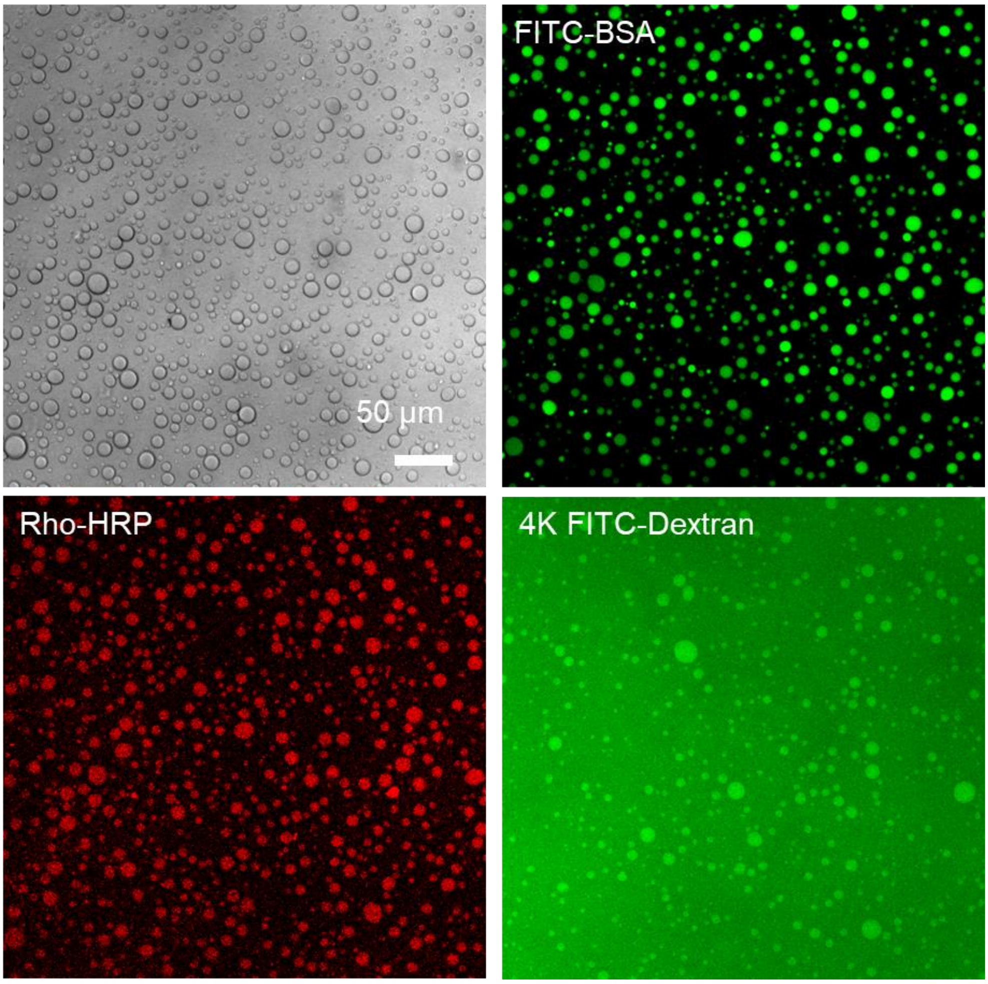

Supplementary Figure 51. After surface stabilization, the BSA@MnO_2 stabilized complex coacervates showed permeability to proteins, FITC-BSA and Rho-HRP, and 4 KDa FITC-dextran. Scale bar=50 μm in all microscopy images. Similar results were obtained with 3 samples measured independently.

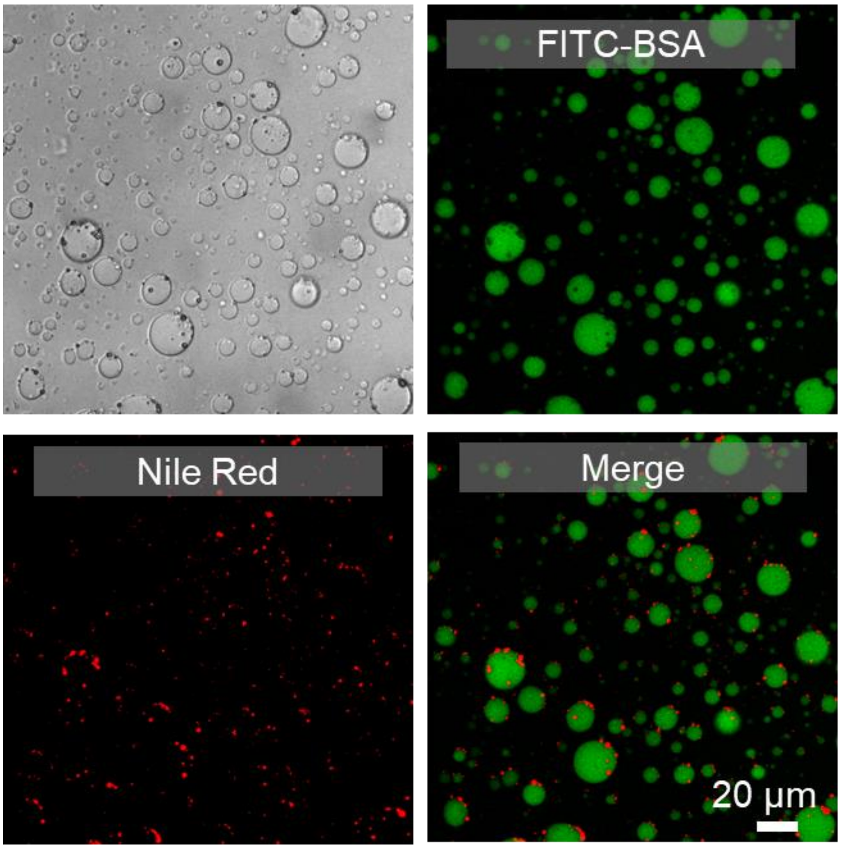

Supplementary Figure 52. FF-OMe coacervates were integrated as organelles within complex coacervates. The different compartments were stained with Nile Red (organelles) and FITC-BSA (“cytosol”). Scale bar=20 µm in all microscopy images. Similar results were obtained with 3 samples measured independently.

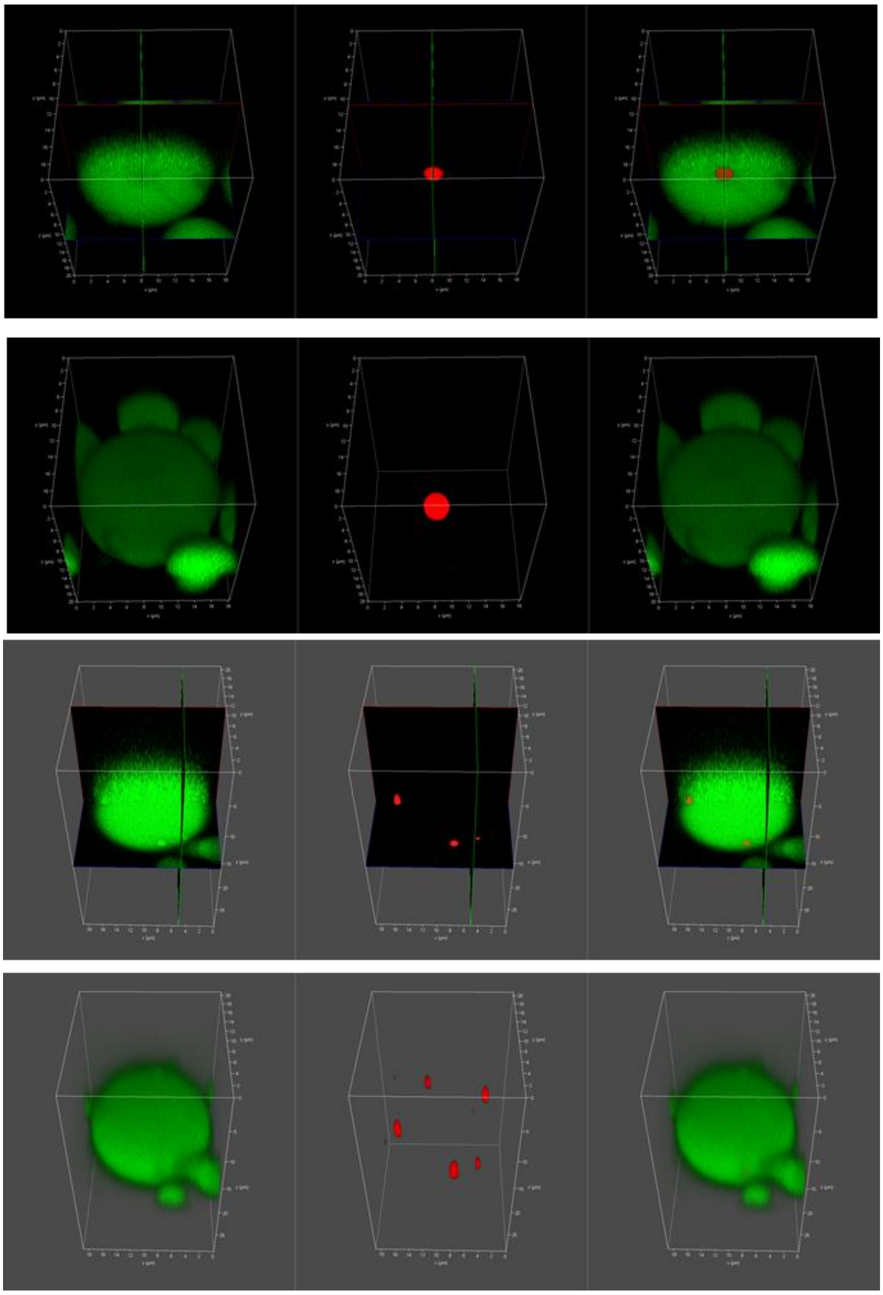

Supplementary Figure 53. 3D cross-sectional images and reconstruction images of synthetic cells formed by integrating peptide coacervates into membrane-bound complex coacervates. Green channel: FITC-BSA; Red channel: Nile Red. Similar results were obtained with 3 samples measured independently.

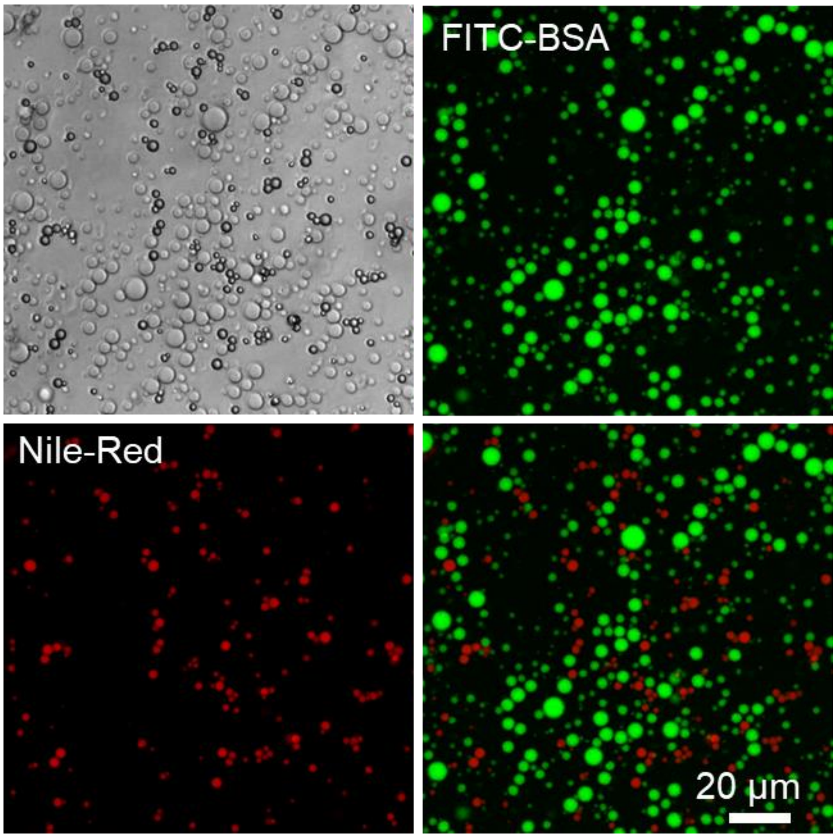

Supplementary Figure 54. Mixing the peptide coacervates with membrane-bound complex coacervates resulted in two populations of droplets. Scale bar=20 μm in all microscopy images. Similar results were obtained with 3 samples measured independently.

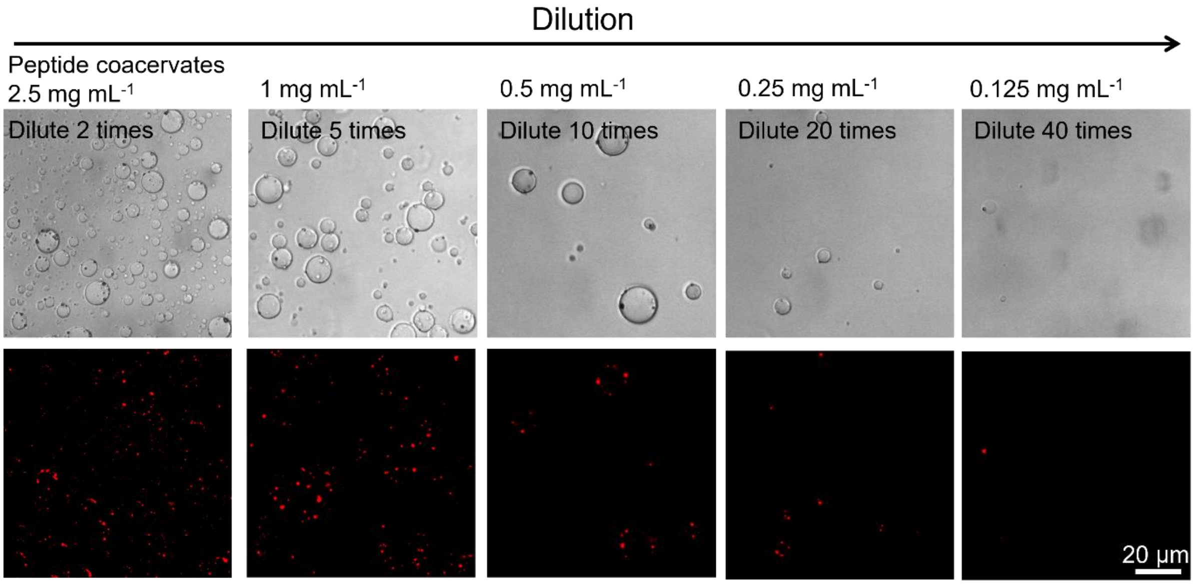

Supplementary Figure 55. Dipeptide coacervates in synthetic cells show improved stability against dilution. The emission of Nile Red in dipeptide coacervates can be observed at concentrations as low as 0.125 mg mL^{-1}. Scale bar=20 μm in all microscopy images. Similar results were obtained with 3 samples measured independently.

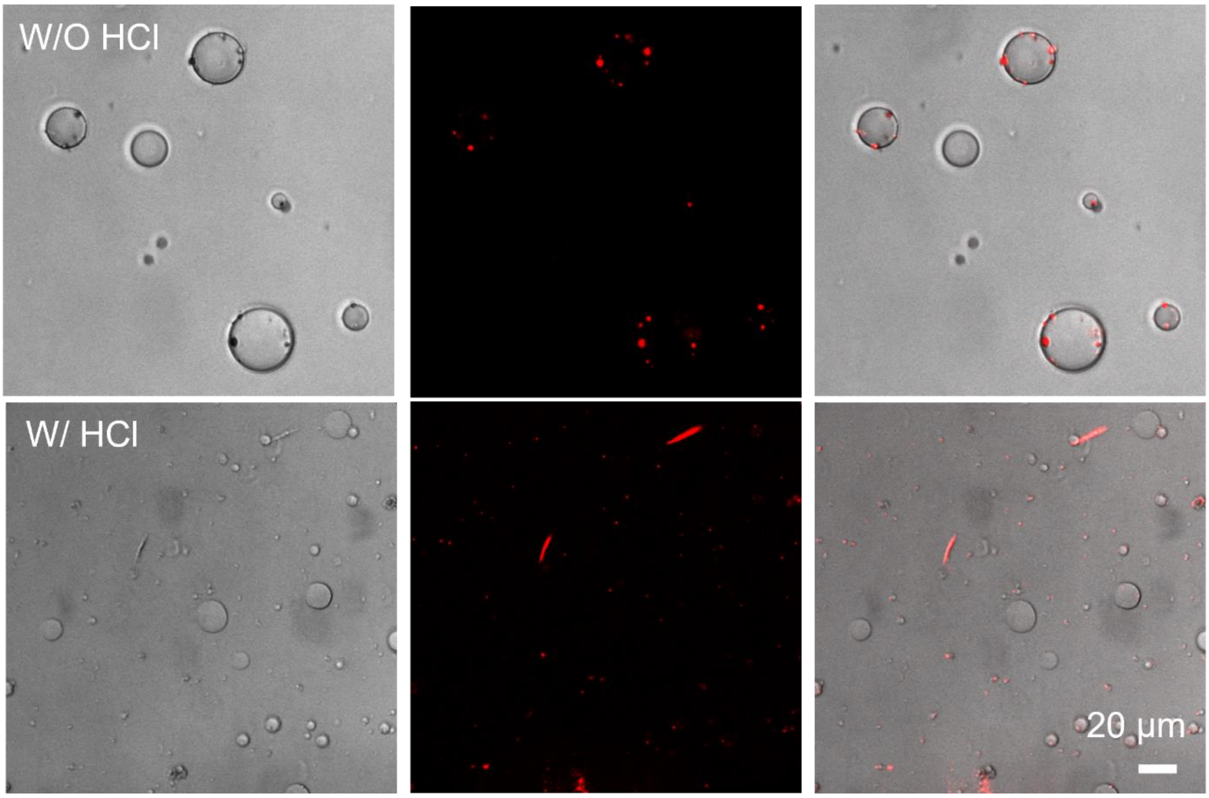

Supplementary Figure 56. Integration of dipeptide coacervates into synthetic cells did not affect their pH responsiveness. Prior to the addition of HCl, Nile Red-loaded dipeptide coacervates were located inside the synthetic cells. The addition of HCl caused the internal droplets to disintegrate. Scale bar=20 μm in all microscopy images. Similar results were obtained with 3 samples measured independently.

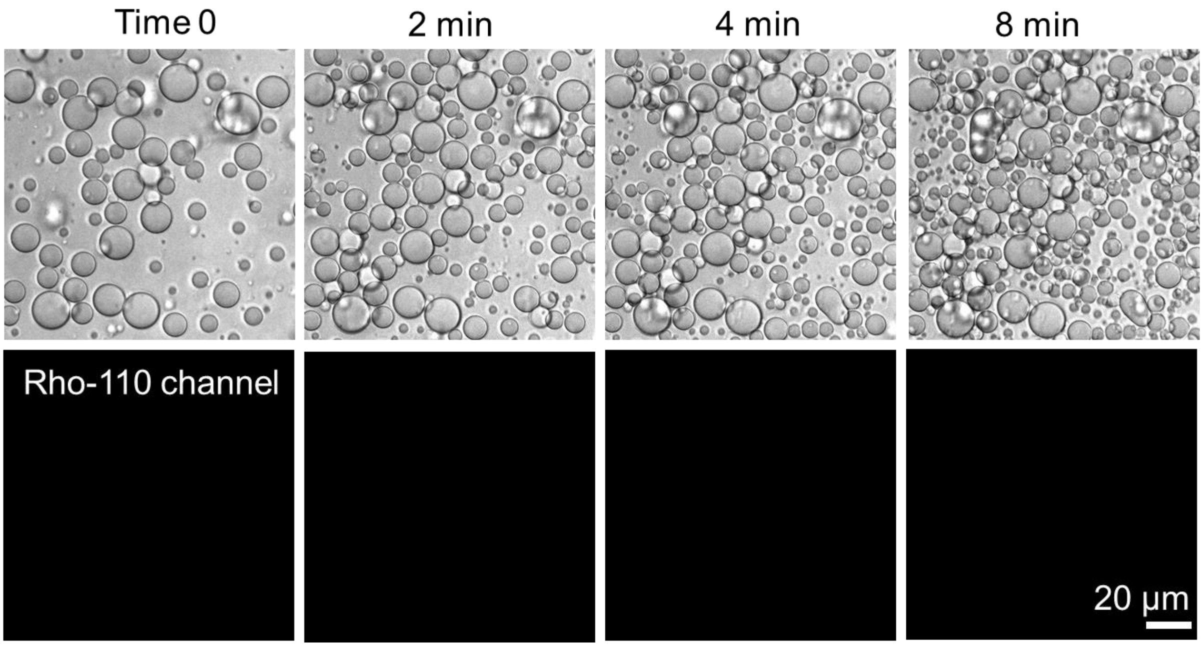

Supplementary Figure 57. Synthetic cells lacking Ru organelles were fed with Ru catalyst and the substrate Rho-pro. Confocal imaging shows no significant product formation in the absence of Ru organelles. Scale bar=20 μm in all microscopy images. Similar results were obtained with 3 samples measured independently.

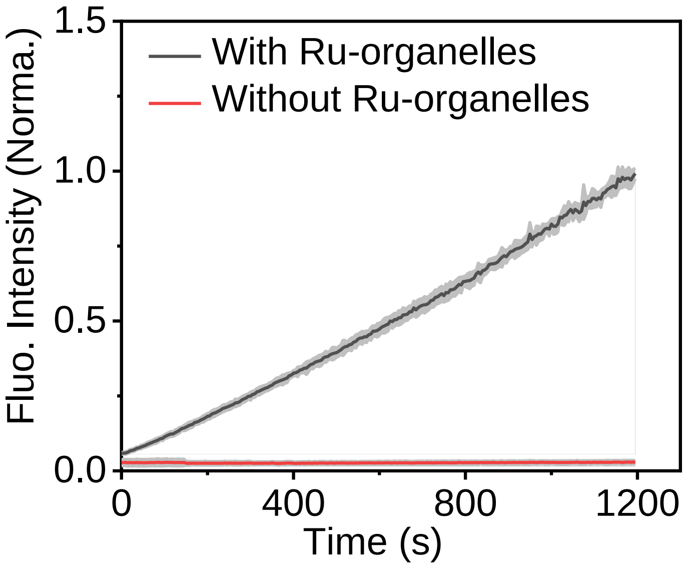

Supplementary Figure 58. Microplate reader assay for the decaging reaction by cell mimics with and without Ru-organelles. The results indicate that the Ru-organelle is necessary for the reaction to occur. Data represent the mean ± SD for n = 3 independent samples.

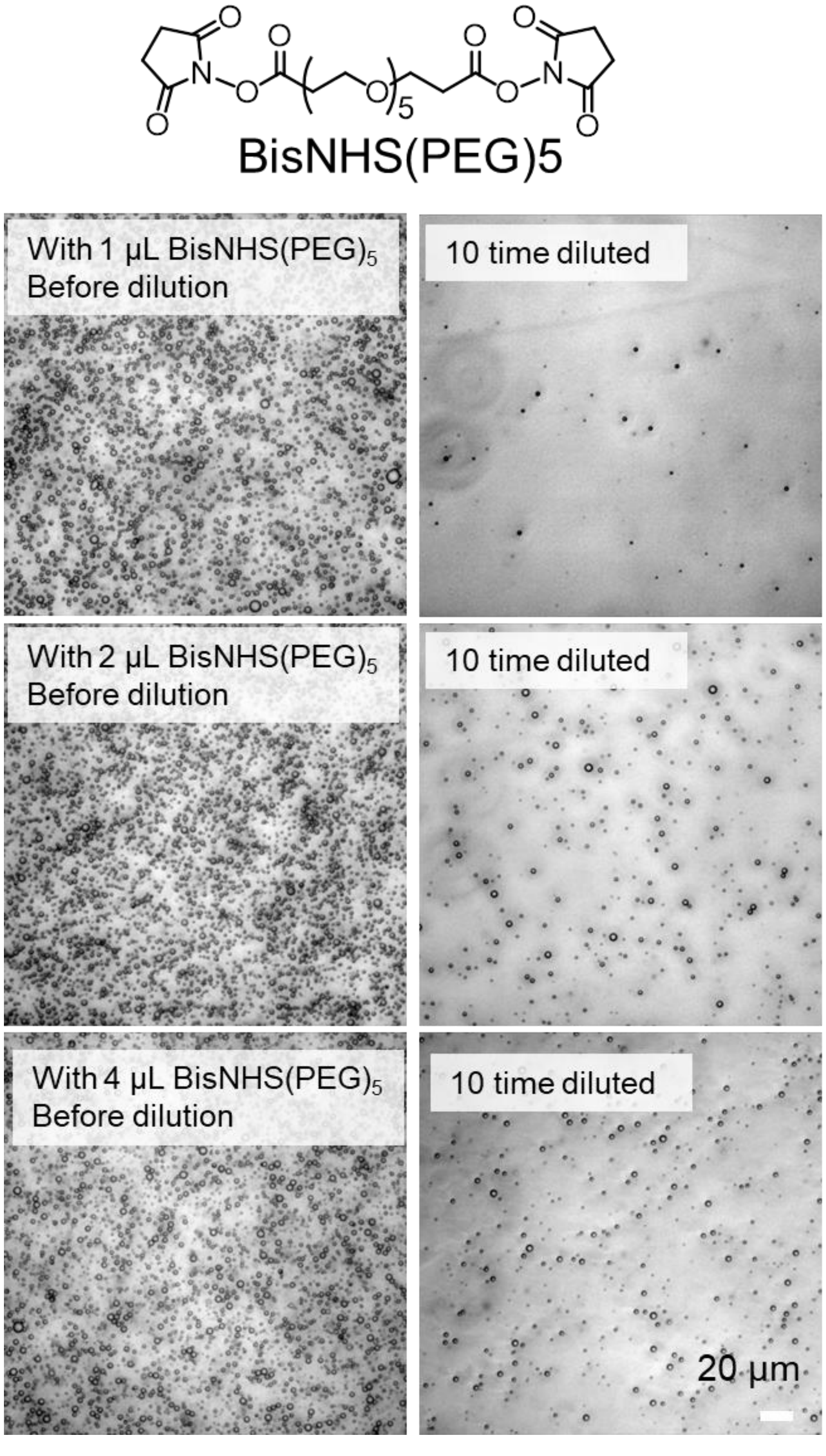

Supplementary Figure 59. Stability of FF-OMe coacervates (100 μL, 5 mg mL^{-1}) after dimerization with different amounts of BisNHS$(PEG)_5$ (100 mg mL^{-1}). The dimerization time was 0.5 hours. After dimerization with ≥2 μL BisNHS$(PEG)_5$, the peptide coacervates showed desirable stability after dilution. Scale bar=20 μm in all microscopy images. Similar results were obtained with 3 samples measured independently.

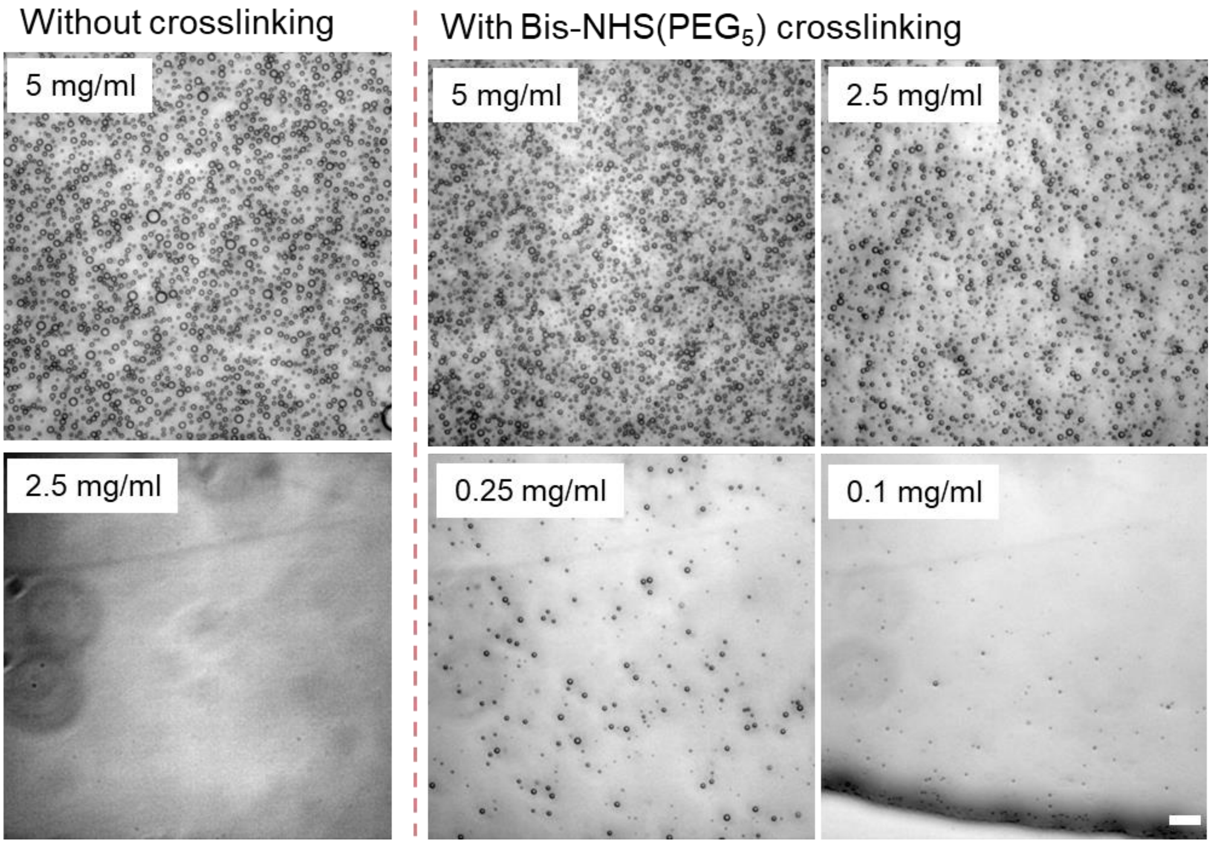

Supplementary Figure 60. Stability of dipeptide coacervates before and after dimerization with 2 μL BisNHS(PEG)$_5$. Before dimerization, the coacervates dissolved in buffer when diluted to 2.5 mg mL^{-1}. Dimerized coacervates were observed even after dilution down to 0.1 mg mL^{-1}, scale bar=20 μm. Similar results were obtained with 3 samples measured independently.

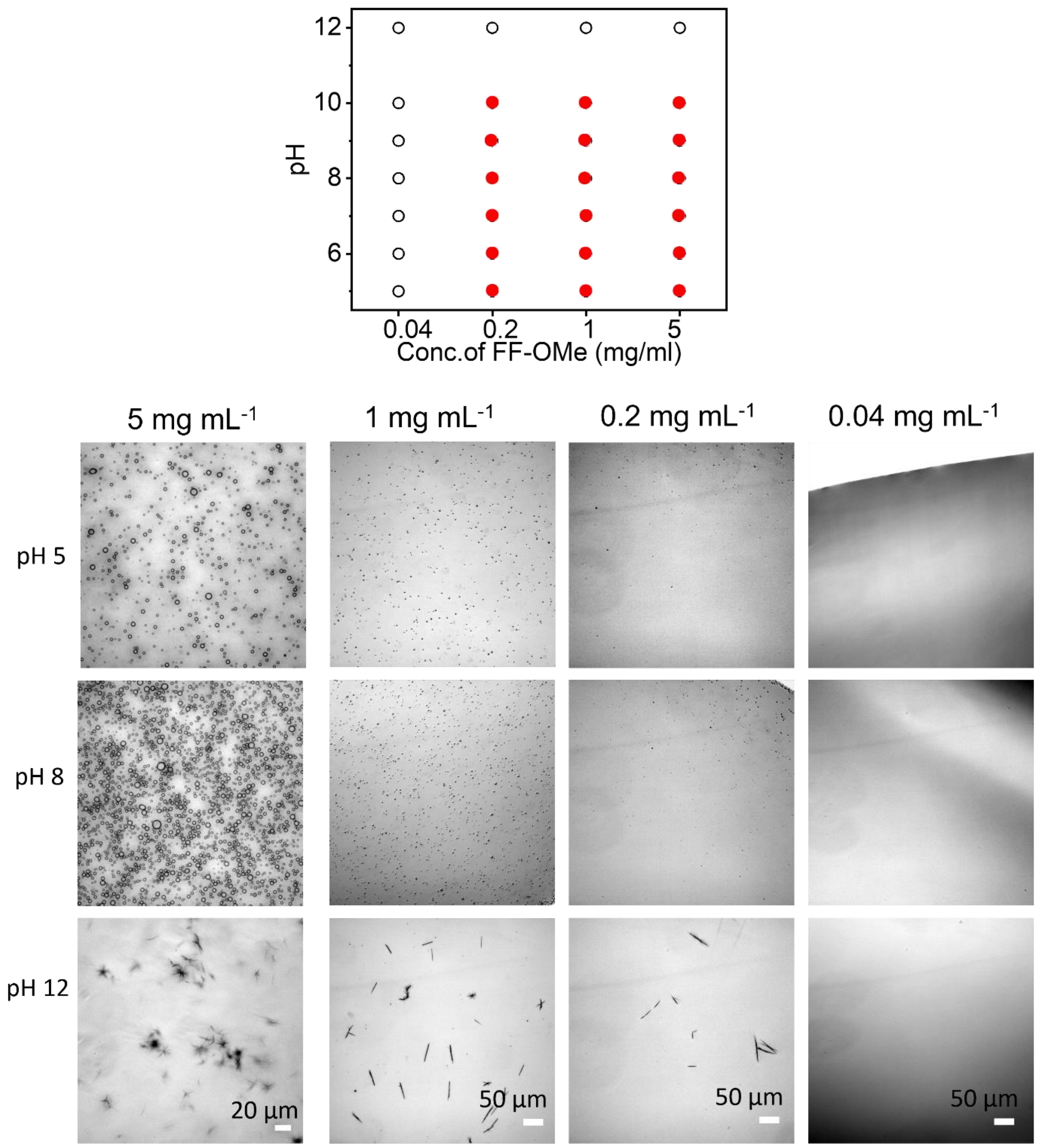

Supplementary Figure 61. Phase diagram of the dimerized peptide coacervates (dimerization with 2 µL BisNHS(PEG)$_5$ (100 mg mL^{-1}). Dimerization increased the stability of the coacervates, which was observed even in acid pH solutions (pH 5) with a concentration down to 0.2 mg mL^{-1}. However, strong alkaline pH (pH ~12) induced fiber transition. Scale bar=50 µm in all microscopy images. Similar results were obtained with 3 samples measured independently.

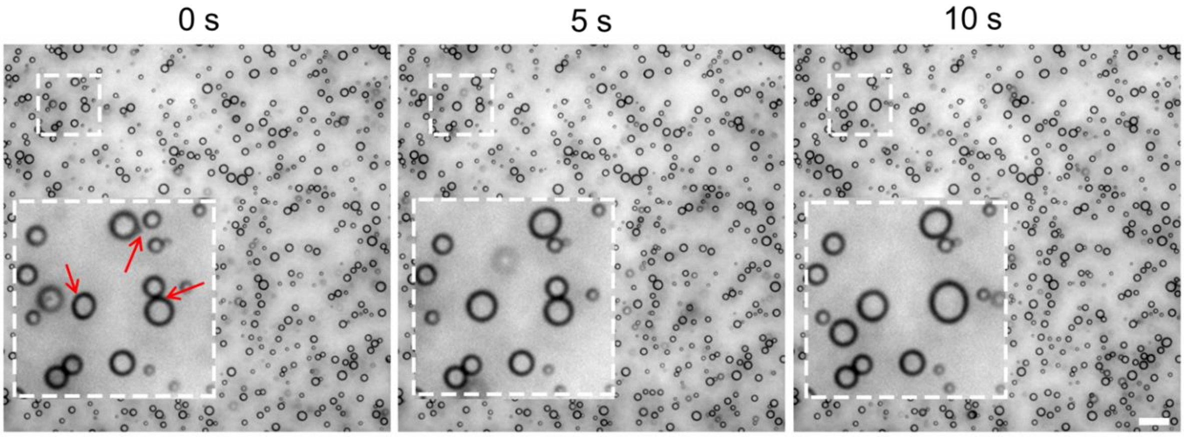

Supplementary Figure 62. The ability of dipeptide coacervates to coalesce was maintained after dimerization with 2 µL BisNHS(PEG)$_5$ (100 mg mL^{-1}), scale bar=20 µm. Similar results were obtained with 3 samples measured independently.

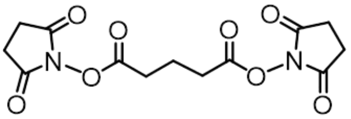

Bis-NHS-C3

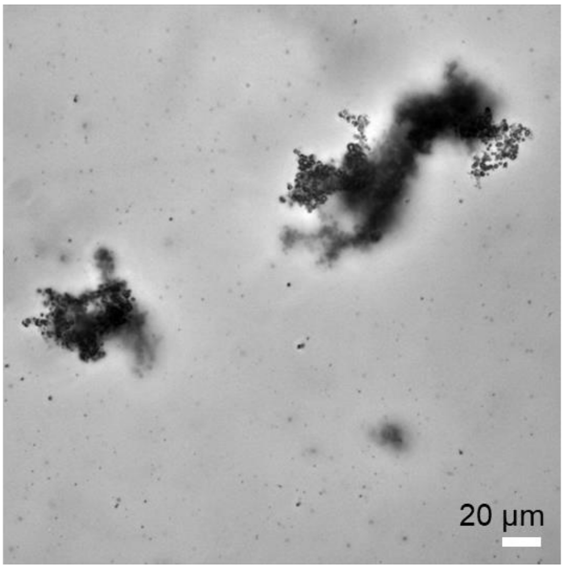

Supplementary Figure 63. The FF-OMe coacervates (5 mg mL^{-1}) were also dimerized with Bis-NHS-C3. However, only aggregates were observed after treatment. Scale bar=20 µm in all microscopy images. Similar results were obtained with 3 samples measured independently.

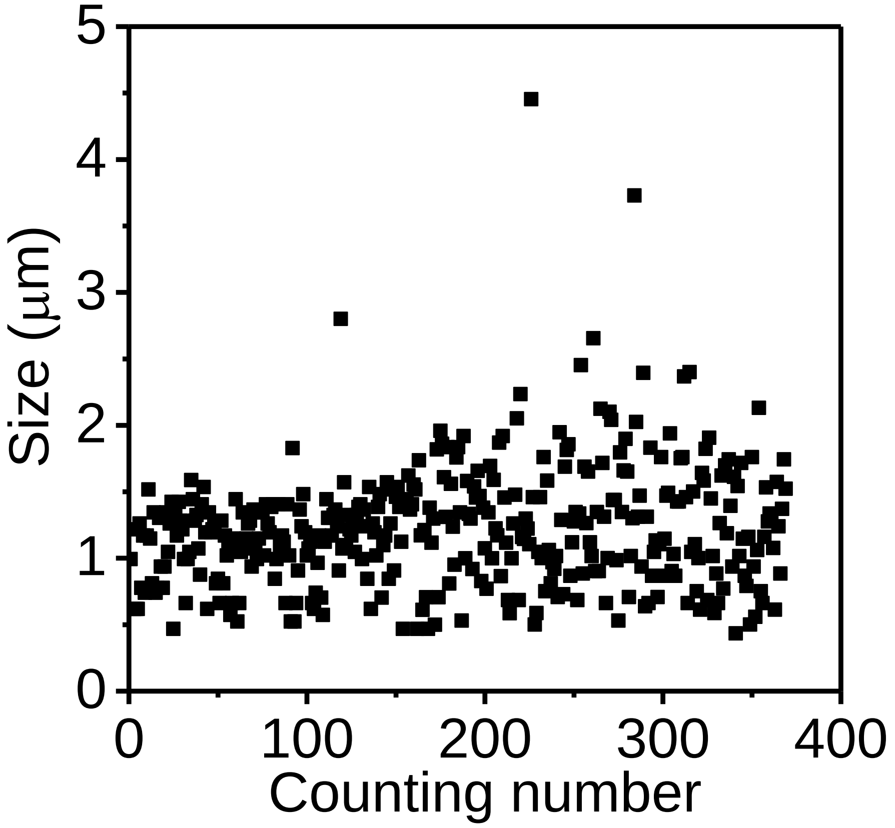

Supplementary Figure 64. Size distribution of dimerized dipeptide coacervates by manual counting. Measurements were performed on coacervates loaded with Nile Red. Analysis was performed using Image J.

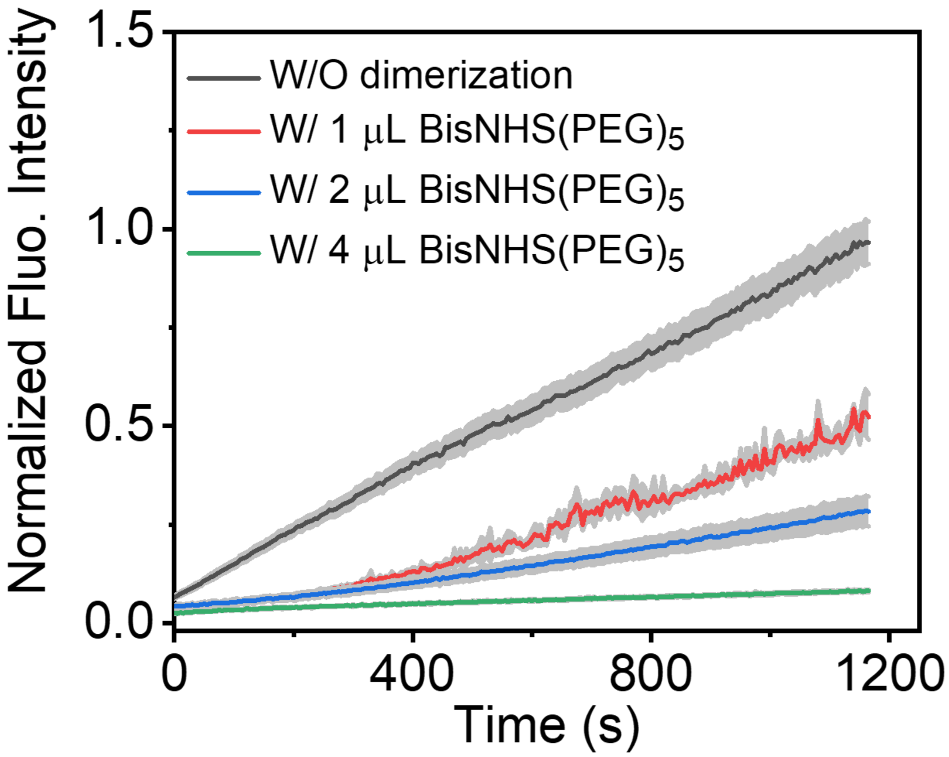

Supplementary Figure 65. Uncaging reaction of Rho-pro using Ru-organelles with different degrees of dimerization. The Ru-organelles were dimerized with different amounts of BisNHS(PEG)$_5$ (100 mg mL^{-1} in DMSO) for 0.5 h. The uncaging reaction of Rho-pro was monitored by microplate reader under periodic shaking. Based on the fluorescence intensity of the restored Rho 110 emission, dimerization with a volume of ~ 2 μL of BisNHS(PEG)$_5$ (100 mg mL^{-1} in DMSO) still retained the catalytic activity. Data represent the mean ± SD for n = 3 independent samples.

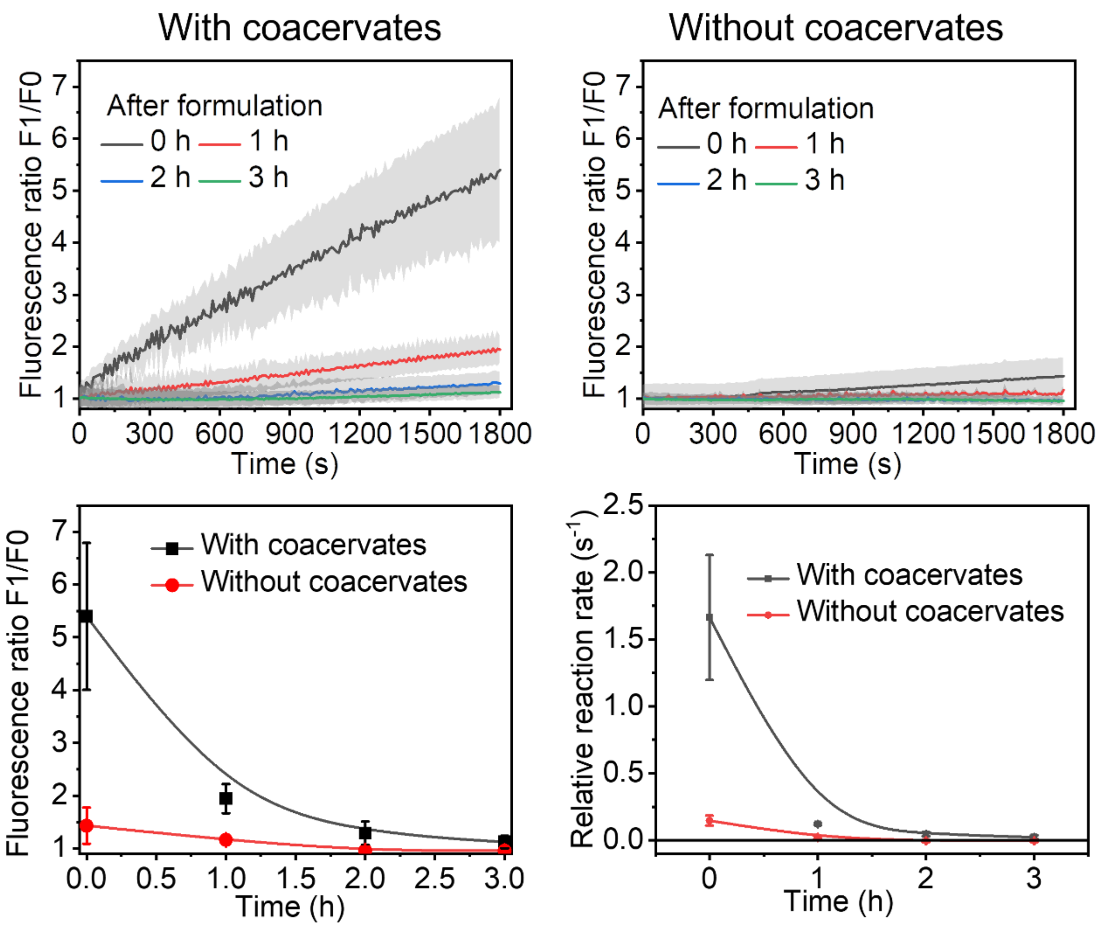

Supplementary Figure 66. Decaging reaction of Rho-pro using Ru organelles and free Ru. The Ru-organelles were dimerized with 2μ BisNHS(PEG)$_5$ (100 mg mL-1 in DMSO). The reaction of Rho-pro was monitored by microplate reader with periodic shaking. Incorporation of the catalyst into the peptide coacervates can greatly enhance the catalytic efficiency, as visualized by the ~5.4-fold increase in fluorescence intensity of the decaged product (Rho-110) with freshly prepared dimerized coacervates, compared to the ~1.4-fold increase in fluorescence intensity of the unmodified catalyst group. After 2 hours of preparation, there is almost no increase in fluorescence intensity after the decaging reaction in the naked catalyst group, indicating that the catalyst has almost lost its activity. However, in the presence of the coacervates, there is still an increase in fluorescence intensity even after 3 hours of sample preparation. This indicates that the presence of coacervates helps to improve the catalytic efficiency and prolong the life of the catalyst. Data represent the mean ± SD for n = 3 independent samples.

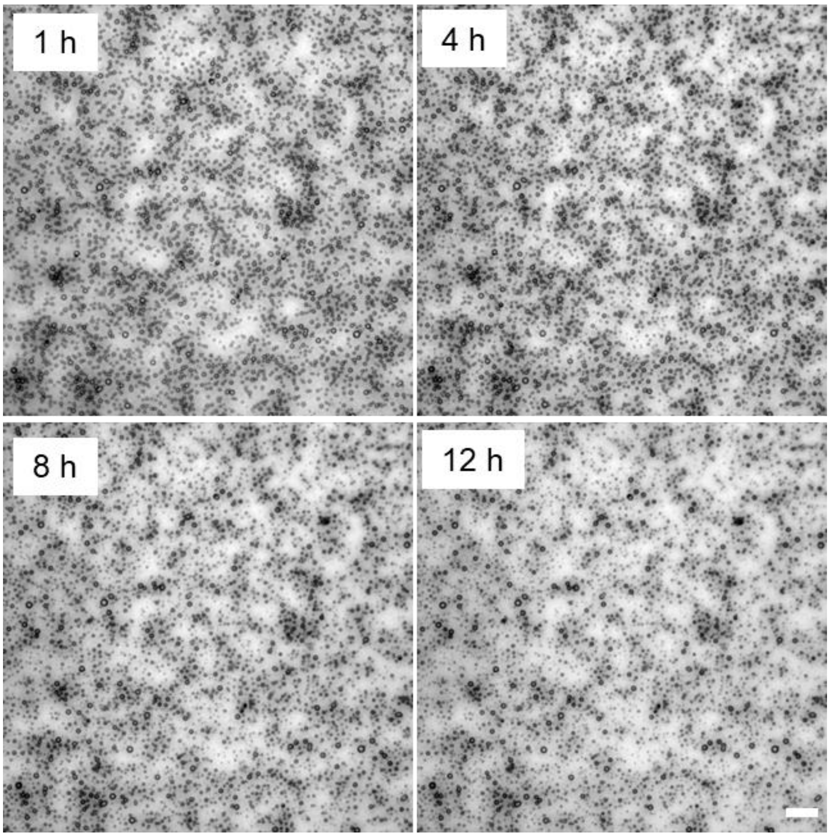

Supplementary Figure 67. Stability of dimerized FF-OMe coacervates (0.5 mg mL^{-1}) incubated with complete cell culture medium. Most of the coacervates retain their integrity, indicating their stability and suitability for cell culture, scale bar=20 μm. Similar results were obtained with 3 samples measured independently.

			Mean (mV)	Area (%)	St Dev (mV)
Zeta Potential (mV):	-5.54	Peak 1:	-5.54	100.0	4.49
Zeta Deviation (mV):	4.49	Peak 2:	0.00	0.0	0.00
Conductivity (mS/cm):	2.67	Peak 3:	0.00	0.0	0.00

Result quality : See result quality report

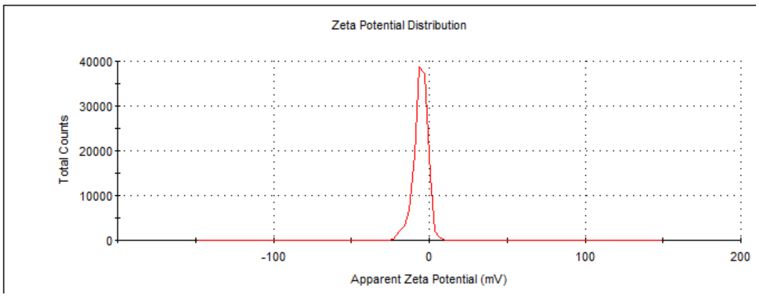

			Mean (mV)	Area (%)	St Dev (mV)
Zeta Potential (mV):	-6.40	Peak 1:	-7.48	95.3	3.71
Zeta Deviation (mV):	6.06	Peak 2:	15.4	4.7	1.88
Conductivity (mS/cm):	2.97	Peak 3:	0.00	0.0	0.00

Result quality : Good

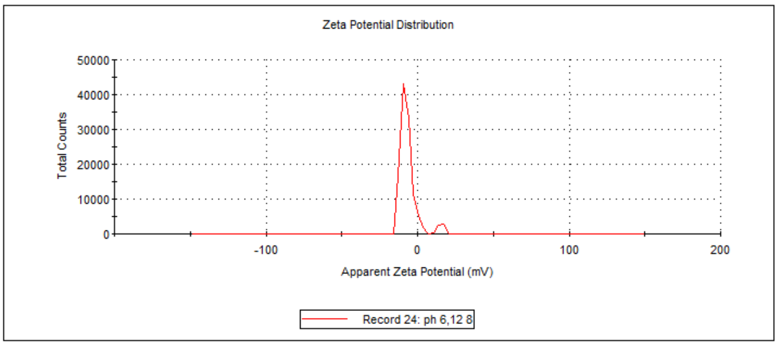

Supplementary Figure 68. Top: Zeta potential of dimerized FF-OMe coacervates. Bottom: Zeta potential of non-treated FF-OMe coacervates.

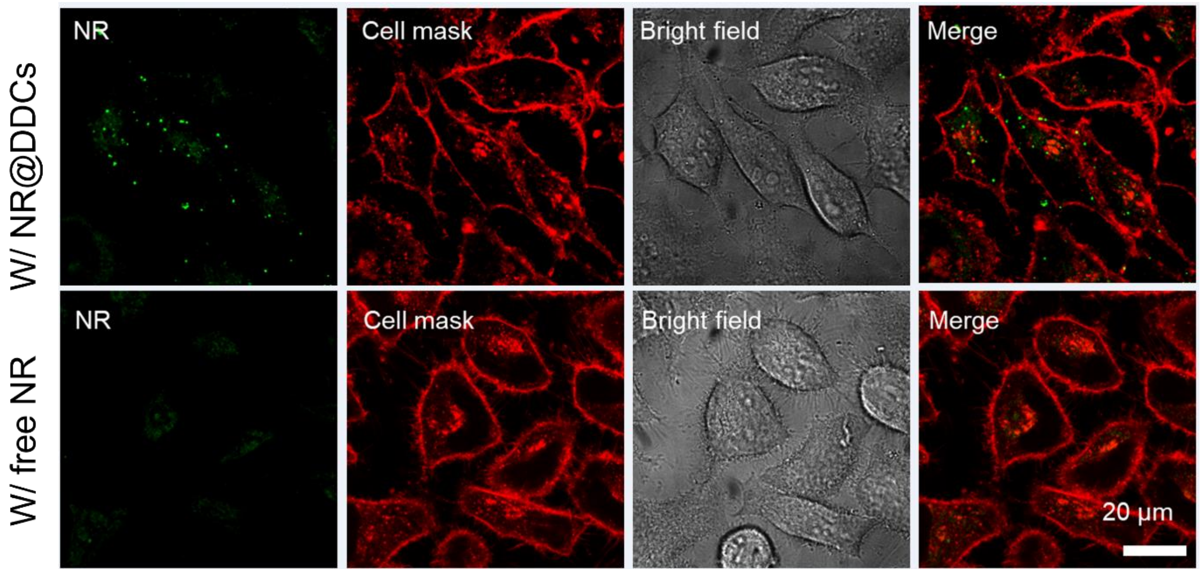

Supplementary Figure 69. Comparison of cellular uptake of free Nile Red and Nile Red-loaded dimerized dipeptide coacervates (DDCs). Scale bar=20 μm in all microscopy images. Similar results were obtained with 3 samples measured independently.

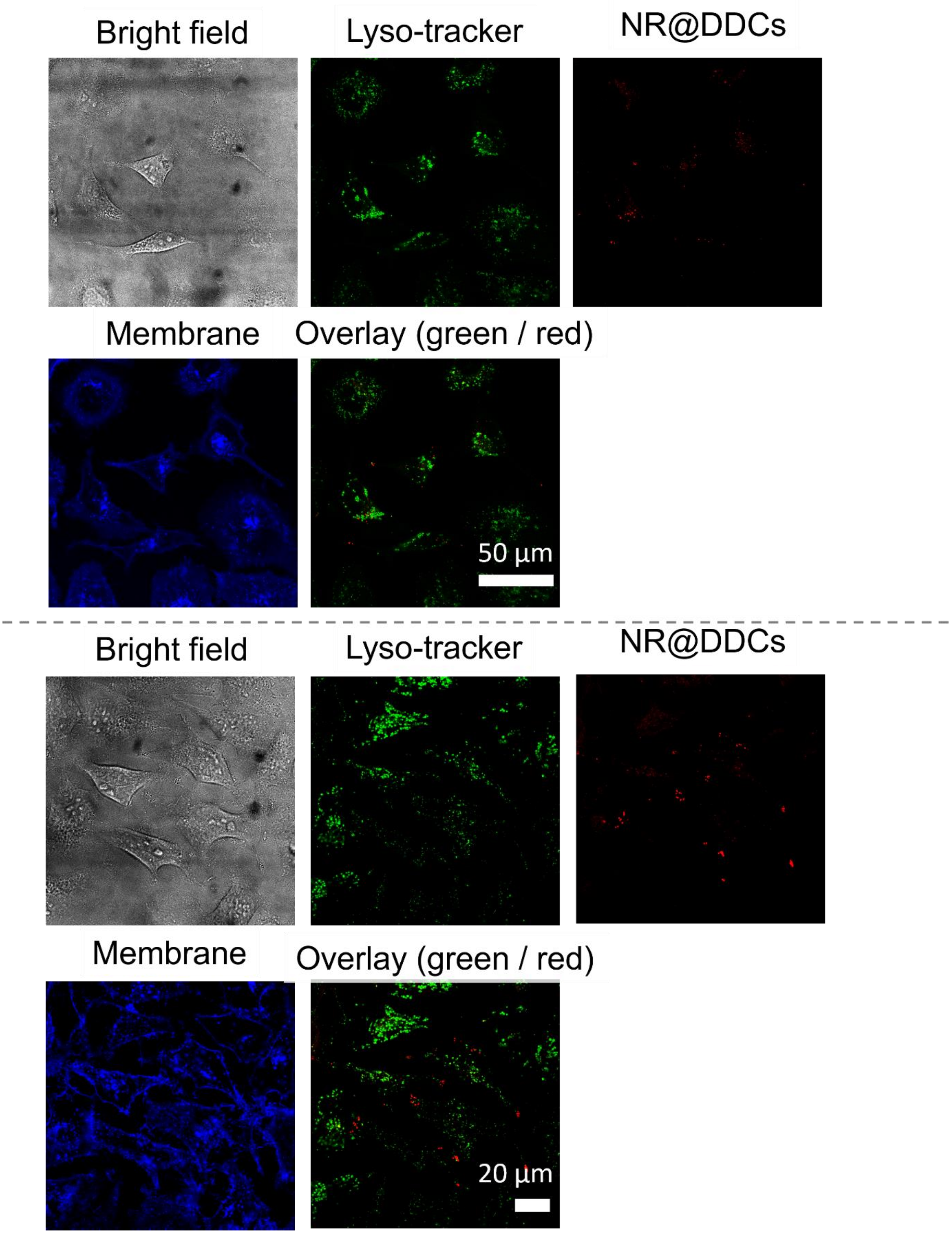

Supplementary Figure 70. Cellular internalization of DDCs, cells were stained with Lysol Tracker and cell mask. The top and bottom confocal images are shown to demonstrate that the peptide organelles were randomly distributed within the cells and were not enriched in lysosomal enriched regions. Scale bar=20 μm in all microscopy images. Similar results were obtained with 3 samples measured independently.

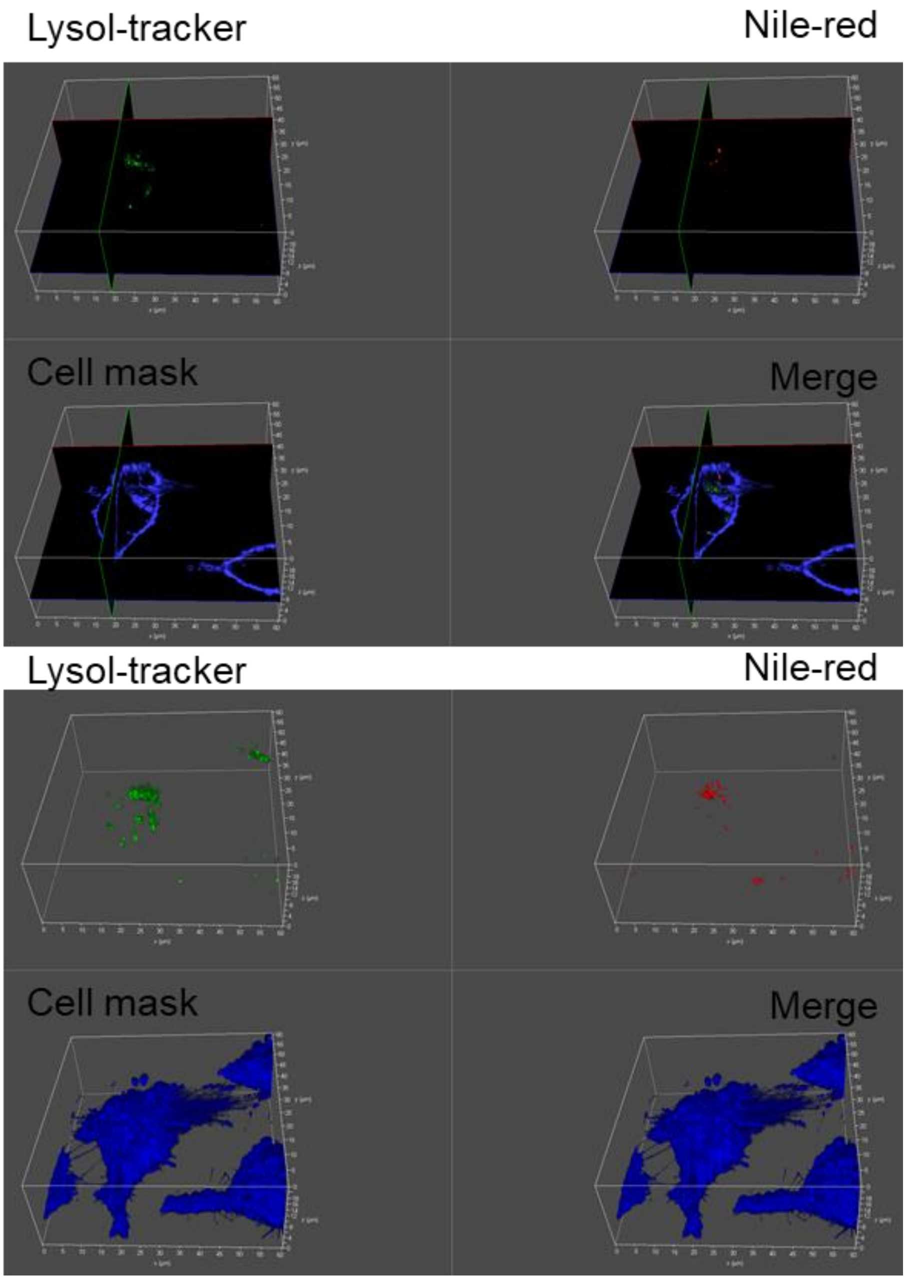

Supplementary Figure 71. 3D cross-sectional and reconstruction images of the internalized dimerized dipeptide coacervates inside HeLa cells. Green channel: Lysol Tracker; Red channel: Nile Red; Blue channel: Cell mask. Similar results were obtained with 3 samples measured independently.

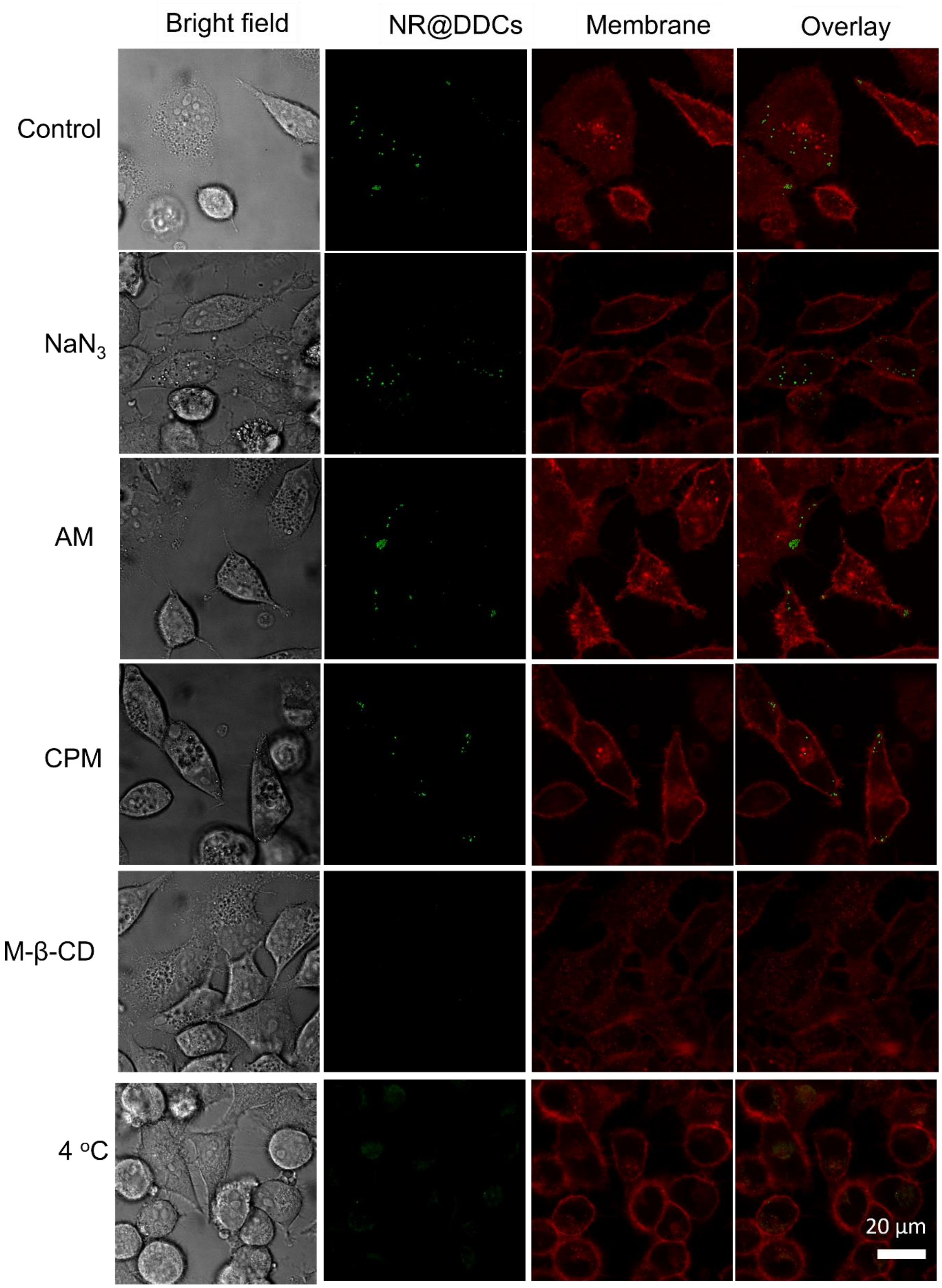

Supplementary Figure 72. Determination of the uptake mechanism. Various endocytosis inhibitors (including chlorpromazine (CPM, 30 µm), amiloride (AM, 20 µm), sodium azide (NaN_3, 100 mM), and methyl-β-cyclodextrin (M-β-CD, 2.5 mM) were pretreated with HeLa cells for 1 hour. Dimerized peptide coacervates (with Nile Red loading, NR@DDCs) were then added and incubated with cells for 2 h. The cells were then washed with cold PBS (3x) and stained

with cell mask for confocal imaging. The presence of chlorpromazine (clathrin-mediated endocytosis inhibitor), amiloride (pinocytosis inhibitor), and sodium azide (energy-dependent endocytosis inhibitor) did not significantly affect the uptake of peptide organelles. However, cells pretreated with methyl-β-cyclodextrin (cholesterol-mediated uptake) showed decreased uptake of coacervated peptides, indicating a significant role of cholesterol-dependent lipid rafting in cellular internalization. Scale bar=20 μm in all microscopy images. Similar results were obtained with 3 samples measured independently.

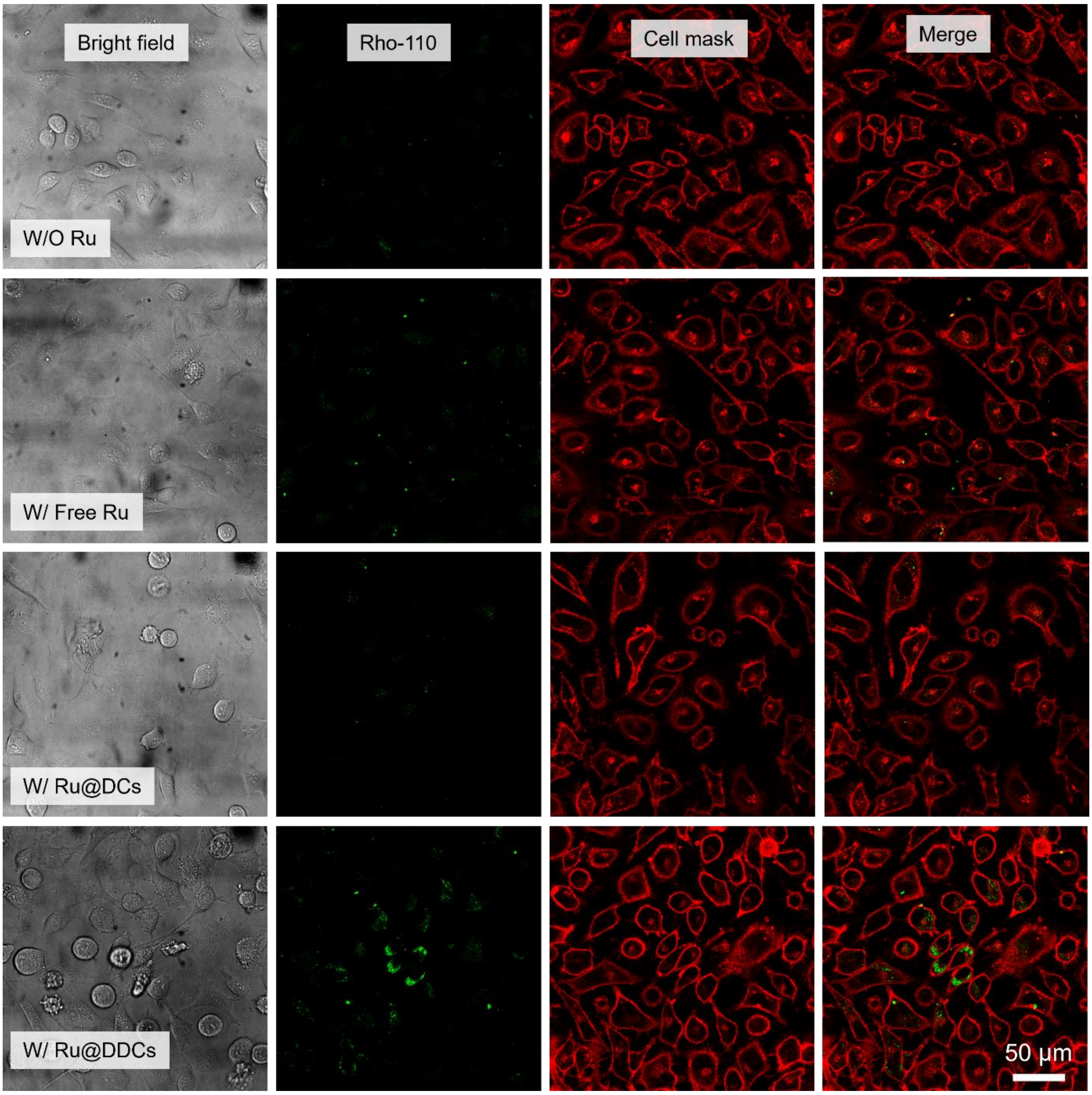

Supplementary Figure 73. Confocal images of HeLa cells after treatment with Ru-organelles showed significant emission in the interior of the cells. The emission was significantly higher than that of the controls (no Ru, free Ru, with Ru integrated with non-treated coacervates). DC: non-treated dipeptide-based coacervate. DDC: dimerized dipeptide-based coacervate. Scale

bar=50 μm in all microscopy images. Similar results were obtained with 3 samples measured independently.

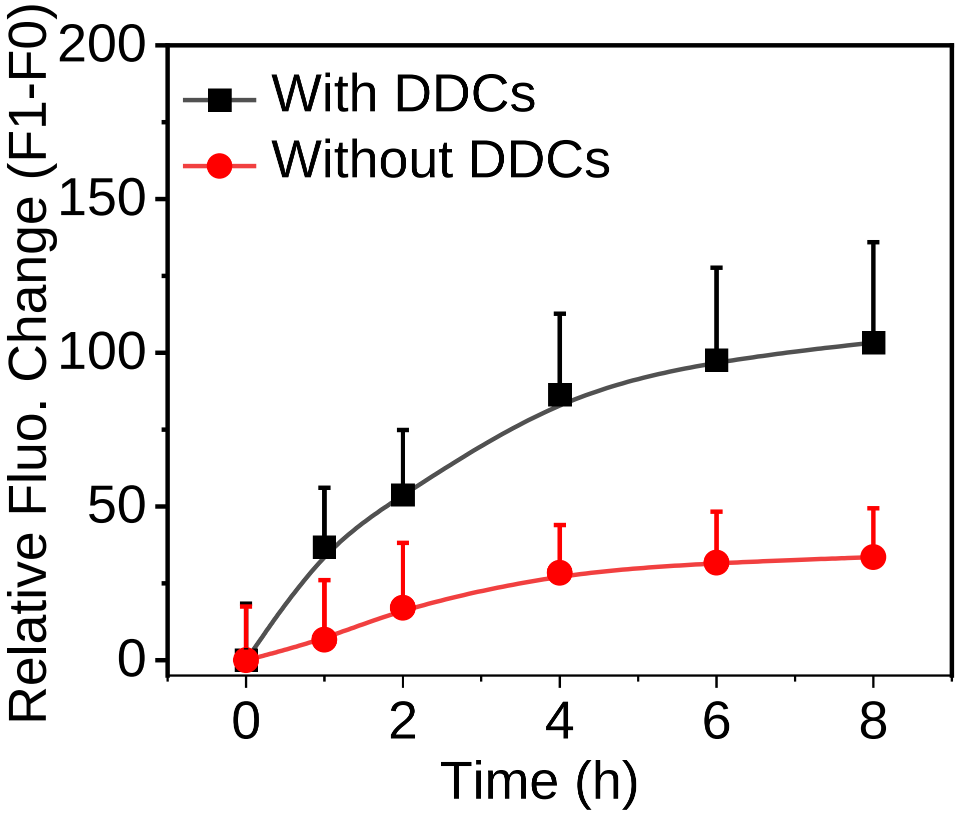

Supplementary Figure 74. Comparison of reaction kinetics of Ru-organelles and free Ru with cells. Ru-organelles and free Ru (concentration 0.01 mg mL^{-1}) were first incubated with cells for 2 h, then washed and fresh medium containing substrate (pro-Rho: 0.01 mg mL^{-1}) was added. The fluorescence intensity of the degraded product was recorded using a microplate reader. Without the presence of Ru organelles (with dimerized dipeptide coacervates, DDCs), there is a decreased increase after 4 hours of incubation. The integration of catalyst with coacervates not only resulted in a more pronounced increase in fluorescence intensity, but also showed a continuous increase after a longer incubation time. Data represent the mean ± SD for n = 3 independent samples.

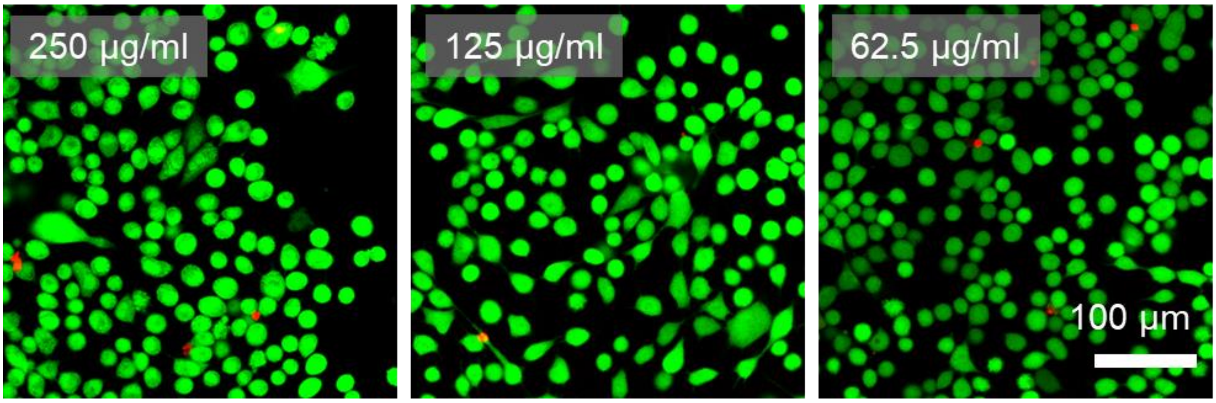

Supplementary Figure 75. Biocompatibility test of stabilized dipeptide coacervates (at different concentrations) with HeLa cells. Cell viability was determined by a live-dead cell imaging assay, with green emission from calcein-am and red emission from propidium iodide. Scale bar=100 μm in all microscopy images. Similar results were obtained with 3 samples measured independently.

3. Supplementary References:

1. Abbas, M., Lipinski, W. P., Nakashima, K. K., Huck, W. T. S., Spruijt, E. A short peptide synthon for liquid-liquid phase separation. *Nat. Chem.* **13**, 1046-1054 (2021).
2. Mu, W., Ji, Z., Zhou, M., Wu, J., Lin, Y., Qiao, Y. Membrane-confined liquid-liquid phase separation toward artificial organelles. *Sci. Adv.* **7**, eabf9000 (2021).
3. Das, R., Landis, R. F., Tonga, G. Y., Cao-Milán, R., Luther, D. C., Rotello, V. M. Control of intra- versus extracellular bioorthogonal catalysis using surface-engineered nanozymes. *ACS Nano* **13**, 229-235 (2019).
4. Mason, A. F., Buddingh', B. C., Williams, D. S., Van Hest, J. C. M. Hierarchical self-assembly of a copolymer-stabilized coacervate protocell. *J. Am. Chem. Soc.* **139**, 17309-17312 (2017).
5. Pijpers, I. a. B., *et al.* Hybrid biodegradable nanomotors through compartmentalized synthesis. *Nano Lett.* **20**, 4472-4480 (2020).
6. Jensen, J. H., Kromann, J. C. The molecule calculator: A web application for fast quantum mechanics-based estimation of molecular properties. *J. Chem. Educ.* **90**, 1093-1095 (2013).
