# Supplementary material for: Dipeptide coacervates as artificial membraneless organelles for bioorthogonal catalysis
Source: Nat Commun. 2024 Jan 2;15:39. doi: 10.1038/s41467-023-44278-9 (PMC10761997; doi:10.1038/s41467-023-44278-9)
Supplement: Supplementary file 3 — Reporting Summary [file 41467_2023_44278_MOESM3_ESM.pdf]

nature portfolio

Corresponding author(s): Katharina Landfester; Lucas Caire da Silva

Last updated by author(s): Nov 20, 2023

Reporting Summary

Nature Portfolio wishes to improve the reproducibility of the work that we publish. This form provides structure for consistency and transparency in reporting. For further information on Nature Portfolio policies, see our Editorial Policies and the Editorial Policy Checklist.

Statistics

For all statistical analyses, confirm that the following items are present in the figure legend, table legend, main text, or Methods section.

n/a	Confirmed	
☐	☒	The exact sample size (*n*) for each experimental group/condition, given as a discrete number and unit of measurement
☐	☒	A statement on whether measurements were taken from distinct samples or whether the same sample was measured repeatedly
☒	☐	The statistical test(s) used AND whether they are one- or two-sided<br>*Only common tests should be described solely by name; describe more complex techniques in the Methods section.*
☒	☐	A description of all covariates tested
☒	☐	A description of any assumptions or corrections, such as tests of normality and adjustment for multiple comparisons
☐	☒	A full description of the statistical parameters including central tendency (e.g. means) or other basic estimates (e.g. regression coefficient) AND variation (e.g. standard deviation) or associated estimates of uncertainty (e.g. confidence intervals)
☒	☐	For null hypothesis testing, the test statistic (e.g. *F*, *t*, *r*) with confidence intervals, effect sizes, degrees of freedom and *P* value noted<br>*Give P values as exact values whenever suitable.*
☒	☐	For Bayesian analysis, information on the choice of priors and Markov chain Monte Carlo settings
☒	☐	For hierarchical and complex designs, identification of the appropriate level for tests and full reporting of outcomes
☒	☐	Estimates of effect sizes (e.g. Cohen's *d*, Pearson's *r*), indicating how they were calculated

Our web collection on statistics for biologists contains articles on many of the points above.

Software and code

Policy information about availability of computer code

Data collection	Nuclear Magnetic Resonance Spectroscopy (NMR): Bruker Avance 400 MHz Ultrashield spectrometer<br>Mass Spectrum:Advion Express CMS compact mass spectrometer and a Waters SYNAPT G2-Si spectrometer<br>Microscope imaging: Leica DMi8 inverted microscope and Leica TCS 264 SP5X system.<br>Dynamic light scattering: Malvern In struments Zetasizer (model Nano ZSP)<br>Microplate reader:TECAN, infinite M1000<br>UV-Vis:Agilent Cary 60 instrument
Data analysis	All numerical data was analyzed and visualized with Origin 2021, 9.8.0.200 Academic (OriginLab)<br>For all microscopy data, the data processing was performed with Leica Application Suite X 3.7.6.25997 and FIJI (ImageJ) version 1.53t were used (https ://imagej .net/Fiji).<br>Mass data was processed with mMass version 5.5.0<br>In the case of NMR the data processing was performed with MestReNova 14.0.0-23239

For manuscripts utilizing custom algorithms or software that are central to the research but not yet described in published literature, software must be made available to editors and reviewers. We strongly encourage code deposition in a community repository (e.g. GitHub). See the Nature Portfolio guidelines for submitting code & software for further information.

Data

Policy information about availability of data

All manuscripts must include a data availability statement. This statement should provide the following information, where applicable:
- Accession codes, unique identifiers, or web links for publicly available datasets
- A description of any restrictions on data availability
- For clinical datasets or third party data, please ensure that the statement adheres to our policy

Full experimental details and data are provided in the Supplementary Information. All data are available from the corresponding author. The data used in the graphs in this study are provided in the Source Data file. Source data are provided with this paper.

Research involving human participants, their data, or biological material

Policy information about studies with human participants or human data. See also policy information about sex, gender (identity/presentation), and sexual orientation and race, ethnicity and racism.

Reporting on sex and gender	Not involving human participants, therefore not applicable.
Reporting on race, ethnicity, or other socially relevant groupings	Not involving human participants, therefore not applicable.
Population characteristics	Not involving human participants, therefore not applicable.
Recruitment	Not involving human participants, therefore not applicable.
Ethics oversight	Not involving human participants, therefore not applicable.

Note that full information on the approval of the study protocol must also be provided in the manuscript.

Field-specific reporting

Please select the one below that is the best fit for your research. If you are not sure, read the appropriate sections before making your selection.

[x] Life sciences [ ] Behavioural & social sciences [ ] Ecological, evolutionary & environmental sciences

For a reference copy of the document with all sections, see nature.com/documents/nr-reporting-summary-flat.pdf

Life sciences study design

All studies must disclose on these points even when the disclosure is negative.

Sample size	All sample sizes are indicated in the figure legends. Experiments were performed in triplicate to ensure reproducibility, which is generally acceptable and sufficient to detect significant differences. The detailed information on sample size was mentioned in the figure legends, method and source data file. No statistical method was used to determine sample size.
Data exclusions	No data was excluded from this study.
Replication	All experiments were repeated at least three times, and all are reproducible following the detailed experimental proceduresdescribed in the main text or in the Supplementary Information.
Randomization	Randomization was not relevant because the experiments were designed to demonstrate reproducibility under controlled conditions.
Blinding	Blinding was not applicable as the results were obtained from physical measurements validated by reproducibility.

Reporting for specific materials, systems and methods

We require information from authors about some types of materials, experimental systems and methods used in many studies. Here, indicate whether each material, system or method listed is relevant to your study. If you are not sure if a list item applies to your research, read the appropriate section before selecting a response.

Materials & experimental systems

n/a	Involved in the study
☒	☐ Antibodies
☐	☒ Eukaryotic cell lines
☒	☐ Palaeontology and archaeology
☒	☐ Animals and other organisms
☒	☐ Clinical data
☒	☐ Dual use research of concern
☒	☐ Plants

Methods

n/a	Involved in the study
☒	☐ ChIP-seq
☒	☐ Flow cytometry
☒	☐ MRI-based neuroimaging

Eukaryotic cell lines

Policy information about cell lines and Sex and Gender in Research

Cell line source(s)	HeLa cells obtained from DSMZ (Deutsche Sammlung von Mikroorganismen and Zellkulturen, Germany). Cat. # ACC 57.
Authentication	No authentication was performed.
Mycoplasma contamination	The cell line was tested for mycoplasma contamination. No mycoplasma contamination was found.
Commonly misidentified lines (See ICLAC register)	No commonly misidentified cell lines were used.
